# ﻿An updated checklist of the wild silkmoths (Lepidoptera, Saturniidae) of Colombia

**DOI:** 10.3897/zookeys.1178.72084

**Published:** 2023-09-07

**Authors:** Lorenzo Comoglio, Ronald Brechlin

**Affiliations:** 1 Laboratorio de Zoología y Ecología Acuática (LAZOEA), Universidad de los Andes, Bogotá, Colombia Universidad de los Andes Bogotá Colombia; 2 Friedberg 20, D-17309 Pasewalk, Germany Unaffiliated Pasewalk Germany

**Keywords:** Bombycoidea, distribution, endemism, Neotropic, South America

## Abstract

In recent years, the study of wild silkmoths (Lepidoptera: Saturniidae) has increased exponentially due to the intense sampling effort and the use of molecular evidence for species delimitation, which led to the description of numerous new taxa especially from the Neotropic. Given these rapid advances, the checklist of the Colombian Saturniidae needs to be updated to cover the taxonomy, distribution, and diversity of these moths in the country. After an extensive review of literature, data repositories, and collections, an updated and comprehensive list of Saturniidae from Colombia is presented, including their occurrence status in each Colombian department. The checklist includes 7 subfamilies, 55 genera, and 790 taxa (766 in species rank) of Saturniidae in Colombia. Current distribution data show that the genus *Winbrechlinia*, the subgenusDarylesia, 379 species, and 18 subspecies are endemic to Colombia. Moreover, a dichotomic key to the Colombian subfamilies is provided. A few taxonomic changes are proposed based on a thorough taxonomic revision of the Colombian taxa. This revision also addresses the issue of outdated species names reported in the first checklist of Colombian Saturniidae (Amarillo-Suárez 2000) and excludes old records of taxa that are considered dubious for Colombia based on new evidence. By presenting an updated list of Colombian species, including the newly described taxa, this study aims at eliminating confusion stemming from outdated names and provides a useful resource for researching and conservating Saturniidae in Colombia. We wish to offer a common reference for future studies on the biodiversity and biogeography of moths in the Neotropical realm.

## ﻿Introduction

“This great diversity of entirely American groups in the Saturniidae […] suggests that the group as a whole arose in the Western Hemisphere and no doubt in the American tropics.”

(Michener 1952: 371)

The Saturniidae, known as wild silkmoths, represent the largest family within the Bombycoidea superfamily and are found almost worldwide (Kitching et al. 2018). However, they are most diverse in the Neotropic region, where they originated (Regier et al. 2008; Rougerie et al. 2022). A global list of valid names for Saturniidae includes 3,454 species in 180 genera (Kitching et al. 2018), of which nearly 2,400 species are estimated to occur in the Neotropic (Decaëns et al. 2021). In the Neotropic, the Saturniidae family is divided into seven subfamilies: Arsenurinae, Ceratocampinae, Cercophaninae, Hemileucinae, Hirpidinae, Oxyteninae, and Saturniinae, the latter being the only cosmopolitan, while the others are exclusively from the New World (Lemaire and Minet 1998; Rougerie et al. 2022). Lemaire revised four subfamilies in America: Saturniinae (Lemaire 1978b), Arsenurinae (Lemaire 1980), Ceratocampinae (Lemaire 1988b), and Hemileucinae (Lemaire 2002). Before publishing his latest three-volume monograph (Lemaire 2002), he compiled a preliminary list of 921 species for the Neotropic (Lemaire 1996). Amarillo-Suárez (2000) reported a total of 183 species in the first checklist of the Colombian Saturniidae, underestimating the diversity of the Hemileucinae, which was later revised by Lemaire (2002). However, both Lemaire (1996) and Amarillo-Suárez (2000) excluded the subfamilies Cercophaninae and Oxyteninae from their checklists. These most basal subfamilies were described as families by Jordan (1924) and then assigned to Saturniidae based on morphological characters (Minet 1994), later confirmed by phylogenetic evidence (Regier et al. 2008). Furthermore, the diversity of Cercophaninae and Oxyteninae has increased enormously due to recent species descriptions (Brechlin and Meister 2014b; Brechlin et al. 2014; Brechlin 2020e, 2021j, 2021o, 2021e, 2023i).

Despite the growing interest and popularity of the Saturniidae (Janzen 1984; Howse and Wolfe 2011; Meister 2011; Basset et al. 2017; Rubin et al. 2018), the literature dealing with the distribution of Colombian Saturniidae is limited. A few ecological studies included lists for specific Colombian localities: Río Ñambí Natural Reserve, Barbacoas, Nariño (Amarillo-Suárez 1997a); San José del Palmar, Chocó (Decaëns et al. 2003b); Tambito Reserve, El Tambo, Cauca (Muñoz and Amarillo-Suárez 2010); Gorgona Island National Park, Guapí, Cauca (Calero-Mejía et al. 2014); and Utría National Park, Chocó (Prada-Lara et al. 2019) in the Chocó biogeographic region; Albania, Caquetá, in the Orinoquía region (Racheli and Vinciguerra 2005); and Arcabuco and Quipama, Boyacá, in the Andean region (Decaëns et al. 2007). However, these local checklists have lost their validity due to recent taxonomic advances.

In the last decade, approximately 1,500 new species and subspecies of Saturniidae have been described globally (Kitching et al. 2018). This enormous number is mainly due to DNA studies (Hebert et al. 2003; Padial et al. 2010). Despite being controversially discussed (Will and Rubinoff 2004; Will et al. 2005; Peigler 2013), DNA barcoding is now widely recognized as a tool for revealing cryptic Lepidoptera species (Decaëns and Rougerie 2008; Vaglia et al. 2008; Gibbs 2009; Hausmann et al. 2009; Decaëns et al. 2021; Moraes et al. 2021). Today, integrative taxonomy combines morphological features, geographic distribution, COI barcode studies (Silva-Brandão et al. 2009 provide an extensive review on the subject), and nuclear markers to increase resolution (Rougerie et al. 2012). Unsurprisingly, many of the newly described Saturniidae are distributed in Colombia due to the variety of ecosystems (van der Hammen and Rangel 1997), biodiversity hotspots (Myers et al. 2000), and the recent sampling boosting, especially at high elevations, in previously inaccessible localities, given the limitations due to the control of the territory by various armed groups (Brechlin 2016f; Murillo-Sandoval et al. 2020). Since 2008, many descriptions of neotropical Saturniidae have been published in the Entomo-Satsphingia journal, including two major revisions of the genera *Hylesia* Hübner, 1820 (Brechlin et al. 2016a) and *Janiodes* Jordan, 1924 (Brechlin 2020e). The most striking result was the description of the genus *Winbrechlinia* Brechlin, 2016, endemic to the cloud forests and páramos of the Sierra Nevada of Santa Marta in northern Colombia (Brechlin 2016f, 2018m, 2020d).

The geographical complexity of Colombia makes its fauna extraordinarily diverse and highly endemic (Bernal and Lynch 2008; Avendaño et al. 2021; Bota-Sierra et al. 2021; Pérez-Escobar et al. 2022). The topography is characterized by three main parallel Andean mountain ranges, known as Cordilleras, located within the “Tropical Andes” biodiversity hotspot (Myers et al. 2000). The Andean region is bordered to the east by the Orinoquía and Amazon regions and to the west by the Chocó biogeographic region, another biodiversity hotspot (Myers et al. 2000). The three Andean mountain ranges are separated by the two large, major streams of Colombia, the Cauca River, which flows northward between the Western and Central Cordilleras, and the Magdalena River, which divides the Central and Eastern Cordilleras. After emerging from the Colombian Massif, these two rivers join and descend to the Caribbean Sea. On the Caribbean’s margin there is the Sierra Nevada de Santa Marta, whose highest elevations are the tallest peaks (5775 m) in Colombia. This more recently formed area (Gómez et al. 2021) is also an essential hotspot of biodiversity that hosts many endemic species (Myers et al. 2000; Brechlin 2016f). The geological history of Colombia has allowed the discontinuous isolation of species, favoring allopatric speciation (Vargas et al. 2023) through the increase in the availability of new niches (Purser 2015; Hazzi et al. 2018). Notably, páramos have been described as “islands” with flickering connectivity (Flantua et al. 2019) and the fastest diversification rate (Madriñán et al. 2013). For these geographical reasons, Colombia probably has one of the most diverse and endemic Saturniidae faunas in tropical America (Lemaire and Venedictoff 1989; Decaëns and Rougerie 2008; Jiménez-Bolívar et al. 2021).

In light of the advances made in the last two decades, the first checklist of the Colombian Saturniidae (Amarillo-Suárez 2000) is outdated. No systematic work has been carried out to date to review the taxonomy and distribution of the Colombian Saturniidae taxa, considering the contributions of checklists for some specific Colombian localities and including the vast cryptic diversity revealed by the use of DNA barcoding and the recent tremendous sampling effort. At the subfamilies level, the diversity of Cercophaninae and Oxyteninae has previously been underestimated since the contribution of these subfamilies is reviewed here for the first time, taking as a starting point the preliminary checklist by Comoglio and Brechlin (2021). Likewise, considering the separation of Hirpidinae from Hemileucinae (Rougerie et al. 2022), updating the taxonomic key for subfamilies is necessary.

This work aims to present an updated checklist of the known Saturniidae from Colombia, considering the many new descriptions and revalidation of taxa, and to clarify the taxonomic confusion that these taxonomic advances may have produced. An updated dichotomous key was also elaborated for the subfamilies distributed in the country. Furthermore, a few taxonomic changes are proposed and discussed, following the criteria of delimitation of species currently used in the literature (Brechlin and Meister 2011a, 2011c; Brechlin et al. 2016a; Bénéluz 2021; Brechlin 2022i). Based on an extensive review of literature, data repositories, and collections, this checklist is the first one for Colombia (published on 6 August 2021 as a preprint) which also includes the subfamilies Cercophaninae and Oxyteninae, and that extensively covers the Hemileucinae. Species and subspecies considered endemic for the country are highlighted here, thanks to an extensive review of the distribution data of all the Neotropical taxa considered in this study. Taxa excluded from our Colombian checklist are discussed in detail, comparing the present knowledge with the old records found in the literature. In addition, taxa with potential distribution in Colombia, but whose presence has not been confirmed yet, are discussed on the basis of their current distribution data. After the preprint of this study (Comoglio and Brechlin 2021) was made available, another checklist of the Colombian Saturniidae was published by Jiménez-Bolívar et al. (2021), whose results are thoroughly reviewed here.

## ﻿Materials and methods

This checklist is mainly the product of recent sampling efforts, which have led to the description of many Saturniidae taxa for Colombia, and a literature review of articles, species descriptions, taxonomic revisions, and records available on BOLD (Hebert and Ratnasingham 2007) until 15 June 2023. In addition, both authors contributed with data from the collections they are currently curating to add new records which have not been previously published elsewhere. The first author examined specimens in the Entomological Collection of the “C.J. Marinkelle” Natural History Museum, Universidad de los Andes, Bogotá, Colombia (**ANDES-E**). The second author conducted a comprehensive review of his Research Collection, Pasewalk, Germany (**CRBP**), which includes type material of more than 530 Colombian taxa and is extensively barcoded (“BC-RBP” in BOLD). The specimens deposited in the main national collections, such as the Institute of Natural Sciences, National University of Colombia, Bogotá (**ICN-MHN**); the “Francisco Luis Gallego” Entomological Museum, National University of Colombia, Medellín (**MEFLG**); the Javeriano Museum of Natural History “Lorenzo Uribe, S.J.”, Pontificia Universidad Javeriana, Bogotá (**MPUJ**), and the National Taxonomic Collection of Insects “Luis María Murillo”, Mosquera (**CTNI**), were examined by Amarillo-Suárez (2000), Clavijo-Giraldo and Uribe (2019), and Jiménez-Bolívar et al. (2021). However, some of these collection records are provided ignoring the current nomenclature that is extensively reviewed and discussed in this paper. Some interesting records are found in the entomological collection of the Alexander von Humboldt Institute, Villa de Leyva, Colombia (IAvH-E), and have DNA barcodes in BOLD for some additional taxa. Barcodes are essential for the verifiable identification of specimens, especially those belonging to species complexes (Decaëns and Rougerie 2008; Decaëns et al. 2021). The implications of using raw data available in repositories without curation or expert identification, such as some records shown in Jiménez-Bolívar et al. (2021), have been debatable (Zizka et al. 2020) and are also discussed in this work, which is also a tool to improve the identification of Colombian Saturniidae taxa.

The dichotomous key to subfamilies covers only Colombian species (e.g., *Janiodes* species for the Cercophaninae). It is based on morphological studies of adults achieved by Michener (1952) and Lemaire and Minet (1998), with the addition of the analysis of the adult morphology of the Arsenurinae (de Camargo et al. 2009), Ceratocampinae (Balcázar-Lara and Wolfe 1997), Cercophaninae (Jordan 1924; Minet 1994), Hemileucinae (Lemaire 2002), and Hirpidinae (Rougerie et al. 2022). The morphological characters used in the literature were systematized and corroborated by direct examination of the specimens in the reviewed collections. Finally, an updated dichotomous key for Colombia was created, modifying the last global subfamily key presented by Lemaire and Minet (1998) and including the recently described subfamily Hirpidinae (Rougerie et al. 2022).

The taxa’s higher classification, names, and authority follow the Bombycoidea global checklist (Kitching et al. 2018) with some additions due to the most recent descriptions and revalidation of old taxa names found in the literature, and especially the recent phylogenomic analysis by Rougerie et al. (2022). The main list is shown alphabetically, ordered by subfamilies, tribes, subtribes, genera, subgenera, species, and subspecies. The occurrence in each Colombian department is provided for each species and subspecies. The Colombian departments are abbreviated as follows: Amazonas (Am), Antioquia (An), Arauca (Ar), Boyacá (By), Caldas (Cl), Caquetá (Ca), Casanare (Cn), Cauca (Cc), Cesar (Ce), Cundinamarca (Cu), Chocó (Ch), Guainía (Gn), Guaviare (Gv), Huila (Hu), La Guajira (Gj), Magdalena (Ma), Meta (Me), Nariño (Na), Norte de Santander (NS), Quindío (Qu), Putumayo (Pu), Risaralda (Ri), Santander (St), Tolima (To), Vaupés (Va), Valle del Cauca (Vl), and Vichada (Vi). Endemic species and subspecies are highlighted. Taxa previously reported in Colombia that were excluded from the list are discussed separately. According to their current distribution data, a list of taxa with potential occurrence in Colombia is also presented.

## ﻿Results

A total of 843 specimens in ANDES-E and more than 15,000 specimens in CRBP were examined, and 2,854 barcodes of Colombian Saturniidae specimens were retrieved from BOLD. To show the updated checklist, it is necessary to propose some taxonomic changes that are summarized here and then discussed, compared with previous studies, and interpreted in light of the criteria currently used to delimit species. An identification key for the 7 Saturniidae subfamilies distributed in Colombia is also presented as a result of this review. An updated national list for the family is then presented, including the distribution of the taxa in the Colombian departments, highlighting the endemic species, and reporting the current evidence as a barcode and related bibliographic references. The taxa excluded from this updated list are discussed below, comparing our results with the old records found in the literature. In addition, species with a possible distribution in Colombia, whose presence has not yet been confirmed, are discussed based on their current distribution data.

During the taxonomic review carried out in this study, we found taxa whose delimitation criteria between species have lost their validity and taxa that need to be revalidated conservatively, according to current knowledge. The proposed taxonomic changes are reflected in the checklist, and their validity and interpretation are discussed in detail below. The proposed taxonomic changes are summarized here. The following taxa, hitherto treated as subspecies, are raised to species status: *Arsenuralemairei* L. Racheli & T. Racheli, 1998, stat. nov. from *A.thomsoni* Schaus, 1906, *Copiopteryxbanghaasi* Draudt, 1930, stat. nov. from *C.semiramis* (Cramer, 1775), and *Rhescyntisnorax* Druce, 1897, stat. nov. from *R.hippodamia* Druce, 1897. A new combination is proposed in this context: *Copiopteryxbanghaasiandensis* (Lemaire, 1974), comb. nov. In addition, *Bathyphlebiaagliagschwandneri* Schawerda, 1925, stat. nov. is removed from its synonymy with *B.a.aglia* C. Felder & R. Felder, 1874 and here treated as a subspecies of the latter. *Grammopeltacervina* Rothschild, 1907, stat. rev. and *Copaxaignescens* Lemaire, 1978, stat. rev. are reinstated and removed from their current synonymies with *G.lineata* (Schaus, 1906) and *C.niepelti* Draudt, 1929, respectively. Furthermore, *Rothschildiaequatorialisbogotana* Rothschild, 1907, stat. rev., comb. nov. is reinstated as a subspecies, but now of *equatorialis* Rothschild, 1907 instead of *orizaba* (Westwood, 1853). A new synonymy is proposed: *Rhescyntishippodamiacolombiana* (Bouvier, 1927), syn. nov. is now treated as a subjective junior synonym of *R.norax* Druce, 1897.

### ﻿Key to subfamilies

A dichotomous key for the seven subfamilies of Saturniidae found in Colombia is presented below, excluding the genera of these subfamilies not found until now in Colombia. The mentioned characters account only for the external morphology. Therefore, identification at the subfamily level is generally immediate.

**Table:** 

1	Male with antennal flagellum dorsally scaled to the apex and lateroventral orientation of the rami; bipectinate antennae in both sexes; proboscis present (Fig. 1A)	**Oxyteninae Jordan, 1924**
–	Male with antennal flagellum unscaled, at least for most of its length; lateral or laterodorsal orientation of the rami	**2**
2	Hindwing with strongly indicated crossvein (R) between Sc and upper edge of the discal cell; ventrally spined tarsi; forewings with at least one dark discocellular spot; small to medium size	**3**
–	Hindwing with crossvein nearly always absent or faintly indicated; hindwings with tails, hyaline discal spots, or eyespots; proboscis always absent	**4**
3	Yellow proboscis, strong and coiled up; bipectinate antennae in males, simple in females; males with a large foretibial epiphysis, whose median area looks internally notched; butterfly-like body shape and pierid-like wing shape (Fig. 1B)	**Cercophaninae Jordan, 1924**
–	Proboscis absent; orange quadripectinate antennae in males, simple in females; frons slightly convex at sides; orange-brown coloration	**Hirpidinae Rougerie, 2022**
4	Presence of solid bristles on pilifers or the clypeal margin between pilifers; hindwings usually with tails, longer in males; dull brown coloration; medium to large size (Fig. 1C)	**Arsenurinae Jordan, 1922**
–	Pilifers and clypeal margin without bristles	**5**
5	Frons convex at sides so that lateral sutures are hidden in an anterior view and antennal cones (short ventral protuberances on flagellomeres) simple; distal section of antenna devoid of rami; general body shape sphingid-like (Fig. 1D)	**Ceratocampinae Harris, 1841**
–	Frons flat at sides or, if convex, antennal cones multiple	**6**
6	Antennae, when quadripectinate, with bases of rami invariably well separated; thorax with the anterior area of mesoscutum lacking middorsal projection; in the forewing, when the discal cell is closed, the base of M1 arising closer to M2 than Rs or about midway between M2 and Rs; segments of labial palpi not fused; hyaline discal spots on both forewings and hindwings (Fig. 1E)	**Saturniinae Boisduval, 1837**
–	Antennae, when quadripectinate, with apical ramus of a segment usually adjacent to the basal ramus of next segment; if rami separate, mesoscutum with an anterior middorsal projection or forewing with the base of M1 distinctly closer to Rs than to M2 (or even stalked with Rs); antennal cones present and simple; labial palpi occasionally fused; hindwings usually with eyespots (Fig. 1F, G)	**Hemileucinae Grote & Robinson, 1866**

**Figure 1.**
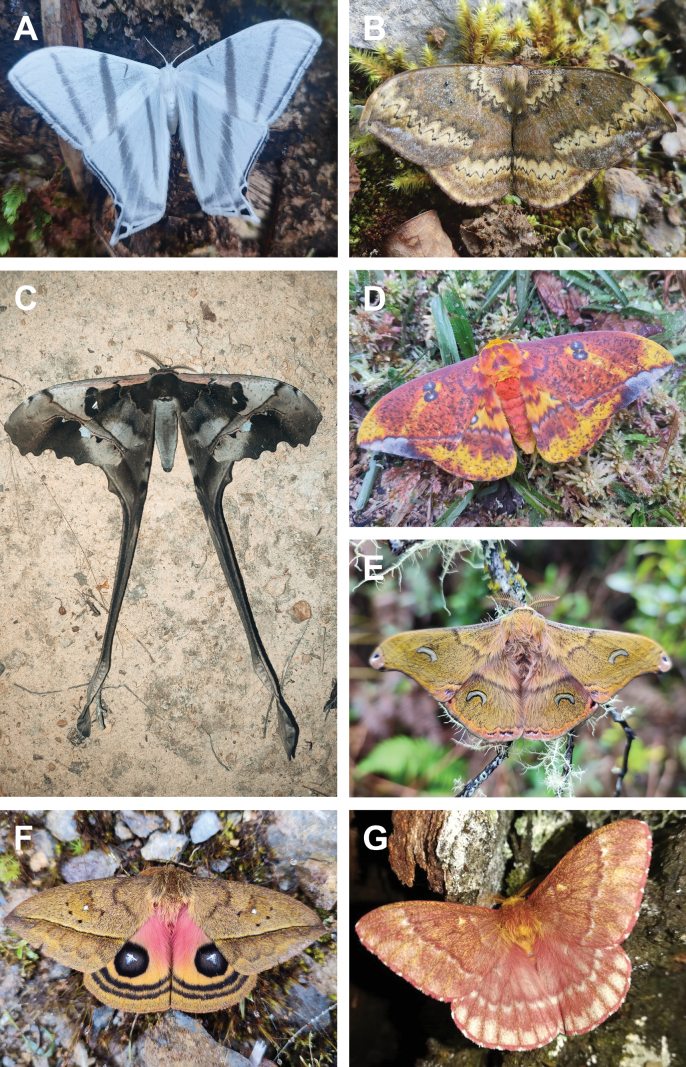
Representative taxa of the diversity of the Colombian Saturniidae subfamilies **A***Theriniaterminalis* (Oxyteninae) **B***Janiodeslavcarchensis* (Cercophaninae) **C***Copiopteryxjehovah* (Arsenurinae) **D***Eaclesniepelti* (Ceratocampinae) **E***Copaxasapatoza* (Saturniinae) **F***Automerisalticarchensis* (Hemileucinae) **G***Winbrechliniasinjaevi* (Hemileucinae).

### ﻿Overview of the checklist

A total of 790 taxa (766 in species rank) into 55 genera of Saturniidae was recorded for Colombia (Tables 1, 2). The most diverse subfamily is Hemileucinae, with 467 species, which also contains the genus with the highest species richness, *Automeris* (86 species), while the least diverse subfamily is Hirpidinae, with eight species of the genus *Hirpida* (Table 1).

**Table 1.** A summary of the number of Colombian Saturniidae genera per subfamily, tribe, and subtribe, and the number of species per genus, together with the number of endemic species.

Taxon	Number of genera	Number of species	Number of endemic species
Family Saturniidae Boisduval, 1837	55	766	379
Subfamily Arsenurinae Jordan, 1922	8	35	1
Tribe Arsenurini Jordan, 1922	8	35	1
Genus *Arsenura* Duncan, 1841		15	
Genus *Caio* Travassos & Noronha, 1968		1	
Genus *Copiopteryx* Duncan, 1841		3	
Genus *Dysdaemonia* Hübner, 1819 [1816]		3	
Genus *Grammopelta* Rothschild, 1907		1	
Genus *Paradaemonia* Bouvier, 1925		6	1
Genus *Rhescyntis* Hübner, 1819 [1816]		3	
Genus *Titaea* Hübner, 1823		3	
Subfamily Ceratocampinae Harris, 1841	15	90	28
Tribe Bathyphlebiini Travassos & Noronha, 1967	3	23	3
Genus *Bathyphlebia* C. Felder & R. Felder, 1874		2	1
Genus *Eacles* Hübner, 1819 [1816]		14	
Genus *Schausiella* Bouvier, 1930		7	2
Tribe Citheroniini Harris, 1841	3	14	3
Genus *Citheronia* Hübner, 1819 [1816]		11	2
Genus *Citheronioides* Lemaire, 1988		2	1
Genus *Procitheronia* Michener, 1949		1	
Tribe Dryocampini Grote & Robinson, 1866	9	53	22
Genus *Adeloneivaia* Travassos, 1940		13	4
Genus *Adelowalkeria* Travassos, 1941		5	1
Genus *Cicia* Oiticica Filho, 1964		1	
Genus *Citioica* Travassos & Noronha, 1965		5	2
Genus *Othorene* Boisduval, 1872		4	
Genus *Psilopygida* Michener, 1949		1	
Subgenus Psigida Oiticica Filho, 1959		1	
Genus *Ptiloscola* Michener, 1949		5	3
Genus *Rachesa* Michener, 1949		4	3
Genus *Syssphinx* Hübner, 1819 [1816]		15	9
Subfamily Cercophaninae Jordan, 1924	1	81	73
Tribe Janiodini Jordan, 1924	1	81	73
Genus *Janiodes* Jordan, 1924		81	73
Subfamily Hemileucinae Grote & Robinson, 1866	24	467	248
Tribe Hemileucini Grote & Robinson, 1866	22	425	229
Subtribe Automeriina Bouvier, 1928	12	269	120
Genus *Automerina* Michener, 1949		6	2
Genus *Automeris* Hübner, 1819 [1816]		86	31
Genus *Catacantha* Bouvier, 1930		2	1
Genus *Erythromeris* Lemaire, 1969		5	4
Genus *Gamelia* Hübner, 1819 [1816]		38	30
Genus *Gamelioides* Lemaire, 1988		8	7
Genus *Hylesia* Hübner, 1820		80	28
Subgenus Darylesia Brechlin, 2022		2	2
Subgenus Hylesia Hübner, 1820		77	26
Subgenus Micrattacus Walker, 1855		1	
Genus *Hylesiopsis* Bouvier, 1929		1	
Genus *Hyperchiria* Hübner, 1819 [1816]		7	2
Genus *Leucanella* Lemaire, 1969		17	10
Genus *Molippa* Walker, 1855		10	1
Genus *Pseudautomeris* Lemaire, 1967		9	4
Subtribe Hemileucina Grote & Robinson, 1866	10	156	109
Genus *Cerodirphia* Michener, 1949		20	15
Genus *Dirphia* Hübner, 1819 [1816]		31	16
Genus *Dirphiella* Michener, 1949		1	
Genus *Dirphiopsis* Bouvier, 1928		5	2
Genus *Meroleuca* Packard, 1904		30	29
Subgenus Dihirpa Draudt, 1929		4	4
Subgenus Meroleuca Packard, 1904		3	3
Subgenus Meroleucoides Michener, 1949		23	22
Genus *Paradirphia* Michener, 1949		15	14
Genus *Periphoba* Hübner, 1820		8	3
Genus *Pseudodirphia* Bouvier, 1928		38	23
Genus *Rhodirphia* Michener, 1949		2	1
Genus *Winbrechlinia* Brechlin, 2016		6	6
Tribe Lonomiini Bouvier, 1930	2	42	19
Genus *Lonomia* Walker, 1855		16	3
Genus *Periga* Walker, 1855		26	16
Subfamily Hirpidinae Rougerie, 2022	1	8	5
Genus *Hirpida* Draudt, 1930		8	5
Subfamily Oxyteninae Jordan, 1924	3	29	1
Genus *Homoeopteryx* C. Felder & R. Felder, 1874		3	1
Genus *Oxytenis* Hübner, 1819 [1816]		18	
Genus *Therinia* Hübner, 1823		8	
Subfamily Saturniinae Boisduval, 1837	3	56	23
Tribe Attacini Blanchard, 1840	1	17	5
Genus *Rothschildia* Grote, 1896		17	5
Tribe Saturniini Boisduval, 1837	2	39	18
Genus *Antheraea* Hübner, 1819 [1816]		1	
Genus *Copaxa* Walker, 1855		38	18

The genus *Winbrechlinia*, the subgenusDarylesia, 379 species (49.5%, almost half of the total), and 18 subspecies are endemic to Colombia (Table 1). More than half of the Hemileucinae (248 species, 53.1%) are endemic, but the Cercophaninae also stand out, with the genus *Janiodes* as the only representative in Colombia, which includes 73 endemic species (90.1%) of a total of 81 (Table 1). The genera *Meroleuca* (96.7% endemic rate), *Paradirphia* (93.3%), and *Gamelioides* (87.5%) present the highest rates of endemism. On the other hand, for both Arsenurinae and Oxyteninae, only one species of each is endemic to Colombia until now, making them the subfamilies with the lowest rates of endemism (2.9% and 3.4%, respectively).

## ﻿Checklist

**Table 2.** Main checklist of Colombian Saturniidae. Endemic (End.) species or subspecies are marked with a plus sign (+) or a section sign (§), respectively. Distribution data are given for each Colombian department. Occurrence records marked with a question mark (?) were found in the literature and considered doubtful since recent samplings could not confirm them. Those taxa with their type locality in Colombia are shown with their primary evidence labeled as “TL”. The additional evidence is mostly barcode numbers that refer to “Sample ID” in BOLD and are provided for most taxa, especially for type specimens and the most taxonomically cryptic species group. Many records refer to specimens found in ANDES-E and/or CRBP collections. References for old records that have been found in the literature are also provided.

Taxon	End.	Distribution	Evidence	References
Family Saturniidae Boisduval, 1837				
Subfamily Arsenurinae Jordan, 1922				
Tribe Arsenurini Jordan, 1922				
Genus *Arsenura* Duncan, 1841				
*Arsenuraalbopicta* Jordan, 1922		Am, Cn, Gn, Pu	BC-Dec1510	Amarillo-Suárez 2000; ANDES-E, CRBP
*Arsenuraarcaei* Druce, 1886		An, Ch, Vl	BC-RBP 12178	Lemaire 1980; Amarillo-Suárez 2000 as *A.batesii*; Decaëns et al. 2003b; Brechlin 2023b; CRBP
*Arsenuraarchianassaarchianassa* Draudt, 1930		An, Ch, Vl	TL; BC-RBP 12184	Draudt 1929; Lemaire 1980; Decaëns et al. 2003b; CRBP
*Arsenuraarchianassavenecolombiana* Brechlin, 2023		By, Cu, To	TL; BC-RBP 12186	Decaëns et al. 2007 as *A.armida*; Brechlin 2023b; ANDES-E, CRBP
*Arsenuraarianae* Brechlin & Meister, 2010		Ma	BC-RBP 12187	CRBP
*Arsenuraarmida* (Cramer, 1779)		Am, Ca, Cn, Me	BC-RBP 12185	Amarillo-Suárez 2000; CRBP
*Arsenurabatesii* (C. Felder & R. Felder, 1874)		Am, Cu, Me	BC-Dec0471	Amarillo-Suárez 2000; ANDES-E, CRBP
*Arsenurabeebei* (Fleming, 1945)		Gn		ANDES-E
*Arsenuraciocolatina* Draudt, 1930		An, By, Ca, Cu, Gv, Ma, Me	TL; BC-RBP 4009	Draudt 1929; Lemaire 1980; Amarillo-Suárez 2000; ANDES-E, CRBP
*Arsenurafuscata* Brechlin & Meister, 2010		Cc	BC-RBP 12188	Brechlin and Meister 2010g; CRBP
*Arsenurakaechi* Brechlin & Meister, 2010		Ca, Hu	BC-RBP 11142	Brechlin and Meister 2010a; CRBP
*Arsenuralemairei* L. Racheli & T. Racheli, 1998, stat. nov.		Cn	BC-FMP-1420	CRBP
*Arsenuramossi* Jordan, 1922		Am, Me		ANDES-E, CRBP
*Arsenuraponderosaponderosa* Rothschild, 1895		Cc	BC-RBP 10988	CRBP
*Arsenurarebeli* Gschwandner, 1920		Cc		CRBP
*Arsenurasyllasylla* (Cramer, 1779)		Am, Pu	BC-RBP 12631	CRBP
*Arsenurasyllaniepelti* (Schüssler, 1936)		Ch, Vl	TL	Schüssler 1936; Amarillo-Suárez 2000; CRBP
Genus *Caio* Travassos & Noronha, 1968				
*Caiochampioni* (Druce, 1886)		An, By, Ch, Cu, Na, To, Vl	BC-RBP 10113	Lemaire 1980; Amarillo-Suárez 2000; Decaëns et al. 2007; ANDES-E, CRBP
Genus *Copiopteryx* Duncan, 1841				
*Copiopteryxbanghaasiandensis* (Lemaire, 1974), comb. nov.		An, By, Ch, St, Vl	BC-Dec0058	Decaëns et al. 2003b and 2007 as *C.semiramisandensis*; ANDES-E, CRBP
*Copiopteryxjehovah* (Strecker, 1874)		Ca, Cn, Pu	BC-Dec1443	Lemaire 1980; Amarillo-Suárez 2000; CRBP
*Copiopteryxsemiramissemiramis* (Cramer, 1775)		Am, Ca, Cu, Me, Pu	BC-RBP 9458	Amarillo-Suárez **2000** as *C.semiramis*; ANDES-E, CRBP
Genus *Dysdaemonia* Hübner, 1819 [1816]				
*Dysdaemoniaaustraloboreas* Brechlin & Meister, 2009		Am, Cc	BC-RBP 11594	Brechlin and Meister 2009; CRBP
*Dysdaemoniapanamana* Brechlin, 2019		An, By, Ma	BC-RBP-2309	Brechlin 2019e; ANDES-E, CRBP
*Dysdaemoniavanschaycki* Brechlin, 2019		Gj, Me	TL; BC-RBP 11417	Brechlin 2019e; CRBP
Genus *Grammopelta* Rothschild, 1907				
*Grammopeltacervina* Rothschild, 1907, stat. rev.		By, Ca, Ch, Cu, Vl	BC-RBP 10352	Lemaire 1980, Amarillo-Suárez 2000, Decaëns et al. 2003b, and 2007 as *G.lineata*; ANDES-E, CRBP
Genus *Paradaemonia* Bouvier, 1925				
*Paradaemoniacastanea* (Rothschild, 1907)		Ch, Vl	BC-RBP 12255	Amarillo-Suárez 2000 as *P.platydesmia*; CRBP
*Paradaemoniaiscaybambensis* Brechlin & Meister, 2013		Ca	BC-RBP 12632	Brechlin and Meister 2013b; CRBP
*Paradaemonianycteris* (Jordan, 1922)		Ar, Cn, Me	BC-Dec1438	Amarillo-Suárez 2000; CRBP
*Paradaemoniaplatydesmia* (Rothschild, 1907)		Am, Ca, Me, Va	BC-RBP 8295	Lemaire 1980 and Amarillo-Suárez 2000 as *P.andensis*; ANDES-E, CRBP
*Paradaemoniasambasambdensis* Brechlin & Meister, 2012		Am, Ca	BC-Dec1778	Amarillo-Suárez 2000 as *P.samba*; CRBP
*Paradaemoniasinjaevi* Brechlin, 2018	+	St	TL; BC-RBP 10108	Brechlin 2018b; CRBP
Genus *Rhescynti*s Hübner, 1819 [1816]				
*Rhescyntishermeshermandensis* Brechlin & Meister, 2013		Pu		Brechlin and Meister 2013a; CRBP
*Rhescyntishippodamia* (Cramer, 1777)		Am, By, Ca, Pu	BC-Dec1575	Amarillo-Suárez 2000 as “*R.hippodamina*”; ANDES-E, CRBP
*Rhescyntisnorax* Druce, 1897, stat. nov.		Ch, Na, Ri, Vl	BC-EvS 3312	Amarillo-Suárez 2000 and Prada-Lara et al. 2019 as *R.hippodamia*; Decaëns et al. 2003b as *R.hippodamiacolombiana*; ANDES-E, CRBP
Genus *Titaea* Hübner, 1823				
*Titaealemoulti* (Schaus, 1905)		Am, Ca, Me	IAvH-E-190496	Lemaire 1980; Amarillo-Suárez 2000; CRBP
*Titaeatamerlanamazonensis* Lemaire, 1980		By, Ca, Gj	BC-RBP 8679	Lemaire 1980; CRBP
*Titaeatamerlannobilis* (Schaus, 1912)		Am, An, By, Ca, Ch, Gn, Na, Vl	BC-RBP-2316	Lemaire 1980; Amarillo-Suárez 2000; Decaëns et al. 2003b; Prada-Lara et al. 2019; ANDES-E, CRBP
*Titaeatimur* (Fassl, 1915)		Am, Cn, Me	TL; BC-Dec0098	Fassl 1915; Amarillo-Suárez 2000; ANDES-E, CRBP
Subfamily Ceratocampinae Harris, 1841				
Tribe Bathyphlebiini Travassos & Noronha, 1967				
Genus *Bathyphlebi*a C. Felder & R. Felder, 1874				
*Bathyphlebiaagliaaglia* C. Felder & R. Felder, 1874	+	By, Cu, Me, NS, Pu, Qu, St	TL; BC-RBP 10815	Felder and Felder 1874; Amarillo-Suárez 2000; Mielke and St Laurent 2021; ANDES-E, CRBP
*Bathyphlebiaagliagschwandneri* Schawerda, 1925, stat. nov.	§	An, To	TL; BC-RBP 8363	Schawerda 1925; Lemaire 1988b; ANDES-E, CRBP
*Bathyphlebiaeminens* (Dognin, 1891)		An, By, Cl, Cu, Qu, Ri, St, Vl	BC-RBP 8434	Amarillo-Suárez 2000; Decaëns et al. 2007; ANDES-E, CRBP
Genus *Eacles* Hübner, 1819 [1816]				
*Eaclesadoxandensis* Brechlin, 2022		Ca	BC-MNHN0322	Amarillo-Suárez 2000 and Racheli and Vinciguerra 2005 as *E.adoxa*; Brechlin 2022i; CRBP
*Eaclesanchicayensis* Lemaire, 1971		An, By, Ce, Ch, Cl, Cu, Gj, Ma, St, Vl	TL; BC-RBP 8322	Lemaire 1971a; Decaëns et al. 2003b and Prada-Lara et al. 2019 as *E.imperialisanchicayensis*; Decaëns et al. 2007 as *E.imperialiscacicus*; ANDES-E, CRBP
*Eaclesbarnesi* Schaus, 1905		An, Ca, Pu		Amarillo-Suárez 2000; CRBP
*Eaclesbarragani* Brechlin & Käch, 2015		Na		Brechlin and Käch 2015; CRBP
*Eacleseccolombiana* Brechlin, 2022		Pu	Paratype	Brechlin 2022i; CRBP
*Eaclesfulvasteroriecuadoriana* Brechlin & Meister, 2011		Am, Ca, Me	RROU00477	Racheli and Vinciguerra 2005 as *E.fulvaster*; ANDES-E, CRBP
*Eaclesguianensisguiaandensis* Brechlin, 2022	§	Cc, Pu	TL; BC-RBP 10991	Brechlin 2022i; ANDES-E, CRBP
*Eaclesimpandensis* Brechlin, 2022		Am, Ca, Cc, Me	BC-RBP 12140	Racheli and Vinciguerra 2005 as *E.imperialiscacicus*; Brechlin 2022i; CRBP
*Eaclesjohnsoniella* Oiticica Filho & Michener, 1950		Cu, St	TL; BC-RBP 10037	Oiticica Filho and Michener 1950b; Brechlin 2017f; ANDES-E, CRBP
*Eacleskaechi* Brechlin & Meister, 2011		Cc	BC-RBP 12180	Brechlin and Meister 2011c; CRBP
*Eaclesniepelti* Draudt, 1930		Cc, Ch, Na, Ri, Vl	TL; BC-FMP-1019	Amarillo-Suárez 2000, Decaëns et al. 2003b, and Prada-Lara et al. 2019 as *E.ormondeiniepelti*; ANDES-E, CRBP
*Eaclespenelope*﻿ (Cramer, 1775)		Ca, Ch, Gv, Me, To, Vl	BC-RBP 6000	Amarillo-Suárez 2000; ANDES-E, CRBP
*Eaclestyrannus* Draudt, 1930		An, By, Ch, Vl	TL; BC-RBP 12146	Amarillo-Suárez 2000 as *E.masoni*; CRBP
*Eaclesviolaceaviolacea* Lemaire, 1975		Am, Cc		Muñoz and Amarillo-Suárez 2010 as *E.ormondei*; CRBP
*Eaclesviolaceaviocolombiana* Brechlin, 2022	§	By, Cu, St	TL; BC-RBP 8357	Brechlin 2022i; CRBP
Genus *Schausiella* Bouvier, 1930				
*Schausielladenhezorum* Lemaire, 1969		Vl	TL	Lemaire 1969, 1988b; Amarillo-Suárez 2000
*Schausiellajanosi* Brechlin & Käch, 2017		Am, Pu	BC-RBP 12313	CRBP
*Schausiellamoinieri* Lemaire, 1969		Ch	BC-Dec0456	Amarillo-Suárez 2000; CRBP
*Schausiellasinjaevi* Brechlin, 2017		Ca, Cc, Cu	BC-RBP 10986	Brechlin 2017e; CRBP
*Schausiellasubochreata* (Schaus, 1904)		Ca, Ch, Me	BC-Dec1565	Amarillo-Suárez 2000
*Schausiellatatama*﻿ Brechlin, 2017	+	Qu, Ri, Vl	TL; BC-RBP 9334	Brechlin 2017e; CRBP
*Schausiellatoulgoeti* Lemaire, 1969	+	Ch, Vl	TL; BC-RBP 12254	Lemaire 1969; Amarillo-Suárez 2000; Prada-Lara et al. 2019; CRBP
Tribe Citheroniini Harris, 1841				
Genus *Citheronia* Hübner, 1819 [1816]				
*Citheroniaaroa* Schaus, 1896		Ca		Racheli and Vinciguerra 2005; CRBP
*Citheroniabellavista*﻿ Draudt, 1930		An, By, Ch, Ma, St, Vl	TL; BC-RBP 11421	Draudt 1929; Lemaire 1988b; Amarillo-Suárez 2000; Decaëns et al. 2003b; CRBP
*Citheroniacaucensis* Brechlin, 2019	+	Vl	TL; BC-RBP 10691	Brechlin et al. 2019a; CRBP
*Citheroniaequatorialis* Bouvier, 1927		Na, Vl?		Lemaire 1988b; Amarillo-Suárez 2000; CRBP
*Citheroniakaechi* Brechlin, 2019		By, Cc, Cu, Hu, Me, Pu	BC-RBP 9139	Brechlin et al. 2019a; CRBP
*Citheronialaguajira* Brechlin, Meister & van Schayck, 2019		An, Cu, Gj, Hu, Ma, St, To	TL; BC-RBP 9185	Lemaire 1988b and Amarillo-Suárez 2000 as *C.lobesis*; Brechlin et al. 2019a; CRBP
*Citheronialaocandensis* Brechlin, Meister & van Schayck, 2019		Me	BC-Dec0214	Racheli and Racheli 2006 as *C.laocoon*; Brechlin et al. 2019a; CRBP
*Citheroniaphoandensis* Brechlin, 2019		Am, Ca, Me	BC-Dec0278	Amarillo-Suárez 2000 and Racheli and Vinciguerra 2005 as *C.phoronea*; Brechlin et al. 2019a; CRBP
*Citheroniaphochocoensis* Brechlin, 2019		An, Ch, Vl	TL; BC-RBP 5413	Amarillo-Suárez 2000 and Decaëns et al. 2003b as *C.phoronea*; Brechlin et al. 2019a; ANDES-E, CRBP
*Citheroniawinbrechlini* Brechlin, 2019	+	By	TL; BC-RBP 8349	Brechlin et al. 2019a; CRBP
*Citheroniawitti* Brechlin, 2019		Ca, Cn, Pu	BC-Dec1465	Racheli and Vinciguerra 2005 as *C.hamifera*; Brechlin et al. 2019a; CRBP
Genus *Citheronioides* Lemaire, 1988				
*Citheronioidescollaris* (Rothschild, 1907)		By, Ch, Na, Ri, Vl	BC-RBP 12520	Amarillo-Suárez 2000; Decaëns et al. 2007; ANDES-E, CRBP
*Citheronioidessamana* Brechlin, 2022	+	By, Cl	TL; BC-RBP 12402	Brechlin 2022c; CRBP
Genus *Procitheronia* Michener, 1949				
*Procitheroniafenestrata* (Rothschild, 1907)		By, Ca, Cn, Me	BC-Dec1464	Amarillo-Suárez 2000; Racheli and Vinciguerra 2005; ANDES-E, CRBP
Tribe Dryocampini Grote & Robinson, 1866				
Genus *Adeloneivaia* Travassos, 1940				
*Adeloneivaiaacuta* (Schaus, 1896)		By, Ma, Me, St	BC-RBP 11254	Amarillo-Suárez 2000; Decaëns et al. 2007; CRBP
*Adeloneivaiaantkozlovi* Brechlin, 2019		An, Ch	BC-RBP 11466	Brechlin 2019d; CRBP
*Adeloneivaiaboisduvalii* (Doûmet, 1859)		An, By, Ca, Ch, Me, Pu, Vl	BC-RBP 11801	Lemaire 1988b; Amarillo-Suárez 2000; Decaëns et al. 2003b; Racheli and Vinciguerra 2005; Prada-Lara et al. 2019; CRBP
*Adeloneivaiacatobezverkhovi* Brechlin, 2020		By, Cu, Me, Pu	BC-RBP 11567	Brechlin 2020i; CRBP
*Adeloneivaiacatoxantha* (Rothschild, 1907)		Ca, Cc	BC-RBP 11449	Racheli and Vinciguerra 2005; CRBP
*Adeloneivaiacentrojason* Brechlin, 2017		Ch, Vl	BC-RBP 10688	Brechlin 2017b; CRBP
*Adeloneivaiaguajira* Brechlin, 2017	+	Gj	TL; BC-RBP 8661	Brechlin 2017l; CRBP
*Adeloneivaiajacolombiana* Brechlin, 2019	+	An, By, Ce, Cu, Ma	TL; BC-RBP 10229	Brechlin 2019d; CRBP
*Adeloneivaiajametensis* Brechlin, 2019	+	Me	TL; BC-RBP 10670	Brechlin 2019d; CRBP
*Adeloneivaiajaustralica* Brechlin & Meister, 2011		Am, Cc	BC-RBP 12194	Brechlin and Meister 2011c; CRBP
*Adeloneivaiaorientoandensis* Brechlin & Meister, 2011		Am, Pu	BC-RBP 12369	Brechlin and Meister 2011c; CRBP
*Adeloneivaiapallid*a Lemaire, 1982		An, By, Ca, Cc, Cl, Me	BC-RBP 11464	Lemaire 1988b, Amarillo-Suárez 2000, and Racheli and Vinciguerra 2005 as *A.subangulata*; Brechlin and Meister 2011c; ANDES-E, CRBP
*Adeloneivaiasantamartaiana* Brechlin, 2017	+	Ma	TL; BC-RBP 10410	Brechlin 2017l; CRBP
Genus *Adelowalkeria* Travassos, 1941				
*Adelowalkeriacaeca* Lemaire, 1969		Ch, Vl	TL	Lemaire 1969; Amarillo-Suárez 2000; Prada-Lara et al. 2019; CRBP
*Adelowalkeriaeugenicolombiana* Brechlin & Meister, 2011	+	Cu, Ma	TL; BC-FMP-0875	Brechlin and Meister 2011c; CRBP
*Adelowalkeriakitchingi* Brechlin & Meister, 2011		Am, Pu		Brechlin and Meister 2011c; CRBP
*Adelowalkeriawinbrechlini* Brechlin, 2017		An, By, Ch, St	TL; BC-RBP 9874	Amarillo-Suárez 2000 as *A.caeca*; Brechlin 2017m; CRBP
*Adelowalkeriawitti* Brechlin & Meister, 2011		Ca, Pu		Racheli and Vinciguerra 2005 as *A.plateada*; CRBP
Genus *Cicia* Oiticica Filho, 1964				
*Ciciapelotandana* Brechlin, 2023		Ca		Racheli and Vinciguerra 2005 as *C.pelota*; Brechlin 2023e
Genus *Citioica* Travassos & Noronha, 1965				
*Citioicaanalis* (Rothschild, 1907)		Am, Pu	BC-RBP 12370	Brechlin 2022b; CRBP
*Citioicacolombiana* Brechlin, 2017	+	An, By, Ch, St	TL; BC-RBP 9225	Amarillo-Suárez 2000, Decaëns et al. 2003b, and 2007 as *C.anthonilis*; Brechlin 2017c; CRBP
*Citioicagrisecolombiana* Brechlin, 2017	+	An, By, Ch	TL; BC-RBP 10110	Brechlin 2017c; CRBP
*Citioicakaechi* Brechlin, 2017		Cu, Me	BC-RBP 12175	Amarillo-Suárez 2000 as *C.homoea*; Brechlin 2017c; CRBP
*Citioicarubrocanescens* Brechlin & Meister, 2011		Ca, Me	BC-RBP 9832	Amarillo-Suárez 2000, Racheli and Vinciguerra 2005, and Jiménez-Bolívar et al. 2021 as *C.anthonilis*; Brechlin and Meister 2011c; CRBP
Genus *Othorene* Boisduval, 1872				
*Othorenecarameridensis* Brechlin & Meister, 2013		An, Ce, Ma	BC-RBP 11184	Brechlin and Meister 2013c; CRBP
*Othorenepurpurascens* (Schaus, 1905)		Ca, Me		Amarillo-Suárez 2000; Racheli and Vinciguerra 2005; ANDES-E, CRBP
*Othorenevanschayckorum* Brechlin & Meister, 2011		Ch, Na, Vl	BC-RBP 10684	Amarillo-Suárez 2000, Decaëns et al. 2003b, and Prada-Lara et al. 2019 as *O.purpurascens*; Brechlin and Meister 2011c; CRBP
*Othorenewinbrechlini* Brechlin & Meister, 2011		Am, Ca, Cc, Pu	IAvH-E-190475	Racheli and Vinciguerra 2005 as *O.hodeva*; Brechlin and Meister 2011j; ANDES-E, CRBP
Genus *Psilopygida* Michener, 1949				
Subgenus Psigida Oiticica Filho, 1959				
Psilopygida (Psigida) apollinairei (Dognin, 1919)		Ca, Cn, Me, Vi	TL; BC-RBP 9621	Dognin 1919; Amarillo-Suárez 2000 as *P. P. walkeri*; ANDES-E, CRBP
Genus *Ptiloscola* Michener, 1949				
*Ptiloscoladescimoni* Lemaire, 1971		Cu	BC-Dec0270	CRBP
*Ptiloscolalilacina* (Schaus, 1900)	+	An, By, Ch, Cu, St, Vl	TL; BC-RBP 8331	Schaus 1900; Amarillo-Suárez 2000; Decaëns et al. 2003b, 2007; ANDES-E, CRBP
*Ptiloscolameta* Brechlin, 2020	+	Cn, Me	TL; BC-RBP 10780	Brechlin 2020c; CRBP
*Ptiloscolasantamartensis* Brechlin, 2017	+	Ce, Ma	TL; BC-RBP 10417	Brechlin 2017d; CRBP
*Ptiloscolawolfei* Brechlin & Meister, 2008		Am, Ca, Pu	BC-RBP 12371	Amarillo-Suárez 2000 and Racheli and Vinciguerra 2005 as *P.photophila*; ANDES-E, CRBP
Genus *Rachesa* Michener, 1949				
*Rachesabreteuilicaucensis* Lemaire, 1969	§	An, Ch, Cl, Qu, Ri, Vl	TL; BC-RBP 10643	Lemaire 1969; Amarillo-Suárez 2000 as *R.breteuili*; ANDES-E, CRBP
*Rachesadianae* Brechlin, 2017	+	St	TL; BC-RBP 8351	Brechlin 2017k; CRBP
*Rachesahuilana* Brechlin, 2019	+	Hu	TL; BC-RBP 11138	Brechlin 2019b; CRBP
*Rachesasvetlanae* Brechlin, 2017	+	By, Cu, Hu, St	TL; BC-RBP 8033	Brechlin 2017k; CRBP
Genus *Syssphin*x Hübner, 1819 [1816]				
*Syssphinxbidmagdaleniana* Brechlin, 2017	+	Ma	TL; BC-RBP 10315	Brechlin 2017a; CRBP
*Syssphinxcentriantioquiana* Brechlin, 2017	+	An, Ch, Cl	TL; BC-RBP 8995	Brechlin 2017a; ANDES-E, CRBP
*Syssphinxcentriboyacensis* Brechlin, 2017	+	By	TL; BC-RBP 8352	Brechlin 2017a; CRBP
*Syssphinxcentrimacula* (Strand, 1912)		Ca, Cc	BC-RBP 11479	CRBP
*Syssphinxchocoensis* Lemaire, 1988		Ch, Ma	TL; BC-RBP 10730	Lemaire 1988b; Amarillo-Suárez 2000; Prada-Lara et al. 2019; CRBP
*Syssphinxcundinamarcana* Brechlin, 2019	+	Cu	TL; BC-RBP 10664	Brechlin 2019a; CRBP
*Syssphinxjasonoides* (Lemaire, 1971)	+	Vl	TL	Lemaire 1971a, 1988b; Amarillo-Suárez 2000
*Syssphinxmolin*a (Cramer, 1780)		An, Ch, Cu, Hu, Na, Vl	BC-Dec0317	Lemaire 1988b; Amarillo-Suárez 2000; Decaëns et al. 2003b; ANDES-E, CRBP
*Syssphinxquadrilineataocclusa* (Dognin, 1916)		An, By, Ce, Ch, Cu, Ma, St, Vl	TL; BC-RBP 9186	Dognin 1916; Lemaire 1988b; Amarillo-Suárez 2000; Decaëns et al. 2003b; ANDES-E, CRBP
*Syssphinxquindana* Brechlin, 2019	+	Qu	TL; BC-RBP 10720	Brechlin 2019a; CRBP
*Syssphinxriekerti* Brechlin & Meister, 2011		Pu		Brechlin and Meister 2011c; CRBP
*Syssphinxsantamartaensis* Brechlin, 2017	+	Ma	TL; BC-RBP 10413	Brechlin 2019a; CRBP
*Syssphinxsmithi* (Druce, 1904)		An, By, Cu, Gj, Hu, Ma, To, Vl	TL; BC-RBP 10733	Druce 1904; Lemaire 1988b; Amarillo-Suárez 2000; CRBP
*Syssphinxtatama* Brechlin, 2017	+	An, Ri	TL; BC-RBP 9564	Brechlin 2017a; ANDES-E, CRBP
*Syssphinxubalana* Brechlin, 2019	+	Cu	TL; BC-RBP 11480	Brechlin 2019a; CRBP
Subfamily Cercophaninae Jordan, 1924				
Tribe Janiodini Jordan, 1924				
Genus *Janiodes* Jordan, 1924				
*Janiodesdogboyacana* Brechlin, 2020	+	By	TL; BC-RBP 9628	Brechlin 2020e; CRBP
*Janiodesdogcaliana* Brechlin, 2023	+	Vl	TL; BC-RBP 12798	Brechlin 2023i; CRBP
*Janiodesdogfranciscona* Brechlin, 2020	+	Cc, Cu, Hu, Na, Pu	TL; BC-RBP 11150	Brechlin 2020e; CRBP
*Janiodesdogfrontino* Brechlin, 2023	+	An	TL; BC-RBP 12356	Brechlin 2023i; CRBP
*Janiodesdogjardina* Brechlin, 2023	+	An	TL; BC-RBP 12333	Brechlin 2023i; CRBP
*Janiodesdoglagruta* Brechlin, 2023	+	Cl, Vl	TL; BC-RBP 12327	Brechlin 2023i; CRBP
*Janiodesdoglalibia* Brechlin, 2020	+	To	TL; BC-RBP 9865	Brechlin 2020e; CRBP
*Janiodesdogletrasa* Brechlin, 2023	+	To	TL; BC-RBP 12357	Brechlin 2023i; CRBP
*Janiodesdognini* Jordan, 1924	+	Qu, Ri	TL; BC-RBP 8037	Jordan 1924; Brechlin 2020e; CRBP
*Janiodesdogpuerres* Brechlin, 2023	+	Na	TL; BC-RBP 12715	Brechlin 2023i; CRBP
*Janiodesdogpurace* Brechlin, 2020	+	Cc, Hu	TL; BC-RBP 11652	Brechlin 2020e; CRBP
*Janiodesdogputumayona* Brechlin, 2020	+	Pu	TL; BC-RBP 10662	Brechlin 2020e; CRBP
*Janiodesdogsonsona* Brechlin, 2023	+	An	TL; BC-RBP 12331	Brechlin 2023i; CRBP
*Janiodesdoguramita* Brechlin, 2023	+	An	TL; BC-RBP 12713	Brechlin 2023i; CRBP
*Janiodesecabriaqui* Brechlin, 2023	+	An	TL; BC-RBP 12702	Brechlin 2023i; CRBP
*Janiodesecarcabuco* Brechlin, 2020	+	By, St	TL; BC-RBP 8268	Brechlin 2020e; CRBP
*Janiodeseccalarca* Brechlin, 2020	+	Qu, To, Vl	TL; BC-RBP 8039	Brechlin 2020e; CRBP
*Janiodeseccarchensis* Brechlin, 2020		Na	BC-RBP 12800	Brechlin 2023i; CRBP
*Janiodeseccolombiana* Brechlin, 2020	+	By, Ca, Cc, Cu, Hu, Na, Pu	TL; BC-RBP 11257	Brechlin 2020e; CRBP
*Janiodeseccumbrana* Brechlin, 2020	+	Cl, Qu, Ri, Vl	TL; BC-RBP 10763	Brechlin 2020e; CRBP
*Janiodesecdelnorte* Brechlin, 2020	+	By	TL; BC-RBP 10772	Brechlin 2020e; CRBP
*Janiodesecfrontino* Brechlin, 2023	+	An	TL; BC-RBP 12334	Brechlin 2023i; CRBP
*Janiodesecinsora* Brechlin, 2023	+	An	TL; BC-RBP 12482	Brechlin 2023i; CRBP
*Janiodeseclagruta* Brechlin, 2023	+	Cl	TL; BC-RBP 12486	Brechlin 2023i; CRBP
*Janiodesecmarmolana* Brechlin, 2020	+	Ca, Cc, Hu	TL; BC-RBP 11700	Brechlin 2020e; CRBP
*Janiodesecminasa* Brechlin, 2020		Na, Pu	BC-RBP 11159	Brechlin 2020e; CRBP
*Janiodesecpenasblancas* Brechlin, 2020	+	By	TL; BC-RBP 9627	Brechlin 2020e; CRBP
*Janiodesecputnarino* Brechlin, 2020	+	Na, Pu	TL; BC-RBP 10727	Brechlin 2020e; CRBP
*Janiodesecsumapasa* Brechlin, 2020	+	Cu	TL; BC-RBP 10303	Brechlin 2020e; CRBP
*Janiodesectatama* Brechlin, 2020	+	An, Ri	TL; BC-RBP 9623	Brechlin 2020e; CRBP
*Janiodesectolima* Brechlin, 2020	+	An, To	TL; BC-RBP 8276	Brechlin 2020e; CRBP
*Janiodesecurrao* Brechlin, 2023	+	An	TL; BC-RBP 12364	Brechlin 2023i; CRBP
*Janiodesecyarumala* Brechlin, 2020	+	An	TL; BC-RBP 9679	Brechlin 2020e; CRBP
*Janiodesguascan*a Brechlin, 2020	+	Cu	TL; BC-RBP 10782	Brechlin 2020e; CRBP
*Janiodeslavbelmirana* Brechlin, 2023	+	An	TL; BC-RBP 12290	Brechlin 2023i; CRBP
*Janiodeslavcabrera* Brechlin, 2020	+	Cu, To	TL; BC-RBP 11682	Brechlin 2020e; CRBP
*Janiodeslavcarchensis* Brechlin, 2020		Na	BC-RBP 12801	Brechlin 2023i; CRBP
*Janiodeslavconcepciona* Brechlin, 2020	+	Cu	TL; BC-RBP 11683	Brechlin 2020e; CRBP
*Janiodeslavfilandia* Brechlin, 2023	+	Qu	TL; BC-RBP 12711	Brechlin 2023i; CRBP
*Janiodeslavgachala* Brechlin, 2020	+	Cu	TL; BC-RBP 11146	Brechlin 2020e; CRBP
*Janiodeslavhollinensis* Brechlin, 2020		Cu, Me	BC-RBP 11147	Brechlin 2020e; CRBP
*Janiodeslavinzana* Brechlin, 2023	+	Cc	TL; BC-RBP 12324	Brechlin 2023i; CRBP
*Janiodeslavirgensis* Brechlin, 2020	+	By	TL; BC-RBP 11680	Brechlin 2020e; CRBP
*Janiodeslavonzaga* Brechlin, 2020	+	By	TL; BC-RBP 9642	Brechlin 2020e; CRBP
*Janiodeslavputumayona* Brechlin, 2020	+	Na, Pu	TL; BC-RBP 11799	Brechlin 2020e; CRBP
*Janiodeslavricaurte* Brechlin, 2023	+	Na	TL; BC-RBP 12804	Brechlin 2023i; CRBP
*Janiodeslavristolima* Brechlin, 2020	+	An, Cl, Ri, To	TL; BC-RBP 9641	Brechlin 2020e, 2023i; CRBP
*Janiodeslavsinjaevi* Brechlin, 2020		Hu	BC-RBP 10973	Brechlin 2020e; CRBP
*Janiodeslavtatama* Brechlin, 2020	+	An, Qu, Ri	TL; BC-RBP 9576	Brechlin 2020e, 2023i; CRBP
*Janiodeslavtogui* Brechlin, 2020	+	By, St	TL; BC-RBP 9643	Brechlin 2020e; CRBP
*Janiodeslavyarumala* Brechlin, 2020	+	An, Cl, Na, Ri	TL; BC-RBP 9675	Brechlin 2020e; CRBP
*Janiodesnaputumayona* Brechlin, 2020	+	Na, Pu	TL; BC-RBP 10663	Brechlin 2020e, 2023i; CRBP
*Janiodespardognini* Brechlin, 2020	+	Cl, Ri	TL; BC-RBP 10152	Brechlin 2020e; CRBP
*Janiodespinzonica* Brechlin, 2020	+	By, Cu	TL; BC-RBP 8290	Brechlin 2020e; CRBP
*Janiodesrusarcabucona* Brechlin, 2020	+	By, St	TL; BC-RBP 9625	Brechlin 2020e; CRBP
*Janiodesrusbogotan*a Brechlin, 2020	+	Cu	TL; BC-RBP 11713	Brechlin 2020e; CRBP
*Janiodesruscalarca* Brechlin, 2020	+	Qu	TL; BC-RBP 8038	Brechlin 2020e; CRBP
*Janiodesruscarchensis* Brechlin, 2020		Na	BC-RBP 12790	Brechlin 2023i; CRBP
*Janiodesrusconcepciona* Brechlin, 2020	+	Cu	TL; BC-RBP 8272	Brechlin 2020e; CRBP
*Janiodesrusflorencia* Brechlin, 2023	+	Ca	TL; BC-RBP 12789	Brechlin 2023i; CRBP
*Janiodesrusfrontino* Brechlin, 2023	+	An	TL; BC-RBP 12338	Brechlin 2023i; CRBP
*Janiodesrusgachala* Brechlin, 2020	+	Cu	TL; BC-RBP 11190	Brechlin 2020e; CRBP
*Janiodesrusguascana* Brechlin, 2020	+	By, Cu	TL; BC-RBP 10765	Brechlin 2020e; CRBP
*Janiodesrusjardina* Brechlin, 2023	+	An, Cl, Na, Vl	TL; BC-RBP 12358	Brechlin 2023i; CRBP
*Janiodesruslagruta* Brechlin, 2023	+	Cl	TL; BC-RBP 12487	Brechlin 2023i; CRBP
*Janiodesrusletrasa* Brechlin, 2023	+	To	TL; BC-RBP 12365	Brechlin 2023i; CRBP
*Janiodesrusmarmolana* Brechlin, 2020	+	Ca, Hu	TL; BC-RBP 11698	Brechlin 2020e; CRBP
*Janiodesrusminasa* Brechlin, 2020		Cc, Na, Pu	TL; BC-RBP 11153	Brechlin 2020e; CRBP
*Janiodesrusnortana* Brechlin, 2020	+	NS, St	TL; BC-RBP 10556	Brechlin 2020e; CRBP
*Janiodesrusperijana* Brechlin, 2023	+	Ce	TL; BC-RBP 12794	Brechlin 2023i; CRBP
*Janiodesruspuerres* Brechlin, 2023	+	Na	TL; BC-RBP 12688	Brechlin 2023i; CRBP
*Janiodesrusputhuilana* Brechlin, 2020	+	Hu, Na, Pu	TL; BC-RBP 11156	Brechlin 2020e; CRBP
*Janiodesrusputumayona* Brechlin, 2020	+	Pu	TL; BC-RBP 11157	Brechlin 2020e; CRBP
*Janiodesrusrondona* Brechlin, 2020	+	By	TL; BC-RBP 10767	Brechlin 2020e; CRBP
*Janiodesrussangayana* Brechlin, 2020		Na	BC-RBP 12690	Brechlin 2020e; CRBP
*Janiodesrustogui* Brechlin, 2020	+	By	TL; BC-RBP 10914	Brechlin 2020e; CRBP
*Janiodesrustolim*a Brechlin, 2020	+	An, Cl, Ri, To	TL; BC-RBP 8279	Brechlin 2020e; CRBP
*Janiodesrustunjana* Brechlin, 2020	+	By	TL; BC-RBP 8287	Brechlin 2020e; CRBP
*Janiodesrusyarumala* Brechlin, 2023	+	An	TL; BC-RBP 12367	Brechlin 2023i; CRBP
*Janiodessumapasa* Brechlin, 2020	+	Cu	TL; BC-RBP 10222	Brechlin 2020e; CRBP
*Janiodesvirgata* Jordan, 1924	+	Cl, Qu, To	TL; BC-RBP 11260	Jordan 1924; Brechlin 2020e; CRBP
Subfamily Hemileucinae Grote & Robinson, 1866				
Tribe Hemileucini Grote & Robinson, 1866				
Subtribe Automeriina Bouvier, 1928				
Genus *Automerina* Michener, 1949				
*Automerinaaucametana* Brechlin & Comoglio, 2023	+	Ca, Cn, Me	TL; BC-RBP 12752	Brechlin and Comoglio 2023c; CRBP
*Automerinaauguajira* Brechlin, 2018	+	Cu, Gj, Ma	TL; BC-RBP 10568	Brechlin 2018a; CRBP
*Automerinaauletes* (Herrich-Schäffer, 1854)		Ca, Cn	BC-Dec1470	Racheli and Vinciguerra 2005; Jiménez-Bolívar et al. 2021; CRBP
*Automerinacaudatula* (C. Felder & R. Felder, 1874)		Am	IAvH-E-190372	CRBP
*Automerinaesmeraletes* Brechlin, Käch & Meister, 2013		Ch, Vl	BC-RBP 10714	Brechlin et al. 2013a; CRBP
*Automerinayungasletes* Brechlin & Meister, 2011		Am, Cc, Pu	BC-RBP 11231	Brechlin and Meister 2011i; CRBP
Genus *Automeris* Hübner, 1819 [1816]				
*Automerisabdancaldasa* Brechlin, 2023	+	An, Cl	TL; BC-RBP 12585	Brechlin 2023c; CRBP
*Automerisabdanrivalle* Brechlin, 2023	+	An, Qu, Ri, Vl	TL; BC-RBP 10645	Brechlin 2023c; CRBP
*Automerisabdantolima* Brechlin, 2023	+	An, Ca, Hu, To	TL; BC-RBP 8022	Brechlin 2023c; CRBP
*Automerisabdgachala* Brechlin, 2023	+	Cu, St	TL; BC-RBP 12583	Brechlin 2023c; CRBP
*Automerisabdominali*s (C. Felder & R. Felder, 1874)	+	Cu?	TL	Felder and Felder 1874; Amarillo-Suárez 2000; Lemaire 2002; Brechlin 2023c; ANDES-E, CRBP
*Automerisabdomimeridensis* Brechlin & Meister, 2011		Ce	BC-RBP 12759	Brechlin 2023c; CRBP
*Automerisabdominapoensis* Brechlin & Meister, 2011		Cc, Pu	BC-RBP 12579	Brechlin and Meister 2011f; CRBP
*Automerisabdomipichinchensis* Brechlin & Meister, 2011		Na	BC-RBP 12787	Brechlin and Meister 2011f; CRBP
*Automerisabdsanboyacensis* Brechlin, 2023	+	By, St	TL; BC-RBP 9248	Brechlin 2023c; CRBP
*Automerisalticarchensis* Brechlin, Käch & Meister, 2013		Na	BC-RBP 12621	Brechlin 2023d; CRBP
*Automerisalticola* Lemaire, 1975		Vl	BC-RBP 12620	Brechlin 2023d; CRBP
*Automerisamageus* Brechlin, 2021		Am, Cn	IAvH-E-190251	Brechlin 2021i; Jiménez-Bolívar et al. 2021
*Automerisamaloretensis* Brechlin & Meister, 2011		Am, By, Cn	BC-RBP 8669	Brechlin and Meister 2011f; CRBP
*Automerisandensis* Brechlin & Käch, 2017		Cn	RR-COL2015-121	Brechlin et al. 2017
*Automerisangulatus* Conte, 1906		By, Ca, Cu, Hu, Me	BC-Dec0646	Amarillo-Suárez 2000 and Decaëns et al. 2007 as *A.hamata*; ANDES-E, CRBP
*Automerisargentiferaargentifer*a Lemaire, 1966		An, Ch, Na, Ri, To, Vl	TL; BC-RBP 3546	Lemaire 1966b, 2002; Amarillo-Suárez 2000 as *A.banus*; Decaëns et al. 2003b as *A.banusargentifera*; ANDES-E, CRBP
*Automerisargentiferaargorientalis* Brechlin, 2023	§	An, By, Cu, Ri, St, To, Vl	TL; BC-RBP 12598	Decaëns et al. 2007 as *A.banusproxima*; Brechlin 2023c; CRBP
*Automerisatrolimbata* Lemaire, 1973		Cc, Pu	BC-RBP 12351	CRBP
*Automerisbarbosan*a Brechlin, 2021	+	An, By, St	TL; BC-RBP 11747	Brechlin 2021i; CRBP
*Automerisbilinea* (Walker, 1855)		Cn	IAvH-E-190279	Jiménez-Bolívar et al. 2021
*Automerisboops* (C. Felder & R. Felder, 1874)		Me	BC-RBP 10835	CRBP
*Automeriscaucensis* Lemaire, 1976		Vl	TL	Lemaire 1976a, 2002; CRBP
*Automerischoco* Brechlin & Meister, 2011	+	Ch, Ri, Vl	TL; BC-FMP-0501	Decaëns et al. 2003b as *A.celata*; Brechlin and Meister 2011f; CRBP
*Automeriscinctistrig*a (C. Felder & R. Felder, 1874)		Am, Ca, Cu?, Gn, Me	TL; BC-Dec0713	Felder and Felder 1874; Amarillo-Suárez 2000; Decaëns et al. 2021; ANDES-E, CRBP
*Automeriscomoglioi* Brechlin, 2023	+	Cu	TL; BC-RBP 12616	Brechlin 2023d; CRBP
*Automerisconceptiona* Brechlin, 2016	+	Cu	TL; BC-RBP 8271	Brechlin 2016c; CRBP
*Automeriscryptica* Dognin, 1911		Na, Vl	TL; BC-RBP 10610	Dognin 1911a; Amarillo-Suárez 2000; CRBP
*Automeriscundinamarcensi*s Brechlin & Meister, 2011		An, By, Cu, St	TL; BC-RBP 3656	Brechlin and Meister 2011f; ANDES-E, CRBP
*Automeriscurvilinea* Schaus, 1906		Am		Amarillo-Suárez 2000; ANDES-E, CRBP
*Automeriscuscosylviae* Brechlin & Meister, 2011		Cc	BC-RBP 12580	Brechlin and Meister 2011f; CRBP
*Automerisdagmara*e Brechlin & Meister, 2011		An, By, Cu, Ma, To, Vl	BC-RBP 3524	Amarillo-Suárez 2000, Lemaire 2002, and Decaëns et al. 2007 as *A.metzli*; Brechlin and Meister 2011f; ANDES-E, CRBP
*Automerisdenticulata* Conte, 1906		Me	BC-Dec0694	CRBP
*Automerisdognini* Lemaire, 1967	+	Cn, Me	TL; BC-FMP-0571	Lemaire 1966a, 2002; Amarillo-Suárez 2000; CRBP
*Automerisduchartrei* Bouvier, 1936	+	Cl, Qu, To, Vl	TL; BC-RBP 8343	Bouvier 1936; Amarillo-Suárez 2000; Lemaire 2002; CRBP
*Automerisecuata* Brechlin & Meister, 2011		Pu		Brechlin and Meister 2011f; CRBP
*Automerisexigua* Lemaire, 1977		An, Ch, Na, Vl	TL; BC-RBP 3523	Lemaire 1977, 2002; Amarillo-Suárez 2000; Decaëns et al. 2003b; Prada-Lara et al. 2019; CRBP
*Automerisfabiani* Brechlin & Meister, 2011		Ca, Cn, Me, Pu	BC-RBP 12589	Brechlin and Meister 2011f; Jiménez-Bolívar et al. 2021 as *A.moresca*; ANDES-E, CRBP
*Automerisfieldifieldi* Lemaire, 1969		Ch, Ri, Vl	TL; BC-MNHN0240	Lemaire 1969; Amarillo-Suárez 2000; Decaëns et al. 2003b; ANDES-E, CRBP
*Automerisfieldifieldseptentrides* Brechlin, 2017		An, By, Cu, St	TL; BC-RBP 10402	Brechlin et al. 2017; ANDES-E, CRBP
*Automerisfrontino* Brechlin, 2022	+	An	TL; BC-RBP 12248	Brechlin 2022m; CRBP
*Automerisgadouae* Lemaire, 1966		By, Ca, Me, Vl	BC-RBP 8397	CRBP
*Automerisgunneri* Brechlin, 2016	+	An, Cl, Qu, To	TL; BC-RBP 9864	Brechlin 2016c; CRBP
*Automerishamata* Schaus, 1906		Ch, Gj, Ma, Vl	BC-RBP 9046	Amarillo-Suárez 2000; Lemaire 2002; Decaëns et al. 2003b; ANDES-E, CRBP
*Automerishandschugi* Brechlin, 2017	+	By, St	TL; BC-RBP 8398	Decaëns et al. 2007 as *A.duchartrei*; Brechlin et al. 2017; CRBP
*Automerisharriamazonica* Brechlin & Meister, 2011		Hu	BC-RBP 11131	Brechlin and Meister 2011f; CRBP
*Automerishausmanni* Brechlin, 2016	+	By	TL; BC-RBP 9676	Brechlin 2016c; CRBP
*Automerisiguaquensis* Lemaire & Amarillo, 1992	+	By, Cu, St	TL	Lemaire and Amarillo-Suárez 1992; Amarillo-Suárez 2000; Lemaire 2002; CRBP
*Automerisincarnata* (Walker, 1865)		An, By, Cu, Gj, Hu, Ma, Me, To	TL; BC-RBP 10567	Walker 1865; Amarillo-Suárez 2000; Lemaire 2002; Decaëns et al. 2007; CRBP
*Automerisinnoxia* Schaus, 1906		Cn	BC-Dec1065	CRBP
*Automerisisabellae* Brechlin & Käch, 2017		Na	BC-RBP 12760	CRBP
*Automerisisnosa* Brechlin, 2022	+	Cc, Hu	TL; BC-RBP 12244	Brechlin 2022m; CRBP
*Automerisiwanowitschi* Brechlin, Käch & Meister, 2013		An, Cc, Hu, To	BC-RBP 8273	Brechlin et al. 2013b; CRBP
*Automerisjanrudloffi* Brechlin & Meister, 2011	+	An, Cu, Ri, To	TL; BC-RBP 3660	Brechlin and Meister 2011f; CRBP
*Automerisjanus* (Cramer, 1775)		Am	IavH-E-190356	CRBP
*Automerisjucunda* (Cramer, 1779)		By, Ca, Ch, Cn, Cu, Gn, Gv, Me, To, Vl	BC-RBP 3579	Amarillo-Suárez 2000; Lemaire 2002; Decaëns et al. 2003b, 2007; Comoglio and Racheli 2016; ANDES-E, CRBP
*Automeriskaechi* Brechlin & Meister, 2011		Pu		Brechlin and Meister 2011f; CRBP
*Automerisliberia* (Cramer, 1780)		Am, Ca, Cu, Gv, Me	BC-Dec1655	Amarillo-Suárez 2000; Lemaire 2002; ANDES-E, CRBP
*Automerisllaneros* Decaëns, Rougerie & Bonilla, 2021	+	Cn, Me	TL; BC-Dec0711	Decaëns et al. 2021; ANDES-E, CRBP
*Automerismagdaleniana* Brechlin & Meister, 2011	+	An, Cu?, To	TL; BC-RBP 3657	Brechlin and Meister 2011f; ANDES-E, CRBP
*Automerismaximae* Brechlin & Witt, 2017	+	By	TL; BC-RBP 9994	Brechlin and Witt 2017; CRBP
*Automerismidenapoensis* Brechlin & Meister, 2011		Am, Ca, Pu	BC-RBP 12508	Brechlin and Meister 2011f; CRBP
*Automerismineros* Decaëns, Rougerie & Bonilla, 2021	+	By	TL; BC-Dec0551	Decaëns et al. 2007 as *A.midea*; Decaëns et al. 2021
*Automerismixtus* Bouvier, 1936		Am, Hu, Pu	IAvH-E-190358	Jiménez-Bolívar et al. 2021 as *A.larra*; ANDES-E, CRBP
*Automerisniepelti* Draudt, 1929		Cc, Ch, Na, Vl	TL; BC-Dec1043	Draudt 1929; Amarillo-Suárez 2000; Lemaire 2002; CRBP
*Automerisoccidentorestes* Brechlin & Meister, 2011		Ca, Gv, Pu		Brechlin and Meister 2011f; ANDES-E, CRBP
*Automerisoiticicai* Lemaire, 1966	+	An, Cc?, Vl	TL; BC-RBP 10577	Lemaire 1966b, 2002; Amarillo-Suárez 2000; CRBP
*Automerisparafera* Brechlin & Meister, 2014		Pu		Brechlin and Meister 2014c; CRBP
*Automerisparapichinchensis* Brechlin & Meister, 2011		An, Ch, Vl	BC-RBP 10612	Amarillo-Suárez 2000, Decaëns et al. 2003b, 2007, and Prada-Lara et al. 2019 as *A.zugana*; Brechlin and Meister 2011f; ANDES-E, CRBP
*Automerispastaziana* Brechlin & Meister, 2011		Me, Pu	BC-RBP 8400	Brechlin and Meister 2011f; CRBP
*Automerispeggyanaepeggyanae* Brechlin, 2016	+	By, St	TL; BC-RBP 9867	Brechlin 2016c; CRBP
*Automerispeggyanaepegbogotana* Brechlin, 2016	§	By, Cu	TL; BC-RBP 10101	Brechlin 2016c; CRBP
*Automerisphrynon* Druce, 1897		Ch, Vl		Lemaire 2002; CRBP
*Automerispinasiana* Brechlin & Meister, 2014		Ch	BC-FMP-1995	Brechlin and Meister 2014c; CRBP
*Automerispostalbida* Schaus, 1900		Ch, Na, Ri, Vl		Amarillo-Suárez 2000; Lemaire 2002; Prada-Lara et al. 2019; ANDES-E, CRBP
*Automerispraemargaritae* Lemaire, 2002		By		Lemaire 2002; CRBP
*Automerisputumayona* Brechlin, 2020	+	Pu	TL; BC-RBP 11181	Brechlin and Meister 2020; CRBP
*Automerisrisquindensis* Brechlin, 2016	+	Cl, Ri, Qu	TL; BC-RBP 9323	Brechlin 2016c; CRBP
*Automerisrudloffjani* Brechlin & Meister, 2011	+	An, Cu, To	TL; BC-RBP 6105	Brechlin and Meister 2011f; CRBP
*Automerisschwartzi* Lemaire, 1967		Am, Ca, Pu	TL	Lemaire 1966a, 2002; Amarillo-Suárez 2000; Racheli and Vinciguerra 2005; CRBP
*Automerisserpina* Butler, 1878		Am	IAvH-E-190391	Jiménez-Bolívar et al. 2021 as *A.occidentorestes*
*Automerissubobscurasubobscura* Weymer, 1909	§	By, Cu, St	TL; BC-RBP 3547	Weymer 1909; Amarillo-Suárez 2000 as *A.amanda*; Lemaire 2002; Brechlin 2023c; ANDES-E, CRBP
*Automerissubobscuradenhezorum* Lemaire, 1966	§	An?, Vl	TL; BC-MNHN0644	Lemaire 1966b and Amarillo-Suárez 2000 as *A.denhezorum*; Brechlin 2023c; ANDES-E, CRBP
*Automerissubobscuralichyi* Lemaire, 1966		Cc, Ce, Hu, Me, NS, St	BC-RBP 12591	Brechlin 2023c; CRBP
*Automerissubobscuralimpida* Lemaire, 1966		Pu		Brechlin 2023c; CRBP
*Automeristamsi* Lemaire, 1966		Ma	TL; BC-RBP 11132	Lemaire 1966b, 2002; CRBP
*Automeristolimaiensis* Brechlin & Meister, 2011	+	An, Cu, To	TL; BC-RBP 3654	Brechlin and Meister 2011f; CRBP
*Automerisvanschaycki* Brechlin & Meister, 2011		Ch	BC-Dec1051	Brechlin and Meister 2011f; CRBP
*Automerisvincentensis* Brechlin, 2017	+	Ri	TL; BC-RBP 9579	Brechlin et al. 2017; CRBP
*Automerisvomona* Schaus, 1906		An, By, Cu, Na, St, To	BC-RBP 11662	Amarillo-Suárez 2000; Lemaire 2002; Decaëns et al. 2007; ANDES-E, CRBP
*Automerisyarumala* Brechlin, 2021	+	An	TL; BC-RBP 10834	Brechlin 2021i; CRBP
*Automeriszaruma* Schaus, 1898		An, By, Ch, Na, Ri, Vl	BC-RBP 3533	Amarillo-Suárez 2000 and Decaëns et al. 2003b as *A.beltizaruma*; Prada-Lara et al. 2019 as *A.belti*; ANDES-E, CRBP
*Automeriszurouae* Brechlin & Meister, 2011		An, By, Ce, Cu, Hu, Ma, To	TL; BC-RBP 3545	Brechlin and Meister 2011f; CRBP
Genus *Catacantha* Bouvier, 1930				
*Catacanthaecorientalis* Brechlin, Käch & Meister, 2013		Ca, Cc, Pu	BC-RBP 11767	Brechlin et al. 2013g; CRBP
*Catacanthameta* Brechlin, 2020	+	By, Cu, Me	TL; BC-RBP 8504	Brechlin 2020a; ANDES-E, CRBP
Genus *Erythromeris* Lemaire, 1969				
*Erythromerischristbrechlinaechristbrechlinae* Brechlin, 2016	+	By, Cu, St	TL; BC-RBP 8244	Lemaire 2002 as *E.flexilineata*; Brechlin 2016b; ANDES-E, CRBP
*Erythromerischristbrechlinaepuracana* Brechlin, 2021	§	Cc, Hu	TL; BC-RBP 11653	Brechlin 2021n; CRBP
*Erythromerisflexilineata* (Dognin, 1911)	+	Cl?, Qu, To, Vl	TL; BC-RBP 8260	Dognin 1911b; Amarillo-Suárez 2000; Lemaire 2002; CRBP
*Erythromerisobscurior* Lemaire, 1975		Na, Pu	BC-RBP 11774	CRBP
*Erythromerissaturniata* (Walker, 1865)	+	By, Cl, Cu, Me, Ri, St	TL; BC-RBP 10563	Walker 1865; Amarillo-Suárez 2000; Lemaire 2002; ANDES-E, CRBP
*Erythromerissonsona* Brechlin, 2021	+	An	TL	Brechlin 2021n; CRBP
Genus *Gamelia* Hübner, 1819 [1816]				
*Gameliaabboyacensis* Brechlin, 2018	+	By, Cu	TL; BC-RBP 10605	Brechlin 2018k; CRBP
*Gameliaaltoflorencia* Brechlin & Comoglio, 2023	+	Ca	TL; BC-RBP 12650	Brechlin and Comoglio 2023a; CRBP
*Gameliabarbacoasa* Brechlin & Comoglio, 2023	+	Na	TL; BC-RBP 12634	Brechlin and Comoglio 2023a; CRBP
*Gameliacabrera* Brechlin, 2018	+	Cu	TL; BC-RBP 10466	Brechlin 2018k; CRBP
*Gameliacaucensis* Brechlin, 2018	+	Vl	TL; BC-RBP 10611	Brechlin 2018k; CRBP
*Gameliacimarrones* Decaëns, Bonilla & Ramirez, 2005	+	Ch, Vl	TL; BC-Dec0544	Decaëns et al. 2003b as *G.abasia*; Decaëns et al. 2005; CRBP
*Gameliacundboyacensis* Brechlin, 2018	+	By, Cu	TL; BC-RBP 8412	Amarillo-Suárez 2000 as *G.neidhoeferi*; Brechlin 2018k; CRBP
*Gameliadenhezi* Lemaire, 1967		Vl	TL	Lemaire 1966c, 2002; Amarillo-Suárez 2000; CRBP
*Gameliaflorencia* Brechlin & Comoglio, 2023	+	Ca	TL; BC-RBP 12806	Brechlin and Comoglio 2023a; CRBP
*Gameliafrontino* Brechlin & Comoglio, 2023	+	An	TL; BC-RBP 12249	Brechlin and Comoglio 2023a; CRBP
*Gameliagordasa* Brechlin & Comoglio, 2023	+	An	TL; BC-RBP 12462	Brechlin and Comoglio 2023a; CRBP
*Gameliahollinensis* Brechlin, Käch & Meister, 2012		Me	BC-RBP 8413	Brechlin and Meister 2012b; CRBP
*Gameliakaechi* Brechlin & Meister, 2012		Na	BC-RBP 12727	Brechlin and Comoglio 2023a; CRBP
*Gameliakiefferi* Lemaire, 1967	+	Cc?, Vl	TL	Lemaire 1966c, 2002; Amarillo-Suárez 2000
*Gamelialacelia* Brechlin, 2018	+	Ri, Vl	TL; BC-RBP 9092	Brechlin 2018k; CRBP
*Gamelialallanada* Brechlin & Comoglio, 2023	+	Na, Vl	TL; BC-RBP 12807	Brechlin and Comoglio 2023a; CRBP
*Gamelialamilagrosa* Brechlin, 2018	+	Ma	TL; BC-RBP 8414	Brechlin 2018k; CRBP
*Gameliamarquezae* Brechlin, 2018	+	By, St	TL; BC-RBP 8250	Brechlin 2018k; CRBP
*Gameliamarmolana* Brechlin, 2020	+	Cc, Hu	TL; BC-RBP 11177	Brechlin 2020f; CRBP
*Gameliaotanchana* Brechlin, 2021	+	By	TL; BC-RBP 11798	Brechlin 2021d; CRBP
*Gameliaparamartiniana* Brechlin & Meister, 2012		Ca, Cc, Cu, Me	BC-RBP 10604	Brechlin and Meister 2012b; ANDES-E, CRBP
*Gameliaparyarumala* Brechlin, 2018	+	An, Qu	TL; BC-RBP 9172	Brechlin 2018k; CRBP
*Gameliapuracana* Brechlin, 2020	+	Hu	TL; BC-RBP 11265	Brechlin 2020f; CRBP
*Gameliaputhuilana* Brechlin, 2020	+	Cc, Hu, Pu	TL; BC-RBP 10976	Brechlin 2020f; ANDES-E, CRBP
*Gameliapyrrhomelas* (Walker, 1855)	+	Cu	TL	Walker 1855b; Amarillo-Suárez 2000; Lemaire 2002
*Gameliaristolima* Brechlin, 2018	+	An, Cl, Ri, To	TL; BC-RBP 8020	Amarillo-Suárez 2000 as *G.neidhoeferi*; Brechlin 2018k; CRBP
*Gameliarubriluna* (Walker, 1862)		By, Cc, Cn, Me, Pu	BC-RBP 6182	CRBP
*Gameliarudloffi* Brechlin & Meister, 2012		An, Cu, Ma, St	BC-RBP 4059	Brechlin and Meister 2012b; CRBP
*Gameliarudloffiana* Brechlin, 2018		Ch		CRBP
*Gameliasalerona* Brechlin, 2020	+	Ch	TL; BC-RBP 11316	Brechlin 2020f; CRBP
*Gameliasamana* Brechlin & Comoglio, 2023	+	Cl	TL; BC-RBP 12406	Brechlin and Comoglio 2023a; CRBP
*Gameliasantboyacensis* Brechlin, 2018	+	By, St	TL; BC-RBP 10661	Brechlin 2018k; CRBP
*Gameliatamarae* Brechlin & Meister, 2012		Ce, St	BC-RBP 10631	Brechlin and Meister 2012b; CRBP
*Gameliatamesisa* Brechlin & Comoglio, 2023	+	An, Ri	TL; BC-RBP 12729	Brechlin and Comoglio 2023a; CRBP
*Gameliatatama* Brechlin, 2018	+	An, Ri	TL; BC-RBP 10465	Brechlin 2018k; ANDES-E, CRBP
*Gameliatatamica* Brechlin, 2018	+	Ri, Vl	TL; BC-RBP 9569	Brechlin 2018k; ANDES-E, CRBP
*Gameliawinbrechlini* Brechlin, 2018	+	Ce, Ma	TL; BC-RBP 10210	Brechlin 2018k; CRBP
*Gameliayarumala* Brechlin, 2018	+	An, Cl	TL; BC-RBP 9682	Brechlin 2018k; CRBP
Genus *Gamelioides* Lemaire, 1988				
*Gamelioideschrisbrechlinae* Brechlin, 2016	+	Qu, To	TL; BC-RBP 8006	Brechlin 2016e; CRBP
*Gamelioidesmachadoi* Brechlin, 2018	+	Cl	TL; BC-RBP 10648	Brechlin 2018d; CRBP
*Gamelioidespeggyae* Brechlin, 2018	+	NS	TL; BC-RBP 10748	Brechlin 2018e; CRBP
*Gamelioidespinzonica* Brechlin, 2016	+	By, Cu	TL; BC-RBP 8263	Brechlin 2016e: CRBP
*Gamelioidessachai* Brechlin, Käch & Meister, 2011		Na	BC-RBP 12512	Brechlin et al. 2011a; CRBP
*Gamelioidessinjaevi* Brechlin, 2016	+	To	TL; BC-RBP 8261	Brechlin 2016e; CRBP
*Gamelioidessochensis* Brechlin, 2018	+	Cu	TL; BC-RBP 11169	Brechlin 2018n; CRBP
*Gamelioideswinbrechlini* Brechlin, 2016	+	By, Cu	TL; BC-RBP 8270	Brechlin 2016e; CRBP
Genus *Hylesia* Hübner, 1820				
Subgenus Darylesia Brechlin, 2022	+			
Hylesia (Darylesia) darjardina Brechlin, 2022	+	An	TL; BC-RBP 12278	Brechlin 2022h; CRBP
Hylesia (Darylesia) daryae Decaëns, Bonilla & Wolfe, 2003	+	By	TL; BC-RBP 10086	Decaëns et al. 2003a; CRBP
Subgenus Hylesia Hübner, 1820				
Hylesia (Hylesia) aencocornex Brechlin & Meister, 2016	+	An, By, St	TL; BC-RBP 9342	Decaëns et al. 2007 as *H.aeneides*; Brechlin et al. 2016a; ANDES-E, CRBP
Hylesia (Hylesia) aeneides aenocciecuadorex Brechlin & Käch, 2016		Na, Vl	BC-RBP 7703	Amarillo-Suárez 2000 and Lemaire 2002 as *H.aeneides*; Brechlin et al. 2016a; ANDES-E, CRBP
Hylesia (Hylesia) amaloretex Brechlin, Meister & van Schayck, 2016		Am	IAvH-E-190366	Brechlin et al. 2016a; Jiménez-Bolívar et al. 2021; CRBP
Hylesia (Hylesia) anchises Lemaire, 1988	+	Vl	TL	Lemaire 1988a, 2002; Amarillo-Suárez 2000
Hylesia (Hylesia) andcaucex andcaucex Brechlin & Meister, 2016	+	Qu, Vl	TL; BC-RBP 8764	Brechlin et al. 2016a; CRBP
Hylesia (Hylesia) andcaucex andentioquiex Brechlin & Meister, 2016	§	An	TL; BC-RBP 8977	Brechlin et al. 2016a; CRBP
Hylesia (Hylesia) andecuadorex Brechlin & Käch, 2016		By, Hu	BC-RBP 9879	Brechlin et al. 2016a; CRBP
Hylesia (Hylesia) andmeridex Brechlin & Meister, 2016		St	BC-RBP 10715	Brechlin et al. 2016a; CRBP
Hylesia (Hylesia) angmetex Brechlin & Meister, 2016	+	Me	TL; BC-RBP 9803	Brechlin et al. 2016a; CRBP
Hylesia (Hylesia) annulata Schaus, 1911		An, Ch, Me, St, Vl	BC-RBP 9043	Lemaire 2002; CRBP
Hylesia (Hylesia) antioquiex Brechlin & Meister, 2016	+	An, St	TL; BC-RBP 10022	Brechlin et al. 2016a; CRBP
Hylesia (Hylesia) arianae Brechlin, 2016		Vl	BC-RBP 8060	Brechlin et al. 2016a; CRBP
Hylesia (Hylesia) ascolombex Brechlin & Meister, 2016	+	An, By	TL; BC-RBP 8775	Brechlin et al. 2016a; CRBP
Hylesia (Hylesia) ascucayalex Brechlin & Meister, 2016		By	BC-RBP 10327	Brechlin et al. 2016a; CRBP
Hylesia (Hylesia) bouvereti Dognin, 1889		An, By, Cc, Cl, Cu, Ri, St, To, Vl	BC-RBP 8236	Amarillo-Suárez 2000; Muñoz and Amarillo-Suárez 2010; CRBP
Hylesia (Hylesia) canandex Brechlin & van Schayck, 2016		Am	IAvH-E-190405	Brechlin et al. 2016a; Jiménez-Bolívar et al. 2021; CRBP
Hylesia (Hylesia) caucanex Draudt, 1929	+	Cc	TL	Draudt 1929; Lemaire 2002 as *H.coex*; Brechlin et al. 2016a; ANDES-E, CRBP
Hylesia (Hylesia) cesarex Brechlin, 2022	+	Ce	TL; BC-RBP 10342	Brechlin 2022h; CRBP
Hylesia (Hylesia) colombex Dognin, 1923	+	Ch, Vl	TL	Dognin 1923; Amarillo-Suárez 2000; Lemaire 2002; CRBP
Hylesia (Hylesia) compandex Brechlin & van Schayck, 2016		By, Ca, Me	BC-RBP 8965	Brechlin et al. 2016a; CRBP
Hylesia (Hylesia) composita Dognin, 1912		By, Me	BC-RBP 8821	Brechlin et al. 2016a; CRBP
Hylesia (Hylesia) compsantandex Brechlin & Meister, 2016	+	By, St	TL; BC-RBP 9596	Brechlin et al. 2016a; CRBP
Hylesia (Hylesia) continua colombiana Dognin, 1922		An, By, Cc, Ch, Gj, Ma, Ri, St, Vl	BC-RBP 7941	Dognin 1922; Lemaire 2002; Decaëns et al. 2003b, 2007; Amarillo-Suárez 2000 and Muñoz and Amarillo-Suárez 2010 as *H.continua*; ANDES-E, CRBP
Hylesia (Hylesia) cotmetex Brechlin & Meister, 2016	+	Me	TL; BC-RBP 8770	Brechlin et al. 2016a; CRBP
Hylesia (Hylesia) dalina Schaus, 1911		An, By, Ce, Ch, Ma, Vl	IAvH-E-186772	Amarillo-Suárez 2000; Lemaire 2002; CRBP
Hylesia (Hylesia) ebalus ebalus (Cramer, 1775)		An, By, Cu, Ca, Cc, Me	BC-RBP 8966	Amarillo-Suárez 2000; Lemaire 2002; CRBP
Hylesia (Hylesia) ebalus margarita Dognin, 1901		An, Cc	TL; BC-RBP 8671	Dognin 1901; Lemaire 2002; CRBP
Hylesia (Hylesia) fabiani elorex Brechlin, 2016		Vl	BC-RBP 10718	Brechlin et al. 2016a; CRBP
Hylesia (Hylesia) faunalex Brechlin & Meister, 2016	+	Ce, Ma	TL; BC-RBP 9223	Brechlin et al. 2016a; CRBP
Hylesia (Hylesia) garrochex Brechlin & Meister, 2016	+	An	TL; BC-RBP 9218	Brechlin et al. 2016a; CRBP
Hylesia (Hylesia) gigantex Draudt, 1929		Ch, St, Vl	TL; BC-RBP 9589	Draudt 1929; Amarillo-Suárez 2000; Lemaire 2002; CRBP
Hylesia (Hylesia) gyramazonex Brechlin & Meister, 2016		Me, Pu		Lemaire 2002 and Jiménez-Bolívar et al. 2021 as *H.gyrex*; Brechlin et al. 2016a; CRBP
Hylesia (Hylesia) ilsantandex Brechlin & Meister, 2016	+	An, By, Cu, St	TL; BC-RBP 9209	Brechlin et al. 2016a; CRBP
Hylesia (Hylesia) indandex Brechlin & Meister, 2016		Am, Me, Pu		Lemaire 2002 as *H.indurata*; Jiménez-Bolívar et al. 2021; CRBP
Hylesia (Hylesia) invidiosa Dyar, 1914		An, By, Ch, Cu, St	BC-RBP 9379	CRBP
Hylesia (Hylesia) juprex Brechlin & Meister, 2016	+	By	TL; BC-RBP 8818	Brechlin et al. 2016a; CRBP
Hylesia (Hylesia) leilex leilseptentridex Brechlin & Käch, 2016		Cc	BC-RBP 11831	Brechlin et al. 2016a; CRBP
Hylesia (Hylesia) limonex Brechlin & Käch, 2016		Cc	BC-RBP 11835	Brechlin et al. 2016a; CRBP
Hylesia (Hylesia) magdalenex Brechlin & Meister, 2016		An, By, Ma	BC-RBP 9190	Brechlin et al. 2016a; CRBP
Hylesia (Hylesia) medifex Dognin, 1916	+	By, Cu, Ma, St	TL; BC-RBP 7940	Dognin 1916; Amarillo-Suárez 2000; Lemaire 2002; Decaëns et al. 2007; ANDES-E, CRBP
Hylesia (Hylesia) melanostigma (Herrich-Schäffer, 1855)		Am, By, Ca	BC-RBP 9592	Amarillo-Suárez 2000; Lemaire 2002; ANDES-E, CRBP
Hylesia (Hylesia) metabus (Cramer, 1775)		By, Cn, Me	BC-RBP 9588	Jiménez-Bolívar et al. 2021; CRBP
Hylesia (Hylesia) metrex Brechlin & Meister, 2016		By, Me	TL; BC-RBP 8819	Brechlin et al. 2016a; CRBP
Hylesia (Hylesia) mincex mincex Brechlin & Meister, 2016	§	Ce, Ma	TL; BC-RBP 8658	Brechlin et al. 2016a; CRBP
Hylesia (Hylesia) moronensis Lemaire, 1976		By, Me	BC-RBP 8998	CRBP
Hylesia (Hylesia) moronex Brechlin & Käch, 2016		By, Me	BC-RBP 9800	Brechlin et al. 2016a; CRBP
Hylesia (Hylesia) murex Dyar, 1913		An, By, Ca, Cc, Me	BC-RBP 11836	CRBP
Hylesia (Hylesia) mymex Dyar, 1913		Cc, Vl	TL	Dyar 1913; Amarillo-Suárez 2000; Lemaire 2002; Muñoz and Amarillo-Suárez 2010; CRBP
Hylesia (Hylesia) mymsantandex Brechlin, 2022	+	By, St	TL; BC-RBP 10717	Decaëns et al. 2007 as *H.mymex*; Brechlin 2022h; CRBP
Hylesia (Hylesia) nigripes Draudt, 1929	+	By	TL	Draudt 1929; Lemaire 2002
Hylesia (Hylesia) olivenca Schaus, 1927		Me		Brechlin et al. 2016a; CRBP
Hylesia (Hylesia) olloretex Brechlin & van Schayck, 2016		Ca	BC-Dec1637	Brechlin et al. 2016a; Jiménez-Bolívar et al. 2021; CRBP
Hylesia (Hylesia) palcazua Schaus, 1927		Pu		Brechlin and Comoglio 2023e; CRBP
Hylesia (Hylesia) panguanex Brechlin & van Schayck, 2016		Am	IAvH-E-190371	Brechlin et al. 2016a; Jiménez-Bolívar et al. 2021; CRBP
Hylesia (Hylesia) pauppichinchex Brechlin & Käch, 2016		By, Ma	BC-RBP 10328	Brechlin et al. 2016a; Jiménez-Bolívar et al. 2021; CRBP
Hylesia (Hylesia) paupseptentridex Brechlin & van Schayck, 2016		By, Ca, Me	BC-RBP 8997	Brechlin et al. 2016a; CRBP
Hylesia (Hylesia) praeda Dognin, 1901		Am, By?, Cu?, Me		Lemaire 2002; Decaëns et al. 2007; CRBP
Hylesia (Hylesia) praedperuana Brechlin & Meister, 2016		Am	IAvH-E-190365	Brechlin et al. 2016a; CRBP
Hylesia (Hylesia) praedpichinchensis Brechlin & Käch, 2016		An, Ch, Vl	BC-RBP 10016	Amarillo-Suárez 2000; Lemaire 2002 as *H.praeda*; Brechlin et al. 2016a; CRBP
Hylesia (Hylesia) remcarabobex Brechlin & van Schayck, 2016		Me	BC-RBP 9802	Brechlin et al. 2016a; CRBP
Hylesia (Hylesia) rosacea thaumex Draudt, 1929		An, Ch, Vl	TL; BC-RBP 9214	Draudt 1929; Lemaire 2002; Decaëns et al. 2003b; Amarillo-Suárez 2000 and Prada-Lara et al. 2019 as *H.rosacea*; CRBP
Hylesia (Hylesia) rosbaguanex Brechlin, Meister & van Schayck, 2016		Cc	BC-RBP 11233	Brechlin et al. 2016a; CRBP
Hylesia (Hylesia) roseata Dognin, 1914		By, Cu, Pu, Ri, St, To, Vl	BC-RBP 8800	Amarillo-Suárez 2000; Lemaire 2002; CRBP
Hylesia (Hylesia) rubrifrons rubrifrons Schaus, 1911		An, Vl	BC-RBP 10710	Brechlin et al. 2016a; CRBP
Hylesia (Hylesia) rubrifrons muzoensis Draudt, 1929	§	By, Cu	TL	Draudt 1929; Amarillo-Suárez 2000; Lemaire 2002
Hylesia (Hylesia) rubriprocta Bouvier, 1930	+	Me	TL; BC-RBP 9586	Bouvier 1930; CRBP
Hylesia (Hylesia) santamartex Brechlin, 2022	+	Gj, Ma	TL; BC-RBP 11232	Brechlin 2022h; CRBP
Hylesia (Hylesia) santboyacex Brechlin & Meister, 2016	+	By, Cu, St	TL; BC-RBP 9590	Brechlin et al. 2016a; CRBP
Hylesia (Hylesia) sucumbex Brechlin & Käch, 2016		Me	BC-RBP 9903	Brechlin et al. 2016a; CRBP
Hylesia (Hylesia) tapareba tapgarrochex Brechlin & Meister, 2016	§	An	TL; BC-RBP 9213	Brechlin et al. 2016a; CRBP
Hylesia (Hylesia) tapboyacex Brechlin, 2022	+	By	TL; BC-RBP 10298	Brechlin 2022h; CRBP
Hylesia (Hylesia) tatamex Brechlin & Meister, 2016	+	Qu, Ri	TL; BC-RBP 9688	Brechlin et al. 2016a; CRBP
Hylesia (Hylesia) termoronex Brechlin & Käch, 2016		Me	BC-RBP 10543	Brechlin et al. 2016a; CRBP
Hylesia (Hylesia) terrocaquetex Brechlin & Comoglio, 2023	+	Ca	TL; BC-RBP 12810	Brechlin and Comoglio 2023e; CRBP
Hylesia (Hylesia) terrosex Dognin, 1916		Pu		Brechlin and Comoglio 2023e; CRBP
Hylesia (Hylesia) tersucumbex Brechlin, 2022		Cc		Brechlin 2022h; CRBP
Hylesia (Hylesia) umbrata (Schaus, 1911)		An, Ch, Cl, Ma, To, Vl	BC-RBP 9771	Amarillo-Suárez 2000; Lemaire 2002; Decaëns et al. 2003b; CRBP
Hylesia (Hylesia) yarumalex Brechlin & Meister, 2016	+	An	TL; BC-RBP 9217	Brechlin et al. 2016a; CRBP
Hylesia (Hylesia) yuyapichrex Brechlin & Meister, 2016		Cn	IAvH-E-190287	Brechlin et al. 2016a; Jiménez-Bolívar et al. 2021; CRBP
Hylesia (Hylesia) zonex Draudt, 1929	+	Cu	TL	Draudt 1929; Lemaire 2002
Subgenus Micrattacus Walker, 1855				
Hylesia (Micrattacus) nanus (Walker, 1855)		By, Ca, Ch, Cu, Me, Na, Ri, Vl	BC-RBP 8911	Lemaire 2002; Decaëns et al. 2003b; 2007; ANDES-E, CRBP
Genus *Hylesiopsis* Bouvier, 1929				
*Hylesiopsisfestiva* Bouvier, 1929		By, Me, Pu	BC-RBP 12662	Amarillo-Suárez 2000; Lemaire 2002; CRBP
Genus *Hyperchiria* Hübner, 1819 [1816]				
*Hyperchiriacolumbiana* Brechlin & Meister, 2010		An, By	TL; BC-RBP-2268	Brechlin and Meister 2010c; ANDES-E, CRBP
*Hyperchirianausimetensis* Brechlin, 2019	+	Me	TL; BC-RBP 9566	Brechlin 2019f; CRBP
*Hyperchirianausioccidentalis* Brechlin & Meister, 2010		Am, Ca, Cc, Me	BC-RBP 11224	Brechlin and Meister 2010c; ANDES-E, CRBP
*Hyperchiriaparacuta* Brechlin, 2019		Pu		Brechlin 2019f; CRBP
*Hyperchiriaparallela* Brechlin, Käch & Meister, 2011		An, Na	BC-RBP 10290	Brechlin et al. 2011b; CRBP
*Hyperchiriavolcana* Brechlin, Käch & Meister, 2011		Vl	BC-RBP 10695	Brechlin et al. 2011b; CRBP
*Hyperchiriawinbrechlini* Brechlin, 2019	+	Hu	TL; BC-RBP 11125	Brechlin 2019f; CRBP
Genus *Leucanella* Lemaire, 1969				
*Leucanellaaltolima* Brechlin, 2021	+	To	TL; BC-RBP 11935	Brechlin 2021c; CRBP
*Leucanellaapollinairei* (Dognin, 1923)	+	Cn, Me	TL; BC-Dec1460	Dognin 1923; Amarillo-Suárez 2000; Lemaire 2002
*Leucanellaarcoccidentalis* Brechlin, 2022	+	Ch, Ri	TL; BC-RBP 12416	Brechlin 2022d; CRBP
*Leucanellaarctioquia* Brechlin, 2021	+	An	TL; BC-RBP 9819	Brechlin 2021c; CRBP
*Leucanellaarcuata* Brechlin & Meister, 2012		Ca	BC-RBP 12753	Brechlin and Meister 2012a; CRBP
*Leucanellabolanosi* Brechlin, Käch & Meister, 2013		Na	BC-RBP 12256	Amarillo-Suárez 2000 as *L.nyctimene*; Brechlin 2021c; CRBP
*Leucanellabonillensis* Decaëns & Rougerie, 2008	+	An?, By	TL; BC-RBP 9871	Decaëns and Rougerie 2008; ANDES-E, CRBP
*Leucanellacontempta* (Lemaire, 1967)	+	An, Cl, Qu, Ri, Vl	TL; BC-RBP 11947	Lemaire 1966a, 2002; Amarillo-Suárez 2000; Brechlin 2021c; ANDES-E, CRBP
*Leucanellaflammans* (Schaus, 1900)		Cc, Ch, Na, Vl	TL	Schaus 1900; Amarillo-Suárez 2000; Lemaire 2002; ANDES-E, CRBP
*Leucanellalynx* (Bouvier, 1930)		Na, Pu	BC-RBP 11941	CRBP
*Leucanellamaandensis* Brechlin & Meister, 2011		By, Cn, Me	BC-Dec0535	Brechlin and Meister 2011h; CRBP
*Leucanellaneglecta* Brechlin & Meister, 2012		Cc	BC-RBP 11246	Brechlin and Meister 2012a; CRBP
*Leucanellaneomene* Brechlin, 2021	+	By, Cu, St	TL; BC-RBP 11948	Brechlin 2021c; ANDES-E, CRBP
*Leucanellanyctimene* (Latreille, 1832)	+	Cu	TL; BC-RBP 5407	Latreille 1832; Amarillo-Suárez 2000; Lemaire 2002; Brechlin 2021c; ANDES-E, CRBP
*Leucanellanyctimenoides* (Lemaire, 1967)		By, Ce, Cu, St	BC-RBP 11950	Brechlin 2021c; CRBP
*Leucanellasantamartensis* Brechlin, 2021	+	Ce	TL; BC-RBP 11939	Brechlin 2021c; CRBP
*Leucanellatolimaiana* Brechlin, 2021	+	Ca, Cc, To	TL; BC-RBP 8355	Amarillo-Suárez 2000 and Lemaire 2002 as *L.nyctimene*; Brechlin 2021c; CRBP
Genus *Molippa* Walker, 1855				
*Molippaazuelensis* Lemaire, 1976		An, Ca, Cl, Hu, Ri, To	BC-RBP 8019	CRBP
*Molippabasina* Maassen & Weyding, 1885		Cn, Cu, Gj, Hu, Ma, Me	BC-RBP 8659	CRBP
*Molippaflavotegana* Brechlin & Meister, 2011		By, Ch, Ma, Ri, St, Vl	BC-RBP 8967	Decaëns et al. 2003b, 2007, and Prada-Lara et al. 2019 as *M.nibasa*; Brechlin and Meister 2011a; ANDES-E, CRBP
*Molippaintermediata* Brechlin & Meister, 2011		Am, Ca	BC-Dec1592	Brechlin and Meister 2011a; CRBP
*Molippalatcolombiana* Brechlin, 2021	+	An, By, St	TL; BC-RBP 9264	Amarillo-Suárez 2000 as *M.latemedia*; Brechlin 2021a; CRBP
*Molippalatemedia* (Druce, 1890)		Ca, Me, Pu	BC-RBP 6349	Racheli and Vinciguerra 2005; CRBP
*Molippaplacnapoana* Brechlin & Meister, 2014		Cc	BC-RBP 11223	Brechlin and Meister 2014a; CRBP
*Molippasimandensis* Brechlin, 2021		Am, Ca	BC-Dec1695	Racheli and Vinciguerra 2005 as “Molippasp. nearsimillima”; Brechlin 2021a; CRBP
*Molippatusina* (Schaus, 1921)		Ch, Na, Vl	BC-RBP 12663	Amarillo-Suárez 2000; Lemaire 2002; CRBP
*Molippavladislavi* Brechlin & Meister, 2014		Pu	TL; BC-RBP 6348	Brechlin and Meister 2014a; CRBP
Genus *Pseudautomeris* Lemaire, 1967				
*Pseudautomerisantioquia* (Schaus, 1921)	+	An	TL; BC-RBP 8672	Schaus 1921; Amarillo-Suárez 2000; Lemaire 2002; CRBP
*Pseudautomerischocensis* Brechlin & Meister, 2013	+	Ch	TL; BC-RBP 4883	Decaëns et al. 2003b as *P.irene*; Brechlin et al. 2013f; CRBP
*Pseudautomerisfrontino* Brechlin, 2022	+	An	TL; BC-RBP 12264	Brechlin 2022f; CRBP
*Pseudautomerishorsti* Brechlin & Meister, 2013		Ca, Cc	BC-RBP 10985	Brechlin et al. 2013f; CRBP
*Pseudautomerislata* (Conte, 1906)		Cc, Cu, Pu		CRBP
*Pseudautomerisrudloffirudecuatorialis* Brechlin, 2016		Ch	BC-RBP 11317	Brechlin 2016d; CRBP
*Pseudautomerissalmcolombiana* Brechlin, 2016		Cu	TL; BC-RBP 3270	Brechlin 2016d; CRBP
*Pseudautomerisubalensis* Brechlin, 2018	+	Cu	TL; BC-RBP 11196	Brechlin 2018l; CRBP
*Pseudautomeriswinbrechlini* Brechlin, 2016		Ch, Na, Vl	BC-RBP 10694	Amarillo-Suárez 2000, Lemaire 2002, and Decaëns et al. 2003b as *P.antioquia*; Brechlin 2016d; CRBP
Subtribe Hemileucina Grote & Robinson, 1866				
Genus *Cerodirphia* Michener, 1949				
*Cerodirphiacandida* Lemaire, 1969	+	Ch, Ma, Vl	TL; BC-RBP 10618	Lemaire 1969; Amarillo-Suárez 2000; Decaëns et al. 2003b; Prada-Lara et al. 2019; ANDES-E, CRBP
*Cerodirphiafabiani* Brechlin, 2016	+	An	TL; BC-RBP 8675	Brechlin 2016a; CRBP
*Cerodirphiaflammans* Lemaire, 1973		Ch, Vl	TL	Lemaire 1972; Amarillo-Suárez 2000
*Cerodirphiafrontino* Brechlin, 2022	+	An	TL; BC-RBP 12272	Brechlin 2022j; ANDES-E, CRBP
*Cerodirphiagachala* Brechlin, 2017	+	Cu	TL; BC-RBP 9329	Brechlin 2017h; CRBP
*Cerodirphiagiustii* Brechlin, 2018	+	An, Ri, To?	TL; BC-RBP 8257	Lemaire 2002 as *C.mota*; Brechlin 2018f; ANDES-E, CRBP
*Cerodirphiakaechi* Brechlin, 2016		Na	BC-RBP 12640	Brechlin and Comoglio 2023g; CRBP
*Cerodirphiakattyana* Brechlin, 2022		Pu		Brechlin 2022j; CRBP
*Cerodirphiamota* (Druce, 1909)	+	Vl	TL	Druce 1909; Amarillo-Suárez 2000; Lemaire 2002
*Cerodirphiamotcaquetana* Brechlin & Comoglio, 2023	+	Ca	TL; BC-RBP 12619	Brechlin and Comoglio 2023g; CRBP
*Cerodirphiamotcaucensis* Brechlin, 2018	+	Vl	TL; BC-RBP 10625	Brechlin 2018f; CRBP
*Cerodirphiamotfrontino* Brechlin & Comoglio, 2023	+	An	TL; BC-RBP 12342	Brechlin and Comoglio 2023g; CRBP
*Cerodirphiamothuilana* Brechlin, 2018	+	Hu	TL; BC-RBP 10994	Brechlin 2018h; CRBP
*Cerodirphiamotjardina* Brechlin, 2022	+	An	TL; BC-RBP 12343	Brechlin 2022j; CRBP
*Cerodirphiamotpeggyae* Brechlin, 2022	+	An	TL; BC-RBP 12279	Brechlin 2022j; CRBP
*Cerodirphiapachona* (Draudt, 1929)	+	Cu, St	TL; BC-RBP 8280	Draudt 1929; Lemaire 2002; Brechlin 2016a; CRBP
*Cerodirphiapuracana* Brechlin, 2018	+	Hu	TL; BC-RBP 11187	Brechlin 2018h; CRBP
*Cerodirphiaroseamazonica* Brechlin & Meister, 2011		By, Cn, Me	BC-RBP 11654	Brechlin 2011; CRBP
*Cerodirphiasiriae* Brechlin & Meister, 2011		Am, Ca, Cu, Pu	BC-RBP 3257	Lemaire 2002 and Racheli and Vinciguerra 2005 as *C.speciosa*; Racheli and Vinciguerra 2005 as *C.brunnea*; Brechlin 2011; CRBP
*Cerodirphiazulemae* Decaëns & Rougerie, 2008	+	By, St	TL; BC-RBP 9391	Decaëns and Rougerie 2008; CRBP
Genus *Dirphia* Hübner, 1819 [1816]				
*Dirphiaabhorca* Lemaire, 1969	+	Na, Vl	TL; BC-RBP 10617	Lemaire 1969; Amarillo-Suárez 2000; CRBP
*Dirphiaaculecuatoriana* Brechlin, Meister & Käch, 2011		Cc, Cu, Me, Pu	BC-RBP 8294	Brechlin and Meister 2011d; CRBP
*Dirphiaantkozlovi* Brechlin, 2022	+	By, Cn	TL; BC-RBP 12291	Brechlin 2022k; CRBP
*Dirphiaavichoco* Brechlin & Meister, 2011		Ch	TL; BC-FMP-0278	Decaëns et al. 2003b as *D.avia*; Brechlin and Meister 2011d; CRBP
*Dirphiaaviluisiana* Brechlin & Meister, 2011	+	An, By, Cu, Hu, St	TL; BC-RBP 3772	Amarillo-Suárez 2000 as *D.avia*; Brechlin and Meister 2011d; ANDES-E, CRBP
*Dirphiaavinapoana* Brechlin, Meister & Käch, 2011		By, Ca, Cc	BC-RBP 12114	Brechlin and Meister 2011d; CRBP
*Dirphiaaviurica* Brechlin & Meister, 2011		An, By, Ma, Me, Vl	BC-RBP 3768	Brechlin and Meister 2011d; ANDES-E, CRBP
*Dirphiabrevifurca* Strand, 1911		Cc, Pu		CRBP
*Dirphiacarimaguensis* Decaëns, Bonilla & Naumann, 2005	+	Cn, Me	TL; BC-FMP-0309	Amarillo-Suárez 2000 as *D.tarquinia*; Decaëns et al. 2004a; CRBP
*Dirphiaconcolor* Walker, 1855		Cn, Gv, Me	PCG6	Jiménez-Bolívar et al. 2021 as *D.avia*; ANDES-E, CRBP
*Dirphiacrassgachala* Brechlin, 2017	+	By, Cu	TL; BC-RBP 10030	Brechlin 2017j; ANDES-E, CRBP
*Dirphiadiana* Brechlin, 2017	+	An	TL; BC-RBP 8643	Brechlin 2017j; CRBP
*Dirphiafraterna* (C. Felder & R. Felder, 1874)		Am, Ca, Hu, Me, Pu	BC-RBP 11899	Amarillo-Suárez 2000; Lemaire 2002; Racheli and Vinciguerra 2005; CRBP
*Dirphiafratmetana* Brechlin, 2021	+	Me	TL; BC-RBP 11815	Brechlin 2021h; CRBP
*Dirphiaguacana* Brechlin, 2020	+	St	TL; BC-RBP 10655	Brechlin 2020b; CRBP
*Dirphiajardina* Brechlin, 2021	+	An	TL; BC-RBP 12277	Brechlin 2021h; CRBP
*Dirphialudmillae* Lemaire, 1974	+	Ch, Vl	TL; BC-RBP 10616	Lemaire 1974, 2002; Amarillo-Suárez 2000; Decaëns et al. 2003b; CRBP
*Dirphialudyarumala* Brechlin, 2017	+	An, Cl, Ri	TL; BC-RBP 8652	Brechlin 2017j; ANDES-E, CRBP
*Dirphianora* (Druce, 1897)		Ch	BC-Dec0916	CRBP
*Dirphiapacifica* Lemaire, 1981	+	Ch, Vl	TL; BC-RBP 12292	Lemaire 1981, 2002; Brechlin 2022k; ANDES-E, CRBP
*Dirphiapanamensis* (Schaus, 1921)		Gj, Hu, Pu	BC-RBP 11639	Lemaire 2002; CRBP
*Dirphiaradandensis* Brechlin, 2017		Am, Pu	BC-RBP 12507	Brechlin 2017j; CRBP
*Dirphiaradinirida* Brechlin & Comoglio, 2023	+	Gn	TL; BC-RBP 12515	Jiménez-Bolívar et al. 2021 as *D.radiata*; Brechlin and Comoglio 2023d; ANDES-E, CRBP
*Dirphiasantboyacensis* Brechlin, 2017	+	By, St	TL; BC-RBP 8010	Brechlin 2017j; ANDES-E, CRBP
*Dirphiasomniculosa* (Cramer, 1777)		By, Cu, Ma, NS	BC-RBP 8996	Lemaire 2002; Decaëns et al. 2007; ANDES-E, CRBP
*Dirphiasomoccidentalis* Brechlin, Käch & Meister, 2013		Ch, Vl	BC-Dec0930	Lemaire 2002, Decaëns et al. 2003b, and Prada-Lara et al. 2019 as *D.somniculosa*; Brechlin et al. 2013e; CRBP
*Dirphiasubhorca* Dognin, 1901		Ch, Na, Vl	BC-Dec0738	Amarillo-Suárez 2000; CRBP
*Dirphiatarquinia* (Cramer, 1775)		Gn		ANDES-E
*Dirphiathliptophana* (C. Felder & R. Felder, 1874)		Am, Ca, Hu, Me, Pu	BC-RBP 11816	Amarillo-Suárez 2000; CRBP
*Dirphiatolimafurca* Brechlin & Meister, 2011	+	Ca, Hu, To	TL; BC-RBP 3234	Brechlin and Meister 2011d; CRBP
*Dirphiayarumala* Brechlin, 2017	+	An, Cl, Ri	TL; BC-RBP 8653	Brechlin 2017j; CRBP
Genus *Dirphiella* Michener, 1949				
*Dirphiellaniobe* (Lemaire, 1978)		Na?		Lemaire 2002
Genus *Dirphiopsis* Bouvier, 1928				
*Dirphiopsisflora* (Schaus, 1911)		Cc, Ch, Na, Vl	BC-RBP 10697	Amarillo-Suárez 2000; Decaëns et al. 2003b; CRBP
*Dirphiopsisorientalis* Lemaire, 1976		By, Ca, Cc, Pu	BC-RBP 11225	Decaëns et al. 2007 as *D.flora*; CRBP
*Dirphiopsispulchriboyacensis* Brechlin & Meister, 2018	+	By, Cu	TL; BC-RBP 9204	Brechlin and Meister 2018; CRBP
*Dirphiopsispulchventanas* Brechlin & Meister, 2019	+	St	TL; BC-RBP 10778	Brechlin and Meister 2018; CRBP
*Dirphiopsisrotenbergi* Brechlin & Meister, 2011		Me	BC-RBP 9997	Brechlin and Meister 2011e; CRBP
Genus *Meroleuca* Packard, 1904				
Subgenus Dihirpa Draudt, 1929				
Meroleuca (Dihirpa) campanario Brechlin, 2018	+	Hu, To	TL; BC-RBP 8262	Brechlin 2018p; CRBP
Meroleuca (Dihirpa) frontino Brechlin, 2021	+	An	TL; BC-RBP 12238	Brechlin 2021m; ANDES-E, CRBP
Meroleuca (Dihirpa) litura (Walker, 1855)	+	By, Cu, St	TL; BC-RBP 8245	Walker 1855b; Amarillo-Suárez 2000; Lemaire 2002; ANDES-E, CRBP
Meroleuca (Dihirpa) ristolima Brechlin, 2018	+	An, Ri, To	TL; BC-RBP 9892	Brechlin 2018p; ANDES-E, CRBP
Subgenus Meroleuca Packard, 1904				
Meroleuca (Meroleuca) lituroides (Bouvier, 1929)	+	By, Cu	TL	Bouvier 1929; Amarillo-Suárez 2000; Lemaire 2002
Meroleuca (Meroleuca) nigra (Dognin, 1913)	+	Cu	TL; BC-RBP 12614	Dognin 1913; Amarillo-Suárez 2000; Lemaire 2002; CRBP
Meroleuca (Meroleuca) venosa (Walker, 1855)	+	Cu, St	TL	Walker 1855b; Amarillo-Suárez 2000; Lemaire 2002
Subgenus Meroleucoides Michener, 1949				
Meroleuca (Meroleucoides) amarillae Lemaire & Wolfe, 1995	+	By, St	TL; BC-RBP 8009	Lemaire and Wolfe 1995; Amarillo-Suárez 2000; Lemaire 2002; CRBP
Meroleuca (Meroleucoides) belmirana Brechlin, 2021	+	An	TL; BC-RBP 12241	Brechlin 2021m; CRBP
Meroleuca (Meroleucoides) cabrera Brechlin, 2018	+	Cu	TL; BC-RBP 10090	Brechlin 2018i; CRBP
Meroleuca (Meroleucoides) cabreroides Brechlin, 2018	+	Cu	TL; BC-RBP 10825	Brechlin 2018c; CRBP
Meroleuca (Meroleucoides) dargei Lemaire, 1982	+	St	TL	Lemaire 1982, 2002; Amarillo-Suárez 2000
Meroleuca (Meroleucoides) fabiani Brechlin, 2018	+	Pu	TL; BC- RBP 10981	Brechlin 2018c; CRBP
Meroleuca (Meroleucoides) fassli Lemaire, 1995	+	Cl, To	TL; BC-RBP 8274	Lemaire 1995, 2002; Amarillo-Suárez 2000; Decaëns et al. 2004b as *M.diazmaurini*; CRBP
Meroleuca (Meroleucoides) fassvicente Brechlin, 2018	+	An, Cl, Ri	TL; BC-RBP 8327	Brechlin 2018i; CRBP
Meroleuca (Meroleucoides) flavodiscata (Dognin, 1916)	+	To	TL	Dognin 1916; Amarillo-Suárez 2000; Lemaire 2002
Meroleuca (Meroleucoides) guanacasa Brechlin, 2023	+	Cc	TL; BC-RBP 12269	Brechlin 2023g; CRBP
Meroleuca (Meroleucoides) elcarmenensis Brechlin & Comoglio, 2023	+	Ch	TL	Brechlin and Comoglio 2023b; CRBP
Meroleuca (Meroleucoides) machadoi Brechlin, 2018	+	Qu, Vl	TL; BC-RBP 9321	Brechlin 2018i; CRBP
Meroleuca (Meroleucoides) manizalesa Brechlin, 2020	+	An, Cl	TL; BC-RBP 11669	Brechlin 2020h; CRBP
Meroleuca (Meroleucoides) marmolana Brechlin, 2018	+	Hu	TL; BC-RBP 11176	Brechlin 2018c; CRBP
Meroleuca (Meroleucoides) marquezae Brechlin, 2018	+	By, Cu	TL; BC-RBP 9040	Brechlin 2018i; CRBP
Meroleuca (Meroleucoides) naias (Bouvier, 1929)	+	Cu	TL	Bouvier 1929; Amarillo-Suárez 2000; Lemaire 2002
Meroleuca (Meroleucoides) perijana Brechlin & Comoglio, 2023	+	Ce	TL; BC-RBP 12615	Brechlin and Comoglio 2023b; CRBP
Meroleuca (Meroleucoides) pinzonica Brechlin, 2018	+	By	TL; BC-RBP 8326	Brechlin 2018i; CRBP
Meroleuca (Meroleucoides) puracana Brechlin, 2020	+	Cc	TL; BC-RBP 11809	Brechlin 2020h; CRBP
Meroleuca (Meroleucoides) rectilineata Lemaire & Venedictoff, 1989		Na	BC-RBP 12513	CRBP
Meroleuca (Meroleucoides) soata Brechlin, 2018	+	By	TL; BC-RBP 10566	Brechlin 2018i; CRBP
Meroleuca (Meroleucoides) sochensis Brechlin, 2018	+	Cu	TL; BC-RBP 11168	Brechlin 2018c; CRBP
Meroleuca (Meroleucoides) urrao Brechlin & Comoglio, 2023	+	An	TL	Brechlin and Comoglio 2023b; CRBP
Genus *Paradirphia* Michener, 1949				
*Paradirphiaantonia* (Dognin, 1911)	+	Vl	TL; BC-RBP 10619	Dognin 1911a; Amarillo-Suárez 2000; Lemaire 2002; CRBP
*Paradirphiacabrera* Brechlin & Meister, 2017	+	Cu	TL; BC-RBP 10099	Brechlin and Meister 2017; ANDES-E, CRBP
*Paradirphiacaldas* Brechlin & Meister, 2017	+	Cl, Ri	TL; BC-FMP-0347	Brechlin and Meister 2017; CRBP
*Paradirphiacavichensis* Brechlin & Meister, 2017	+	By	TL; BC-RBP 10375	Decaëns et al. 2007 as *P.oblita*; Brechlin and Meister 2017; CRBP
*Paradirphiacundala* Brechlin, 2022	+	Cu	TL; BC-RBP 10999	Brechlin 2022a; CRBP
*Paradirphiaflorenciana* Brechlin & Comoglio, 2023	+	Ca	TL; BC-RBP 12813	Brechlin and Comoglio 2023f; CRBP
*Paradirphiafrontino* Brechlin, 2022	+	An	TL; BC-RBP 12263	Brechlin 2022a; CRBP
*Paradirphiagencarchensis* Brechlin, 2022		Na	BC-RBP 12658	Brechlin 2022a; Brechlin and Comoglio 2023f; CRBP
*Paradirphiajardina* Brechlin, 2022	+	An	TL; BC-RBP 12307	Brechlin 2022a; CRBP
*Paradirphianeivana* Brechlin, 2022	+	Ca, Hu	TL; BC-RBP 11198	Brechlin 2022a; Brechlin and Comoglio 2023f; CRBP
*Paradirphiapitalitana* Brechlin, 2022	+	Hu	TL; BC-RBP 11185	Brechlin 2022a; CRBP
*Paradirphiasantander* Brechlin & Meister, 2017	+	St	TL; BC-RBP 10059	Brechlin and Meister 2017; CRBP
*Paradirphiatatama* Brechlin & Meister, 2017	+	Ri	TL; BC-RBP 9575	Brechlin and Meister 2017; CRBP
*Paradirphiatolima* Brechlin & Meister, 2017	+	An, To	TL; BC-RBP 12392	Brechlin and Meister 2017; CRBP
*Paradirphiawinbrechlini* Brechlin, 2018	+	Ce	TL; BC-RBP 10650	Brechlin 2018g; CRBP
Genus *Periphoba* Hübner, 1820				
*Periphobacarbajal* Brechlin, 2019		Pu		CRBP
*Periphobacesar* Brechlin, 2019	+	Ce	TL; BC-RBP 10476	Brechlin et al. 2019b; CRBP
*Periphobaguajira* Brechlin, 2019	+	Gj	TL; BC-RBP 10477	Brechlin et al. 2019b; CRBP
*Periphobahuaticocha* Brechlin, 2019		Ca, Me	BC-Dec1772	Amarillo-Suárez 2000 as *P.hircia*; Brechlin et al. 2019b; CRBP
*Periphobanigra* (Dognin, 1901)		Ch, Na, Vl		Lemaire 2002; CRBP
*Periphobarudloffi* Brechlin & Meister, 2010		Ch	BC-FMP-0243	Brechlin and Meister 2010d; CRBP
*Periphobatolimaiana* Brechlin & Meister, 2010	+	An, By, Ma, St	TL; BC-RBP 3791	Amarillo-Suárez 2000 and Decaëns et al. 2007 as *P.arcaei*; Brechlin and Meister 2010d; CRBP
*Periphobatrincheras* Brechlin, Meister & van Schayck, 2019		Cn, Me	BC-FMP-0237	Brechlin et al. 2019b; CRBP
Genus *Pseudodirphia* Bouvier, 1928				
*Pseudodirphiaagandensis* Brechlin, Meister & Käch, 2011		Am, Pu		Brechlin and Meister 2011g; CRBP
*Pseudodirphiaagiyungana* Brechlin & Meister, 2011		Am	IAvH-E-190354	Brechlin and Meister 2011g
*Pseudodirphiaandicoloides* Brechlin, Meister & Käch, 2011		Pu		Brechlin and Meister 2011g; CRBP
*Pseudodirphiaangulata* Bouvier, 1929	+	Ca, Cn, Me	TL; BC-RBP 12215	Bouvier 1929; Lemaire 2002; CRBP
*Pseudodirphiabeckei* Brechlin & Meister, 2011		Cn, Me	BC-Dec0770	Brechlin and Meister 2011g; CRBP
*Pseudodirphiabireyarumala* Brechlin, 2018	+	An	TL; BC-RBP 9331	Brechlin 2018j; CRBP
*Pseudodirphiabonitala* Brechlin, 2018		Pu		Brechlin 2018j; CRBP
*Pseudodirphiabucaramangana* Brechlin, 2018	+	St	TL; BC-RBP 10614	Brechlin 2018j; CRBP
*Pseudodirphiacesar* Brechlin, 2018	+	Ce	TL; BC-RBP 10615	Brechlin 2018j; CRBP
*Pseudodirphiacomoglioi* Brechlin, 2023	+	Vl	TL; BC-RBP 12638	Brechlin 2023h; CRBP
*Pseudodirphiaconcava* Bouvier, 1929	+	By, Me	TL; BC-RBP 8668	Bouvier 1929; Brechlin 2018j; CRBP
*Pseudodirphiaconjuncta* Lemaire, 2002	+	An, By, Cu, Ma, St	TL; BC-RBP 4280	Lemaire 2002; CRBP
*Pseudodirphiacupripuncta* Lemaire, 1982	+	Cc, Ch, Vl	TL; BC-Dec0777	Lemaire 1982, 2002; Amarillo-Suárez 2000; Decaëns et al. 2003b; CRBP
*Pseudodirphiaecandides* Brechlin, 2018		Cc	BC-RBP 11220	Brechlin 2018j; CRBP
*Pseudodirphiaecoccidides* Brechlin, Meister & Käch, 2011		Pu	BC-RBP 4330	Brechlin and Meister 2011g; CRBP
*Pseudodirphiaflorenciacola* Brechlin, 2023	+	Ca	TL; BC-RBP 12780	Brechlin 2023h; CRBP
*Pseudodirphiagachacola* Brechlin, 2018	+	Cu	TL; BC-RBP 9327	Brechlin 2018j; CRBP
*Pseudodirphiagachala* Brechlin, 2021	+	Cu	TL; BC-RBP 11389	Brechlin 2021b; CRBP
*Pseudodirphiaimperialis* (Draudt, 1930)	+	Ch	TL; BC-Dec0790	Draudt 1929; Amarillo-Suárez 2000; Decaëns et al. 2003b; CRBP
*Pseudodirphiaincaquetana* Brechlin, 2023	+	Ca	TL; BC-RBP 12647	Brechlin 2023h; CRBP
*Pseudodirphiainfuscata* (Bouvier, 1924)	+	Cu?, Me?	TL	Bouvier 1924; Amarillo-Suárez 2000; Lemaire 2002
*Pseudodirphiainhuilana* Brechlin, 2018	+	Cc, Hu	TL; BC-RBP 10993	Brechlin 2018j; CRBP
*Pseudodirphiainputumayana* Brechlin, 2018	+	Pu	TL; BC-RBP 10975	Brechlin 2018j: CRBP
*Pseudodirphialeticiana* Brechlin, 2021	+	Am	TL; BC-RBP 11898	Brechlin 2021b; CRBP
*Pseudodirphiamedinensis* (Draudt, 1930)	+	Cu	TL	Draudt 1929; Lemaire 2002
*Pseudodirphiamenanderreducta* (Hering, 1925)		Cc, Ch, Cl, Na, Vl	TL; BC-RBP 10948	Hering and Hopp 1925; Lemaire 2002; Amarillo-Suárez 2000, Decaëns et al. 2003b, and Muñoz and Amarillo-Suárez 2010 as *P.menander*; ANDES-E, CRBP
*Pseudodirphiamenandersantander* Brechlin, 2018	§	St	TL; BC-RBP 9202	Brechlin 2018j; CRBP
*Pseudodirphiaobcaucana* Brechlin, 2023	+	Cc	TL; BC-RBP 12200	Brechlin 2023h; CRBP
*Pseudodirphiaobecuatoriana* Brechlin, Meister & Käch, 2011		Pu		Brechlin and Meister 2011g; CRBP
*Pseudodirphiapallida* (Walker, 1865)	+	An, Gj, Hu, To	TL; BC-RBP 8347	Walker 1865; Amarillo-Suárez 2000; Lemaire 2002; Jiménez-Bolívar et al. 2021 as *P.convexa*; CRBP
*Pseudodirphiapalmarensis* Brechlin, 2018	+	By	TL; BC-RBP 8011	Brechlin 2018j; CRBP
*Pseudodirphiaparfuscata* Brechlin, Meister & Käch, 2011		Cc	BC-RBP 11216	Brechlin and Meister 2011g; CRBP
*Pseudodirphiaregiaregia* (Draudt, 1930)		Ch, Na, Vl	TL; BC-RBP 12665	Draudt 1929; Amarillo-Suárez 2000; CRBP
*Pseudodirphiaseptentrides* Brechlin, Meister & Käch, 2011		Pu		Brechlin and Meister 2011g; CRBP
*Pseudodirphiasinuosa* Lemaire, 2002		An, By, Cl, Cu, Ma, St, To, Vl	TL; BC-RBP 12463	Lemaire 2002; Amarillo-Suárez 2000 and Decaëns et al. 2007 as *P.agis*; CRBP
*Pseudodirphiasucumbioscola* Brechlin, 2018		Cc	BC-RBP 11217	Brechlin 2018j; CRBP
*Pseudodirphiauniseptentrionalis* Brechlin, Meister & Käch, 2011		Cc, Cu, Me	BC-RBP 8330	Brechlin and Meister 2011g; CRBP
*Pseudodirphiaventanita* Brechlin, 2018	+	An, Cl, Vl	TL; BC-RBP 9332	Brechlin 2018j; CRBP
*Pseudodirphiayarumacola* Brechlin, 2018	+	An	TL; BC-RBP 10302	Brechlin 2018j; CRBP
Genus *Rhodirphia* Michener, 1949				
*Rhodirphiacarminata* (Schaus, 1902)		Cc, Na, Ri, Vl	TL; BC-RBP 10565	Schaus 1902; Amarillo-Suárez 2000; Muñoz and Amarillo-Suárez 2010; CRBP
*Rhodirphiawinbrechlini* Brechlin, 2017	+	An	TL; BC-RBP 8651	Brechlin 2017g; CRBP
Genus *Winbrechlinia* Brechlin, 2016	+			
*Winbrechliniagrissinjaevi* Brechlin, 2018	+	Ce	TL; BC-RBP 10467	Brechlin 2018m; CRBP
*Winbrechliniakitchingi* Brechlin, 2020	+	Ma	TL; BC-RBP 11428	Brechlin 2020d; CRBP
*Winbrechliniaparbrechlini* Brechlin, 2018	+	Ma	TL; BC-RBP 10525	Brechlin 2018m; CRBP
*Winbrechliniashapiroi* (Lemaire, 1978)	+	Ce	TL; BC-MNHN0001	Lemaire 1978a, 2002; Amarillo-Suárez 2000
*Winbrechliniasinjaevi* Brechlin, 2018	+	Ce	TL; BC-RBP 10356	Brechlin 2018m; CRBP
*Winbrechliniawinbrechlini* Brechlin, 2016	+	Ma	TL; BC-RBP 10208	Brechlin 2016f; CRBP
Tribe Lonomiini Bouvier, 1930				
Genus *Lonomia* Walker, 1855				
*Lonomiaachelous* (Cramer, 1777)		Am	BC-RBP 11959	González et al. 2023; ANDES-E, CRBP
*Lonomiacanescens* Brechlin & Meister, 2011		Pu		Brechlin et al. 2011c; CRBP
*Lonomiacasanarensis* Brechlin, 2017	+	Cn, Me	TL; BC-RBP 8014	Brechlin 2017i; ANDES-E, CRBP
*Lonomiacayennensis* Brechlin & Meister, 2019		Gn	CGR_Lon119	González et al. 2023; ANDES-E
*Lonomiacolumbiana* Lemaire, 1972		Na, Vl	TL; BC-RBP 12756	Lemaire 1971b, 2002; Amarillo-Suárez 2000; ANDES-E, CRBP
*Lonomiadescimoni* Lemaire, 1972		Am, Me	BC-RBP 11958	Amarillo-Suárez 2000; Lemaire 2002; González et al. 2023; ANDES-E, CRBP
*Lonomiafrontino* Brechlin, 2022		An, Ri, Vl	TL; BC-RBP 12412	Brechlin 2022e; CRBP
*Lonomialaalbania* Brechlin, 2017	+	Vl	TL; BC-RBP 8291	Brechlin 2017i; CRBP
*Lonomiaminca* Brechlin, 2017	+	Ma	TL; BC-RBP 9174	Brechlin 2017i; ANDES-E, CRBP
*Lonomiaorientoandensis* Brechlin & Meister, 2011		Am, Cn, Me, Pu	BC-RBP 8403	Brechlin et al. 2011c; ANDES-E, CRBP
*Lonomiaorientocordillera* Brechlin, Käch & Meister, 2013		Am, Cn, Me	BC-Dec0853	Brechlin and Meister 2013f; ANDES-E, CRBP
*Lonomiapuntarenasiana* Brechlin & Meister, 2011		An, By, St	BC-RBP 8649	Brechlin et al. 2011c; CRBP
*Lonomiarengifoi* Brechlin & Käch, 2017		Am, Pu	EL7179	Brechlin 2017i; CRBP
*Lonomiarufescens* Lemaire, 1972		Vl	TL; BC-MNHN0244	Lemaire 1971b, 2002; Amarillo-Suárez 2000; CRBP
*Lonomiavanschaycki* Brechlin, Käch & Meister, 2013		Cc	BC-RBP 10987	Brechlin and Meister 2013f; CRBP
*Lonomiavenezuelensis* Lemaire, 1972		An, By, Cl, Cu, Hu, St, To	BC-RBP 8655	ANDES-E, CRBP
Genus *Periga* Walker, 1855				
*Perigaagrio* Brechlin & Käch, 2018		Cc	BC-RBP 12267	Brechlin 2018q; CRBP
*Perigaangcaucana* Brechlin, 2021	+	Ca, Cc	TL; BC-RBP 11768	Racheli and Vinciguerra 2005 as *P.angulosa*; Brechlin 2021f; CRBP
*Perigaarmata* (Lemaire, 1973)	+	Cu	TL	Lemaire 1973, 2002; Amarillo-Suárez 2000
*Perigabarragani* Brechlin, Meister & Käch, 2013		Na		Brechlin 2023k; CRBP
*Perigaelsa* (Lemaire, 1973)	+	Vl	TL	Lemaire 1973, 2002; Amarillo-Suárez 2000
*Perigaextensiva* Lemaire, 2002		Cc, Me, Pu	BC-RBP 10990	Lemaire 2002; CRBP
*Perigagachala* Brechlin, 2018	+	Cu	TL; BC-RBP 9330	Brechlin 2018j; CRBP
*Perigagalbiparaculata* Brechlin, Meister & Käch, 2013		Cc	BC-RBP 11903	Brechlin and Meister 2013d; CRBP
*Perigaguaca* Brechlin, 2018	+	Ce, St	TL; BC-RBP 10658	Brechlin 2018j; CRBP
*Perigainexpectata* (Lemaire, 1972)	+	Cu, Gv, Me	TL; BC-RBP 12514	Lemaire 1971b, 2002; Amarillo-Suárez 2000; CRBP
*Perigaintensiva* (Lemaire, 1973)	+	Vl	TL	Lemaire 1973, 2002; Amarillo-Suárez 2000
*Perigakaechi* Brechlin, 2018		Na		Brechlin 2018j; CRBP
*Perigalamercedia* Brechlin, Meister & van Schayck, 2013		Am	BC-RBP 11960	Brechlin and Meister 2013d; CRBP
*Perigamincensis* Brechlin, 2018	+	Ce, Ma	TL; BC-RBP 10311	Brechlin 2018j; CRBP
*Perigaoccidentalis* (Lemaire, 1972)	+	Ch, Vl	TL; BC-RBP 10890	Lemaire 1971b, 2002; Amarillo-Suárez 2000; Decaëns et al. 2003b; CRBP
*Perigapachijalensis* Brechlin, Meister & Käch, 2013		Na	BC-RBP 12754	Brechlin and Meister 2013d; CRBP
*Perigaparvibulbacea* (Lemaire, 1972)		Pu		CRBP
*Perigaparvicaucana* Brechlin, 2022	+	Cc	TL; BC-RBP 11239	Brechlin 2022l; CRBP
*Perigaparvicitara* Brechlin, 2022	+	An	TL; BC-RBP 12424	Brechlin 2022l; CRBP
*Perigaperijana* Brechlin, 2023	+	Ce	TL; BC-RBP 12757	Brechlin 2023k; CRBP
*Perigaprattorum* (Lemaire, 1972)		Cc, Pu	BC-RBP 11238	CRBP
*Perigapuracana* Brechlin, 2020	+	Hu	TL; BC-RBP 11189	Brechlin 2020g; CRBP
*Perigasanmartiniana* Brechlin & Meister, 2013		Ca, Me	BC-Dec0842	Brechlin and Meister 2013d; Jiménez-Bolívar et al. 2021 as *P.bispinosa*; CRBP
*Perigasantandensis* Brechlin, 2018	+	By, St	TL; BC-RBP 8281	Brechlin 2018j; CRBP
*Perigaseptoccidentalis* Brechlin, 2023	+	An, Ri, Vl	TL; BC-RBP 8186	Brechlin 2023k; CRBP
*Perigatatama* Brechlin, 2018	+	Ch, Ri	TL; BC-RBP 9814	Brechlin 2018j, 2023k; CRBP
Subfamily Hirpidinae Rougerie, 2022				
Genus *Hirpida* Draudt, 1930				
*Hirpidaechuilana* Brechlin, 2023		Hu	BC-RBP 11268	Brechlin 2023f; CRBP
*Hirpidagaujoni* (Dognin, 1894)		An, Ca, Pu, To	BC-RBP 8021	Lemaire 2002; ANDES-E, CRBP
*Hirpidagauhuilana* Brechlin, 2019	+	Hu	TL; BC-RBP 11178	Brechlin 2019c; CRBP
*Hirpidagaurisaraldana* Brechlin, 2019	+	An, Qu, Ri	TL; BC-RBP 9582	Brechlin 2019c; CRBP
*Hirpidapeggyae* Brechlin, 2019	+	By, Cu, St	TL; BC-RBP 8354	Lemaire 2002 as *H.gaujoni*; Brechlin 2019c; ANDES-E, CRBP
*Hirpidasantboyacana* Brechlin, 2019		By, St	BC-RBP 10649	Brechlin 2019c; CRBP
*Hirpidatatama* Brechlin, 2019	+	Ri	TL; BC-RBP 9581	Brechlin 2019c; CRBP
*Hirpidayarumala* Brechlin, 2019	+	An, Vl	TL; BC-RBP 9665	Brechlin 2019c; CRBP
Subfamily Oxyteninae Jordan, 1924				
Genus *Homoeopteryx* C. Felder & R. Felder, 1874				
*Homoeopteryxfrontino* Brechlin, 2021	+	An	TL; BC-RBP 12236	Brechlin 2021e; CRBP
*Homoeopteryxmalecena* (Druce, 1886)		By	BC-RBP 11655	Brechlin 2021e; CRBP
*Homoeopteryxpinchcarchensis* Brechlin & Käch, 2014		Na, Ri	BC-RBP 12633	Jiménez-Bolívar et al. 2021; CRBP
Genus *Oxytenis* Hübner, 1819 [1816]				
*Oxytenisalbilunulataalbecuatoriana* Brechlin & Käch, 2014		An, Ch, Vl	BC-RBP 10292	Decaëns et al. 2003b as *O.albilunulata*; Brechlin et al. 2014; CRBP
*Oxytenisalbnapoensis* Brechlin & Käch, 2014		Am, Cc, Cu, Me		Brechlin et al. 2014; ANDES-E, CRBP
*Oxytenisbepreoides* Brechlin, 2021		By	BC-RBP 11802	Brechlin 2021j; CRBP
*Oxyteniseppinchcarchensis* Brechlin & Käch, 2014		Ri, Vl	BC-RBP 9580	Brechlin et al. 2014; CRBP
*Oxytenisepsumacensis* Brechlin & Käch, 2014		Ca, Cu, Hu, St	BC-RBP 8409	Brechlin et al. 2014; CRBP
*Oxytenisespichinchensis* Brechlin & Käch, 2014		Ch		Brechlin et al. 2014; CRBP
*Oxytenisgigantea* (Druce, 1890)		Hu	BC-RBP 11128	CRBP
*Oxytenismodestia* (Cramer, 1780)		Am, Ca, Cc, Me	BC-Dec1701	Racheli and Vinciguerra 2005; CRBP
*Oxytenismodoccidentalis* Brechlin & Käch, 2014		An, Ch, Gj, Ma	BC-RBP 8373	Brechlin et al. 2014; CRBP
*Oxytenisnaemianaemia* Druce, 1906		Am, By, Ca, Ch, Me, Pu, St	BC-RBP 8405	Decaëns et al. 2003b, 2007; CRBP
*Oxytenisnaemiajordani* Brechlin, 2021		Ce, Ch, Ma	TL; BC-RBP 10314	Decaëns et al. 2003b and Jiménez-Bolívar et al. 2021 as *O.naemiaorecta*; Brechlin 2021j; CRBP
*Oxytenisnubilanubila* Jordan, 1924		St	TL; BC-RBP 10035	Jordan 1924; CRBP
*Oxytenisnubilanuboroiana* Brechlin & Käch, 2014		Vl	BC-RBP 10689	Brechlin et al. 2014; CRBP
*Oxytenisnubnapoensis* Brechlin & Käch, 2014		By, Me	BC-RBP 8406	Brechlin et al. 2014; ANDES-E, CRBP
*Oxytenispanguana* Brechlin & Meister, 2014		Cn	IAvH-E-190421	Racheli and Vinciguerra 2005 as *O.leda*; Brechlin et al. 2014; Jiménez-Bolívar et al. 2021; CRBP
*Oxytenisperegrinaperandensis* Brechlin & Meister, 2014		Cc	BC-RBP 11262	Brechlin et al. 2014; CRBP
*Oxytenisplettina* Jordan, 1924		Vl	BC-RBP 10775	Jordan 1924; CRBP
*Oxytenissiriae* Brechlin & Käch, 2014		Pu		Brechlin et al. 2014; CRBP
*Oxytenisspadix* Jordan, 1924		Vl	BC-RBP 10628	Jordan 1924; CRBP
*Oxytenisvanmeraldas* Brechlin, 2021		Na	BC-RBP 12769	CRBP
Genus *Therinia* Hübner, 1823				
*Theriniaamphiraamphira* (Druce, 1890)		An, By, Ca, Cc, Me, Pu, St	BC-RBP 9176	Jordan 1924; CRBP
*Theriniabuckleyibuckleyi* (Druce, 1890)		Am, Ca	BC-Dec1685	Jordan 1924; ANDES-E, CRBP
*Theriniadiffissa* (Jordan, 1924)		Am, Pu		CRBP
*Theriniageometraria* (C. Felder & R. Felder, 1862)		By, Cc, Me	BC-RBP 9177	Felder and Felder 1862; Jordan 1924; CRBP
*Therinialactucinalactandensis* Brechlin & Meister, 2014		Ca, Cc	BC-Dec1705	Brechlin and Meister 2014b; CRBP
*Theriniasinae* Brechlin, 2021		By, Ch, Vl	BC-RBP 12228	Brechlin 2021k; CRBP
*Theriniaterminalis* (Jordan, 1924)		Ch, Na, Vl	BC-RBP 12235	Jordan 1924; CRBP
*Theriniatransversariacolumbiana* (Jordan, 1924)		By, Cc, Ce, Ma, Me, St	TL; BC-RBP 8359	Jordan 1924; Jiménez-Bolívar et al. 2021 as *T.t.transversaria*; CRBP
Subfamily Saturniinae Boisduval, 1837				
Tribe Attacini Blanchard, 1840				
Genus *Rothschildia* Grote, 1896				
*Rothschildiaaltomartensis* Brechlin, 2021	+	Ce, Ma	TL; BC-RBP 10218	Brechlin 2021g; CRBP
*Rothschildiaarethusarhodina* Jordan, 1911		Hu, Pu		Lemaire 1978b; CRBP
*Rothschildiaariciaaricia* (Walker, 1855)		By, Cn, Cu	TL; BC-RBP 8205	Walker 1855a; Amarillo-Suárez 2000; CRBP
*Rothschildiaaricianapoecuadoriana* Brechlin & Meister, 2010		Hu, Na, To, Vl	BC-RBP 8204	Amarillo-Suárez 2000 as *R.a.aricia*; Brechlin and Meister 2010f; CRBP
*Rothschildiaaurotaauroamazonensis* Brechlin & Meister, 2013		Me	BC-RBP 8339	Amarillo-Suárez 2000 as *R.aurota*; Brechlin and Meister 2013e; CRBP
*Rothschildiaequatorialisequatorialis* Rothschild, 1907		An, Ch, Na, Vl	TL; BC-RBP 8340	Decaëns et al. 2003b; ANDES-E, CRBP
*Rothschildiaequatorialisbogotana* Rothschild, 1907, stat. rev., comb. nov.	§	By, Cu, St	TL; BC-RBP 8206	Rothschild 1907; Brechlin 2023l; ANDES-E, CRBP
*Rothschildiaequatorialiscentricolombiana* Brechlin, 2023	§	An, By, Ma, To	TL; BC-RBP 9635	Brechlin 2023l; CRBP
*Rothschildiaerycinaerycina* (Shaw, 1796)		Am, Ca, Cc, Me	RROU00474	ANDES-E, CRBP
*Rothschildiaerycinanigrescens* Rothschild, 1907		An, By, Ch, Gj, Hu, Na	BC-RBP 11991	Amarillo-Suárez 2000; Decaëns et al. 2003b; ANDES-E, CRBP
*Rothschildiahesperus* (Linnaeus, 1758)		Am, Cc, Pu		Amarillo-Suárez 2000; CRBP
*Rothschildiaincainccolombiana* Brechlin, 2023	§	Cc, Cu, Hu, Me	TL; BC-RBP 12132	Brechlin 2023l; CRBP
*Rothschildiaincaincecuatoriana* Brechlin, Käch & Meister, 2012		Am	BC-RBP 12628	Brechlin and Meister 2012c; CRBP
*Rothschildiainccundnamarca* Brechlin, 2021	+	By, Cu	TL; BC-RBP 11998	Brechlin 2021g, 2023l; CRBP
*Rothschildialebeauaroma* Schaus, 1905		An, By, Cc, Ce, Ch, Gj, Hu, To, Vl	BC-RBP 11979	Amarillo-Suárez 2000, Decaëns et al. 2003b, and 2007 as *R.lebeauinca*; Calero-Mejía et al. 2014 as *R.lebeau* (Guérin-Méneville, 1868); CRBP
*Rothschildialebecuatoriana* Brechlin, Käch & Meister, 2012		Ch		ANDES-E, CRBP
*Rothschildialebtolimaiana* Brechlin & Meister, 2012	+	By, Cu, St, To	TL; BC-RBP 11983	Brechlin and Meister 2012c; ANDES-E, CRBP
*Rothschildiameridana* Rothschild, 1907		Cu, Me	BC-RBP 11987	CRBP
*Rothschildiaperuvianacoxeyi* Schaus, 1932		Am, Cc	BC-RBP 12670	Jiménez-Bolívar et al. 2021 as *R.peruviana*; CRBP
*Rothschildiasantamartensis* Brechlin, 2021	+	Ce, Ma	TL; BC-RBP 10219	Brechlin 2021g; CRBP
*Rothschildiatatama* Brechlin, 2021	+	An, By, Ri, Vl	TL; BC-RBP 9578	Brechlin 2021g; ANDES-E, CRBP
*Rothschildiazacateca* (Westwood, 1854)		By, Cu, Na, Qu, To	TL; BC-RBP 8278	Westwood 1853; Amarillo-Suárez 2000; Brechlin 2022g; CRBP
Tribe Saturniini Boisduval, 1837				
Genus *Antheraea* Hübner, 1819 [1816]				
Subgenus Telea Hübner, 1819 [1816]				
Antheraea (Telea) godmani columbiana (Draudt, 1930)	§	An, By, Ca, Cu, Qu, St	TL; BC-RBP 8032	Draudt 1929; Jiménez-Bolívar et al. 2021; Amarillo-Suárez 2000 and Ramos-Artunduaga et al. 2022 as *A.godmani*; ANDES-E, CRBP
Genus *Copaxa* Walker, 1855				
*Copaxaandensis* Lemaire, 1971	+	An, Qu, Ri, Vl	TL; BC-RBP 10624	Lemaire 1971a; Amarillo-Suárez 2000; CRBP
*Copaxaandescens* Brechlin & Meister, 2012		Am		Brechlin and Meister 2012d; CRBP
*Copaxaandorientalis* Brechlin & Meister, 2012		Pu	BC-RBP 11120	Brechlin and Meister 2012d; CRBP
*Copaxaantiollita* Brechlin, 2016	+	An, By, Cu	TL; BC-RBP 10040	Brechlin et al. 2016b; ANDES-E, CRBP
*Copaxaapollinairei* Lemaire, 1978		By, Cu, St	TL; BC-RBP 8012	Lemaire 1978b; Amarillo-Suárez 2000; Decaëns et al. 2007; CRBP
*Copaxaarianae* Brechlin, Käch & Meister, 2013		Ca	BC-RBP 12777	Brechlin 2023j; CRBP
*Copaxabachuea* Wolfe, 2005	+	By, Cu, St	TL; BC-RBP 8277	Wolfe 2005; CRBP
*Copaxacabrera* Brechlin, 2016	+	Cu	TL; BC-RBP 10093	Brechlin et al. 2016b; CRBP
*Copaxacomoglioi* Brechlin, 2023	+	Vl	TL; BC-RBP 12618	Brechlin 2023j; CRBP
*Copaxadagmarae* Brechlin, Meister & van Schayck, 2016		Cl, Na, Qu, To	TL; BC-RBP 8015	Amarillo-Suárez 2000 as *C.semioculata*; Brechlin et al. 2016b; ANDES-E, CRBP
*Copaxadenhezi* Lemaire, 1971	+	Vl	TL; EL5964	Lemaire 1971a; Amarillo-Suárez 2000
*Copaxafrontina* Brechlin, 2021	+	An	TL; BC-RBP 12270	Brechlin 2021l; CRBP
*Copaxagachala* Brechlin, 2019	+	Cu	TL; BC-RBP 11117	Brechlin 2019g; CRBP
*Copaxaignescens* Lemaire, 1978, stat. rev.		Cc?, Ch, Na, Vl	TL	Lemaire 1978b; Amarillo-Suárez 2000; Muñoz and Amarillo-Suárez 2010; Brechlin et al. 2013d; CRBP
*Copaxalitensis* Wolfe & Conlan, 2002		By, Ch	BC-RBP 11314	CRBP
*Copaxaluedtkei* Brechlin, 2021		Na, Vl	BC-RBP 10705	Brechlin 2021k; ANDES-E, CRBP
*Copaxamachadoi* Brechlin, 2016	+	An, Ca, Cl, Hu	TL; BC-RBP 8650	Brechlin et al. 2016b, Brechlin 2023j; ANDES-E, CRBP
*Copaxamarquezae* Brechlin, 2016	+	By, St	TL; BC-RBP 8249	Brechlin et al. 2016b; CRBP
*Copaxametescens* Brechlin & Meister, 2016		By, Cn, Cu, Me	TL; BC-RBP 9982	Brechlin et al. 2016b; ANDES-E, CRBP
*Copaxanavalle* Brechlin, 2023	+	Na, Vl	TL; BC-RBP 12623	Brechlin 2023j; CRBP
*Copaxaniepelti* Draudt, 1929		By, Ce, Cu, Gj, Hu, Ma, St, To, Vl	TL	Draudt 1929; CRBP
*Copaxaparexpandens* Brechlin, 2016		Cu, Me, St	BC-RBP 8645	Amarillo-Suárez 2000 as *C.expandens*; Brechlin et al. 2016b; CRBP
*Copaxarufinansrufstralica* Brechlin & Meister, 2016		Ch	BC-FMP-2383	Amarillo-Suárez 2000, Decaëns et al. 2003b, and 2007 as *C.rufinans*; Brechlin et al. 2016b; CRBP
*Copaxarufotincta* Rothschild, 1895		An, Ch, Cl, Na, Ri, To, Vl	BC-RBP 9813	Amarillo-Suárez 2000, Decaëns et al. 2003b, and Muñoz and Amarillo-Suárez 2010 as *C.multifenestrata*; ANDES-E, CRBP
*Copaxasapatoza* (Westwood, 1854)	+	By, Cu, NS	TL; BC-RBP 4126	Westwood 1853; Amarillo-Suárez 2000; Wolfe et al. 2003b; ANDES-E, CRBP
*Copaxasatellita* Walker, 1865	+	By, Ce, St	TL; BC-RBP 8247	Walker 1865; Decaëns et al. 2007; CRBP
*Copaxasemioculata* (C. Felder & R. Felder, 1874)		By, Cu	BC-RBP 8024	Amarillo-Suárez 2000; Wolfe et al. 2003a; Wolfe 2005; Brechlin 2023a; CRBP
*Copaxasimoni* Brechlin, Käch & Meister, 2011		Na, To	BC-RBP 11656	Brechlin and Meister 2011b; CRBP
*Copaxasimsonsimson* Maassen & Weymer, 1881		An, By	BC-RBP 12399	Amarillo-Suárez 2000 and Decaëns et al. 2007 as *C.simson*; CRBP
*Copaxasimsonbireni* Bénéluz, 2008		Cn, Me, St	BC-Dec1450	Amarillo-Suárez 2000 as *C.simson*; CRBP
*Copaxasumacensis* Brechlin & Rimkus-Handschug, 2016		Ca, Cc, Hu	BC-RBP 11145	Brechlin et al. 2016b; CRBP
*Copaxasvetlanae* Brechlin, 2018	+	Ca, Hu	TL; BC-RBP 10970	Brechlin 2018o, 2023a; CRBP
*Copaxatroetschi* Druce, 1886		An, By, Ce, Cu, Gj, Hu, Ma, St, To, Vl	BC-RBP 3175	ANDES-E, CRBP
*Copaxaurrao* Brechlin, 2021	+	An	TL; BC-RBP 12274	Brechlin 2021l; CRBP
*Copaxavirgensis* Brechlin, 2016	+	By	TL; BC-RBP 9631	Brechlin et al. 2016b; CRBP
*Copaxawernermeisteri* Brechlin & Meister, 2010		Vl	KLWBC-078	Brechlin and Meister 2010b
*Copaxawinbrechliniani* Brechlin, 2016	+	Ma	TL; BC-RBP 8646	Brechlin et al. 2016b; CRBP
*Copaxawitti* Brechlin, Käch & Meister, 2013		Ch, Na, Vl	BC-RBP 10704	Brechlin et al. 2013d; CRBP
*Copaxayarumala* Brechlin, 2016	+	An	TL; BC-RBP 9583	Brechlin et al. 2016b; CRBP

### ﻿Discussion

We discuss the taxonomic changes proposed in this review, the current distribution of some Colombian taxa, taxa excluded from the updated checklist, and those with potential distribution in Colombia but not yet confirmed for the country. Finally, the results of the previous checklists of the Colombian Saturniidae are contrasted and discussed.

### ﻿Taxonomic changes

Racheli and Racheli (1998) described a subspecies of *Arsenurathomsoni* Schaus, 1906 that is now raised to full species status: *Arsenuralemairei* L. Racheli & T. Racheli, 1998, stat. nov. It is possible to separate this taxon from *thomsoni* by external morphology: the outer hindwing margins are smoother in *A.thomsoni* and notched in *A.lemairei*. The two species also have different distributions. Only *A.lemairei* was found in Colombia, in the eastern plains (Cn), and it was also reported in northwestern Brazil, northeastern Ecuador (Racheli and Racheli 2006), and northern Peru (type locality: Loreto). In contrast, *A.thomsoni* is a Guiano-Amazonian species distributed in the Guianas (type locality: Omai, Guyana), Venezuela, and northern Brazil (Lemaire 1980). Molecular evidence also supports this taxonomic change since there is a minimum p-distance of 4.33% between the BINs clustering *A.thomsoni* (BIN BOLD:AAC8188) and *A.lemairei* (BIN BOLD:AAC0236).

Lemaire (1980) divided *Copiopteryxsemiramis* (Cramer, 1775) into seven subspecies that range from Mexico to Bolivia. *Copiopteryxsemiramisbanghaasi* Draudt, 1930 is known from Central America (Mexico to Nicaragua) and was distinguished by Lemaire (1980: 167) as “easily recognizable by the much paler, yellowish brown, ground color than in all the previous subspecies.” Given the distribution and morphological features provided for *C.s.banghaasi*, *Copiopteryxbanghaasi* Draudt, 1930, stat. nov. is raised to full species status now. Additionally, according to these new results, the taxon *C.semiramisandensis* is found to be a subspecies of *C.banghaasi* and finally treated as *Copiopteryxbanghaasiandensis* (Lemaire, 1974), comb. nov. In summary, three taxa of the genus *Copiopteryx* are distributed in Colombia: *C.banghaasiandensis* comb. nov. is mainly found in western Colombia (An, Ch, and Vl), but also in By and St; *C.jehovah* is reported for Ca, Cn, and Pu; and *C.semiramissemiramis* is found in southeastern Colombia (Ca, Cu, and Me).

*Grammopeltalineata* (Schaus, 1906) was reported for By, Ca, Ch, Cu, and Vl (Lemaire 1980; Amarillo-Suárez 2000; Decaëns et al. 2003b, 2007), but this species should be restricted to the Guianan region only. *Grammopeltacervina* Rothschild, 1907, stat. rev. is here reinstated to species status, with its type locality in the southeastern Peruvian department of Puno (Carabaya). This species is now considered to be distributed in the Andean region, from Bolivia to Colombia. Besides biogeographic reasons, the Guianan *G.lineata* is relatively smaller than the Andean *G.cervina*, as recognized by Lemaire (1980: 185). Molecular evidence reveals the existence of three BINs, currently identified as *G.lineata* on BOLD, but here treated as follows. The BIN BOLD:AAC5835 is clustering the actual *G.lineata* from French Guiana, with a minimum p-distance of 4.86%. At the same time, the BINs BOLD:AAC5833 (Bolivia: La Paz, Peru: Madre de Dios, and Brazil: Pará) and BOLD:AAC5834 (Colombia: Boyacá and Ecuador), with a minimum p-distance of 2.26% and 2.91%, respectively, now refer to *G.cervina*, stat. rev. It must be noted that the latter BIN (BOLD:AAC5834) refers to the invalid *G.convergens* (Bouvier, 1928), which is currently a synonym of *G.cervina*. With its type locality in Colombia (Bogotá), this taxon could also be treated as a subspecies of *G.cervina*, but no taxonomic change is formally made here.

In addition, *Rhescyntisnorax* Druce, 1897, stat. nov. is removed from its subspecies status with *R.hippodamia* Druce, 1897 and now raised to full species status. The distribution of *R.norax* ranges from Mexico to western Colombia and western Ecuador. Consequently, we recognize *R.h.colombiana* Bouvier, 1927, syn. nov. as a subjective junior synonym of *R.norax*. Both species, *R.hippodamia* and *R.norax*, can be found in Colombia: *R.hippodamia* in the Amazon region (Am, Ca, and Pu) and *R.norax*, which tends to be larger, in western Colombia (Ch, Na, Ri, and Vl). Thus, the old records for Ch, Na, and Vl of *R.hippodamia* (Amarillo-Suárez 2000; Prada-Lara et al. 2019) very likely belong to *R.norax*.

*Bathyphlebiagschwandneri* Schawerda, 1925 was considered a junior subjective synonym of *B.aglia* C. Felder & R. Felder, 1874 by Lemaire (1976b). Because of external differences and features in the male genitalia *Bathyphlebiaagliagschwandneri* Schawerda, 1925, stat. nov. is here treated as a subspecies of *B.aglia*. This change mainly stems from its extreme phenotype with a broad white shadow after the black postmedian line, as figured in Naumann et al. (2009: figs 4, 5). It was also noted that “the transverse rugae of the uncus are much weaker” in *B.a.gschwandneri*, stat. nov. than in *B.a.aglia* (Oiticica Filho and Michener 1950a).

*Rothschildiaorizababogotana* Rothschild, 1907 was previously considered a synonym of *R.orizabaequatorialis* Rothschild, 1907 by Lemaire (1975). Brechlin and Meister (2012c) raised the latter to species status with its synonym *bogotana*. However, we again treat *Rothschildiaequatorialisbogotana* Rothschild, 1907, stat. rev., comb. nov. as a subspecies, but now of *R.equatorialis* with its type locality in Bogotá, Cu. The nominate subspecies is found in western Colombia, with its synonym *R.cauca* Rothschild, 1907 (Brechlin 2023l).

There are taxonomic confusions within the species group of *Copaxadescrescenssensu*Brechlin and Meister (2010b). Three species of this group were reported in western Colombia: *C.niepelti* Draudt, 1929 (type locality: West Colombia, [Valle del Cauca, Dagua], Bellavista), *C.ignescens* Lemaire, 1978 (type locality: Valle del Cauca, [Dagua, El Queremal, Cerro] Tokio), and *C.troetschi* Druce, 1886 (type locality: Panama, Chiriquí). Lemaire (1975) treated *C.niepelti* as a synonym of *C.decrescens* Walker, 1855, but the latter is restricted to Brazil. Brechlin and Meister (2012d) reinstated *C.niepelti* as a species and synonymized *C.ignescens* with the latter. In a conservative way, *Copaxaignescens* Lemaire, 1978, stat. rev. is here reinstated to species status. According to genitalia comparison and new molecular studies of material collected near the type locality of *C.ignescens*, there seems to be a great possibility that *C.witti* Brechlin, Käch & Meister, 2013 could be a synonym of *C.ignescens*. Broader distribution is recognized for *C.troetschi*, and according to preliminary DNA barcoding results, *C.niepelti* could be a synonym of *C.troetschi*. To avoid further confusion, in the checklist, this issue is treated as follows: *C.witti* is very likely a synonym of *C.ignescens* with its distribution in the Western Cordillera of Colombia (Ch, Na, and Vl) and western Ecuador; *C.niepelti* is probably a synonym of *C.troetschi*, and it is widely distributed in Colombia: By, Ce, Cu, Gj, Hu, Ma, St, To, and Vl. However, all names have been preserved, and no synonymy is here formally proposed. The old record for *C.ignescens* in Cc (Muñoz and Amarillo-Suárez 2010) could not be verified due to the cryptic diversity within this species group. The specimens identified as *C.ignescens* in the literature (Amarillo-Suárez 1997b, 2000) could not be examined and have no DNA barcodes. Further studies are necessary to clear the correct identifications and boundaries between these closely related species.

### ﻿Remarks on the checklist

In this checklist (Table 2), the distribution of each taxon is represented by the departments of Colombia where they are found, but this can be ambiguous in some cases since the departments are administrative subdivisions and not geographical units. Consequently, the presence of a given taxon in e.g., Ri may mean it occurs in the Central Cordillera and/or Western Cordillera. Caution should be exercised when extrapolating biogeographic data, as a taxon reported for example from Pu could indicate that it is Andean or Amazonian.

It should be noted that some distribution data reported in the literature are considered doubtful based on recent sampling and taxonomic advances. For example, high-altitude species are restricted to very narrow distribution ranges, while only a few lowland species are considered polytopic and can be found in both eastern and western Colombia. However, the old records could not be verified by direct examination. It must be pointed out that this could prove fruitless if only the external morphology is compared without integrating molecular evidence. The distribution of at least the following taxa, listed in alphabetic order, presents some issues that need to be discussed in depth.

*Adeloneivaiaacuta* (Schaus, 1896) was reported for Ch by Amarillo-Suárez (2000), but this species appears to be restricted to northern and eastern Colombia.

*Adelowalkeriacaeca* Lemaire, 1969 was reported for St by Amarillo-Suárez (2000), but this species is distributed in western Colombia. The old record likely refers to *A.winbrechlini*.

Antheraea (Telea) godmani columbiana (Draudt, 1930) is here considered as a valid subspecies name since it has been revived by Jiménez-Bolívar et al. (2021: 194) and thus removed from its synonymy with *A.godmani* (Druce, 1892). However, during this work, specimens from Mexico, Costa Rica, Panama, and Colombia were examined without finding significant or repeated morphological differences between the geographic populations; and neither between the barcodes.

*Arsenuraarmida* (Cramer, 1779) was reported for An, By, Ca, Cn, Cu, Ma, Me, Pu, and To departments by Amarillo-Suárez (2000) and Decaëns et al. (2007), but several new taxa were described within this species group (Brechlin and Meister 2010g). The distribution of *A.armida* is restricted to the Guianan region and was only recently confirmed by molecular evidence for Am, Ca, Cn, and Me in southern and eastern Colombia. Except for these localities, the old records of this species in Colombia must be carefully examined and assigned either to *A.archianassaarchianassa* or *A.archianassavenecolombiana*.

*Arsenurabatesii* (C. Felder & R. Felder, 1874) was reported for Vl by Amarillo-Suárez (2000), but this species is restricted to eastern Colombia. The old record should refer to *A.arcaei*.

*Arsenurabatesiiarcaei* Druce, 1886 was reported for Cu (Lemaire 1980), but this taxon has been recently raised to species status (Brechlin 2023b) and it seems to be restricted to western Colombia. The old record should refer to *A.batesii*.

*Automerisabdominalis* (C. Felder & R. Felder, 1874) was reported for An and Vl (Amarillo-Suárez 2000; Lemaire 2002), but this species is only known from the type material, whose origin was assigned to the type locality in “Colombia, Bogotá” (Brechlin 2023c). The identity of this species is doubtful, and probably among *A.abdgachala* and *A.abdsanboyacensis*, which are found in Cundinamarca (Brechlin 2023c). It must be noted that much old locality information (e.g., especially by Apollinaire Marie) are unreliable (Lemaire 2002: 897). Only genetic studies of the lectotype of *A.abdominalis* could clarify this issue (Brechlin 2023c). In light of the description of many species within this complex from western Colombia, the old records should refer to them.

*Automerisbilinea* (Walker, 1855) was reported for NS (Amarillo-Suárez 2000), but currently, there is only a barcoding evidence of this species from Cn (Jiménez-Bolívar et al. 2021). Decaëns et al. (2021) showed that this is a cryptic species complex in which the identification could be difficult.

*Automerisduchartrei* Bouvier, 1936 was reported for By (Decaëns et al. 2007), but this old record likely refers to *A.handschugi*.

*Automerisexigua* Lemaire, 1977 was reported for Cu by Amarillo-Suárez (2000), but this species is distributed in western Colombia. The old record likely refers to *A.dagmarae*.

*Automerishamata* Schaus, 1906 was reported for By, Cu, Hu, and Me (Amarillo-Suárez 2000; Decaëns et al. 2007), but this species is restricted to northern and western Colombia. The old records likely refer to *A.angulatus*.

*Automerisjanus* (Cramer, 1775) was reported for An, By, and Hu, as an Andean species, by Jiménez-Bolívar et al. (2021: 170). By contrast, in footnote 20 (Jiménez-Bolívar et al. 2021: 196), this species was mentioned for Am only (“BIN BOLD:ACF3806”). In fact, there is evidence for this species in BOLD (Sample ID: IavH-E-190356) for Am, although the available image wrongly corresponds to a specimen of *A.curvilinea*. Additionally, the old record of this species for An (Amarillo-Suárez 2000) is probably a misinterpretation of *A.exigua* or *A.dagmarae* which are both found in An.

*Automerisoccidentorestes* Brechlin & Meister, 2011 was reported for Am by Jiménez-Bolívar et al. (2021: 171), but the specimen with barcode (Sample ID in BOLD) IavH-E-190391 likely refers to *A.serpina*.

*Automerisoiticicai* Lemaire, 1966 was reported for Cc and Vl (Muñoz and Amarillo-Suárez 2010) in the western Cordillera, so this record is possible but could not be verified without barcode evidence.

*Cerodirphiamota* (Druce, 1909) was doubtfully reported for To by Lemaire (2002), but this species is probably only distributed in Vl. Thus, the old record may refer to *C.giustii*.

*Citheroniaequatorialis* Bouvier, 1927 was reported for An, Ca, St, and Vl (Lemaire 1988b; Amarillo-Suárez 2000; Racheli and Vinciguerra 2005). However, this species can be found in southwestern Colombia, at least in Na and very likely in Vl at low and medium elevations, while *C.caucensis* is found at higher elevations in Vl. The old records for An and St likely refer to *C.bellavista*.

*Copaxaandensis* Lemaire, 1971 was reported for By (Decaëns et al. 2007), but this species is restricted to more western parts of Colombia, with its type locality in Vl.

*Copaxasemioculata* (C. Felder & R. Felder, 1874) was reported for Na and To (Amarillo-Suárez 2000), but this species is restricted to the Eastern Cordillera (Wolfe 2005). The old records probably refer to *C.dagmarae* (Brechlin et al. 2016b).

*Copaxasimsonbireni* Bénéluz, 2008 was originally described in full species status from French Guiana, and later Bénéluz (2021) treated it as a subspecies of *C.simson*. At the moment, we treat *C.s.simson* (TL: Panama) for the more western populations (e.g., BC-RBP 12399 and BC-Dec0584) and *C.s.bireni* for the eastern ones (e.g., BC-Dec1450 and BC-Dec0604). Further studies are necessary.

*Copaxawernermeisteri* Brechlin & Meister, 2010 was described from Mexico (type locality: Chiapas), but this locality is erroneous. The corrected type locality is in western Colombia, Vl.

*Dirphiasomniculosa* (Cramer, 1777) was reported for Ch and Vl (Lemaire 2002; Decaëns et al. 2003b; Prada-Lara et al. 2019), but this species is restricted to eastern Colombia. Thus, the old records for western Colombia very likely belong to *D.somoccidentalis*.

*Dirphiellaniobe* (Lemaire, 1978) was reported for Na by Lemaire (2002), but the toponym of the collecting site (“Namambi”) and the location of the deposit of the cited specimen are unknown. In addition, recent sampling has not confirmed the presence of this species in the country. The distribution of this species in both the Venezuelan Cordillera de Merida and southwestern Colombian Andes is very unlikely, as well as the assignation of this species to the Mexican genus *Dirphiella*.

*Dirphiopsisflora* (Schaus, 1911) was reported for By (Decaëns et al. 2007), but this species is restricted to western Colombia. Therefore, the old record likely refers to *D.orientalis*.

*Erythromerisflexilineata* (Dognin, 1911) was reported for By by Lemaire (2002). However, this species is only confirmed at its type locality, Paramo del Quindío, and further south in Vl. Thus, this old record likely refers to *E.christbrechlinae*.

*Gameliakiefferi* Lemaire, 1967 was reported for Cc (Muñoz and Amarillo-Suárez 2010) and Cu (Amarillo-Suárez 2000), but this species is only found near its type locality in Anchicayá, Vl. The old record for Cc is possible but could not be verified recently.

*Gameliapyrrhomelas* (Walker, 1855) was reported for Na and Vl (Amarillo-Suárez 2000; Lemaire 2002), but this species is only known to us from its type locality near Bogotá, Cu.

*Gamelioidessachai* Brechlin, Käch & Meister, 2011 is probably a synonym of *G.elainae* (Lemaire, 1967), according to current studies of this genus (Brechlin 2016e, 2018n), but the DNA study of the female holotype of the latter is needed.

*Hirpidagaujoni* (Dognin, 1894) was reported for By by Lemaire (2002), but this old record very likely refers to *H.peggyae*.

Hylesia (Hylesia) mymex Dyar, 1913 was reported for By (Decaëns et al. 2007), but this species is restricted to western Colombia. The old record likely refers to H. (H.) mymsantandex.

Hylesia (Hylesia) olivenca Schaus, 1927 was reported for Ch (Decaëns et al. 2003b), but this species is restricted to eastern Colombia.

Hylesia (Hylesia) praeda Dognin, 1901 was reported for An, Ch, and Vl (Amarillo-Suárez 2000; Lemaire 2002), but this species is restricted to eastern Colombia. The old records likely refer to H. (H.) praedpichinchensis.

*Leucanellaflammans* (Schaus, 1900) was reported for Me by Amarillo-Suárez (2000), but this species is restricted to western Colombia.

*Leucanellanyctimene* (Latreille, 1832) was reported for Cc, Cu, Na, and Ri (Amarillo-Suárez 2000; Lemaire 2002). According to recent studies, including the description of several new taxa within this species complex, *L.nyctimene* is only found in Cu until now (Brechlin 2021c). Thus, the old records of this species for Cc and Ri probably belong to *L.tolimaiana*, while the record for Na probably refers to *L.bolanosi*.

*Lonomiacolumbiana* Lemaire, 1972 was reported for By and Ma (Amarillo-Suárez 2000; Lemaire 2002), but this species is restricted to western Colombia.

*Lonomiarufescens* Lemaire, 1972 was reported for By (Decaëns et al. 2007), but this species is restricted to western Colombia.

Meroleuca (Meroleucoides) flavodiscata (Dognin, 1916) was reported for Cl and Cu (Lemaire 2002). However, species of this genus are known to have very narrow distributions. Thus, the old records likely refer to other species of this genus.

*Molippalatemedia* (Druce, 1890) was reported for An by Amarillo-Suárez (2000), but this species is restricted to eastern Colombia. The old record likely refers to *M.latcolombiana*.

*Othorenepurpurascens* (Schaus, 1905) was reported for Ch, Na, and Vl (Amarillo-Suárez 2000; Decaëns et al. 2003b; Prada-Lara et al. 2019), but this species is a Guiano-Amazonian species that is found in eastern Colombia. The old records likely belong to *O.vanschayckorum*.

*Paradaemoniaplatydesmia* (Rothschild, 1907) was reported for Ch and Vl by Amarillo-Suárez (2000), but these old records likely refer to *P.castanea*.

*Perigaoccidentalis* (Lemaire, 1972) was reported for By and St (Lemaire 2002), but this species is restricted to western Colombia. Additionally, *Perigaelsa* and *P.intensiva* could be synonyms of *P.occidentalis*, as discussed by Brechlin (2023k), but no taxonomic change was made herein.

*Pseudautomerisantioquia* (Schaus, 1921) was reported for Ch, Na, and Vl (Amarillo-Suárez 2000; Lemaire 2002; Decaëns et al. 2003b), but this species is endemic to An. The old, more southern records likely refer to *P.winbrechlini*.

*Pseudodirphiainfuscata* (Bouvier, 1924) was reported for An, Cl, Cu, and Me (type locality) (Amarillo-Suárez 2000; Lemaire 2002), but the identity and distribution of this species are doubtful and need further studies. This species’ type locality does not refer to the collecting site, as explained by Lemaire (2002: 897).

*Rothschildiaaricia* (Walker, 1855) was reported for Na and Vl (Amarillo-Suárez 2000). These old records belong to the subspecies *Rothschildiaa.napoecuadoriana*.

### ﻿Excluded taxa

The following 94 taxa, listed in alphabetic order, were previously reported for Colombia but are excluded from the current checklist due to recent changes in taxonomy and the descriptions of new species, as well as new findings regarding the distribution ranges of several known taxa.

*Adeloneivaiajason* (Boisduval, 1872) was reported for Ca, Cc, Ch, Me, and Vl (Amarillo-Suárez 2000; Decaëns et al. 2003b; Racheli and Vinciguerra 2005; Muñoz and Amarillo-Suárez 2010), but this is a complex of species, and *A.jason* is known to be restricted to Mexico and northern Guatemala (Brechlin 2017b). Several new species of this species complex have been described, so the old records likely refer to them.

*Adeloneivaiasubangulata* (Herrich-Schäffer, 1855) was reported for An, Ca, Ch, Me, and Vl (Lemaire 1988b; Amarillo-Suárez 2000; Racheli and Vinciguerra 2005). However, the old records likely refer to *A.pallida*, previously considered as a subspecies of *A.subangulata* and later raised to full species status by Brechlin and Meister (2011c).

*Adelowalkeriaeugenia* (Druce, 1904) was reported for Hu (Lemaire 1988b; Amarillo-Suárez 2000), but this species is restricted to the Guianan region. Therefore, the old record likely refers to either *A.bezverkhovi*, which is still not confirmed for Colombia, or *A.eugenicolombiana* (Brechlin and Meister 2011c; Brechlin 2017m).

*Adelowalkeriaplateada* (Schaus, 1905) was reported for Ca (Racheli and Vinciguerra 2005), but this species is restricted to the Guianan region. Therefore, the old record likely refers to *A.witti* (Brechlin and Meister 2011c).

*Automerisamanda* Schaus, 1900 was reported for Cu (Amarillo-Suárez 2000), but the nominate subspecies *A.a.amanda* is known only from southern Peru and Bolivia. The old record likely refers to *A.subobscura* distributed in the Eastern Cordillera of Colombia at moderate elevations nearby its type locality near Bogotá, Cu (Lemaire 2002).

*Automerisbanus* (Boisduval, 1875) was reported for Ch, Na, and Vl (Amarillo-Suárez 2000), but this species ranges from Mexico to Costa Rica (Brechlin and Meister 2011f). The old records likely refer to *A.argentifera*, raised to full species status by Brechlin and Meister (2011f).

*Automerisbanusproxima* Conte, 1906 was reported for By (Decaëns et al. 2007). However, this record was initially stated as doubtful by the authors. This subspecies, only known from southwestern Ecuador (Racheli and Racheli 2005), is probably a synonym of *A.argentifera*, to which the old record likely refers.

*Automerisbelti* Druce, 1886 was reported for Ch (Prada-Lara et al. 2019), but this species is known from Nicaragua (type locality), Honduras, Costa Rica, and northwestern Panama. The old record likely refers to *A.zaruma*, formerly treated as a subspecies of *A.belti*, but previously raised to full species status by Brechlin and Meister (2011f).

*Automeriscelata* Lemaire, 1969 was reported for Ch (Decaëns et al. 2003b). However, several new taxa were recently described within this species complex (Brechlin and Meister 2011f). The distribution of *A.celata* sensu stricto is now known to be restricted to Costa Rica. The old record likely refers to *A.choco*.

*Automerisjivaros* Dognin, 1890 was reported for Hu in our unreviewed preprint (Comoglio and Brechlin 2021) and later also cited by Jiménez-Bolívar et al. (2021: 171), but the given evidence (BC-RBP 11131) refers to *A.harriamazonica*.

*Automerislapaza* Brechlin & Meister, 2017 was reported for Me by Jiménez-Bolívar et al. (2021: 171), but this species is only known from the Bolivian department of La Paz. This wrong record is based on a mislabeled specimen (Sample ID [in BOLD]: “BC-FMP-0652”). In the original description of this species by Brechlin et al. (2017), this specimen is listed as a paratype with the correct locality data.

*Automerislarra* (Walker, 1855) was reported from Am by Jiménez-Bolívar et al. (2021: 171), but this species is restricted to southeastern Brazil. In fact, the record with barcode IAvH-E-190358 corresponds to a specimen of *A.mixtus*.

*Automerismetzli* (Sallé, 1853) was reported for By and Vl (Amarillo-Suárez 2000; Lemaire 2002; Decaëns et al. 2007), but this species is now known to be restricted to Mexico. Thus, the old records likely refer to *A.dagmarae* or *A.exigua* (Brechlin and Meister 2011f).

*Automerismidea* (Maassen, 1885) was reported for By (Decaëns et al. 2007), but this species has a more eastern distribution, restricted to Brazil: Pará and the Guianan region (Decaëns et al. 2021). The old record likely refers to *A.mineros*, recently described from the same area.

*Automerismoresca* Schaus, 1906 was reported from Ca by Jiménez-Bolívar et al. (2021: 171), but this species is restricted to the Guianan region. The old record, which is missing barcode evidence, likely refers to *A.fabiani*.

*Automeriszugana* Druce, 1886 was reported for An, By, Ch, and Vl (Amarillo-Suárez 2000; Decaëns et al. 2003b, 2007; Prada-Lara et al. 2019), but this species is only known from Costa Rica and Panama. The old records of this species likely refer to *A.parapichinchensis*.

*Cerodirphiaaraguensis* Lemaire, 1971 was reported for Colombia by Jiménez-Bolívar et al. (2021: 173), but this record is very likely based on a mislabeled and/or misidentified specimen (Sample ID [in BOLD]: “BC-EvS 1496”). It is indeed a specimen of *C.brunnea*, whose distribution is discussed below. Nevertheless, the occurrence of *C.araguensis* in eastern Colombia cannot be completely excluded, but this seems to be quite unlikely because of biogeographical reasons.

*Cerodirphiabrunnea* (Draudt, 1930) was reported for Ca by Racheli and Vinciguerra (2005), but these authors already had some doubts about the identification between *C.brunnea* and *C.speciosa*. However, the latter can be easily distinguished by its pink color and smaller size. *Cerodirphiabrunnea* was also reported for Colombia, within the first checklist of the Colombian Saturniidae by Amarillo-Suárez (2000), but without specifying its distribution. According to Lemaire (2002), this species ranges from Ecuador to Bolivia. However, *C.brunnea* seems to be restricted to Argentina and Bolivia. Thus, the old record could refer to *C.siriae*, which is externally closer to *C.speciosa* (Brechlin 2011).

*Cerodirphiasanctimartinensis* Lemaire, 1982 was reported for Ma (Amarillo-Suárez 2000), but this species seems to be endemic to northern Peru (Lemaire 2002). Thus, the old record likely refers to another species of this genus or even an undescribed taxon, given that confusing *C.sanctimartinensis* with any other species of the genus is very unlikely.

*Cerodirphiaspeciosa* (Cramer, 1777) was reported for Ca (Lemaire 2002; Racheli and Vinciguerra 2005), but this species is restricted to the Guianan region. The old record likely refers to *C.siriae* (Brechlin 2011).

*Ciciapelota* (Schaus, 1905) was reported for Ca (Racheli and Vinciguerra 2005), but this species is restricted to the Guianan region. The old record should refer to *C.pelotandana*, distributed in the Andean region (Brechlin 2023e).

*Citheroniahamifera* Rothschild, 1907 was reported for Ca (Racheli and Vinciguerra 2005), but it is now known to be restricted to Trinidad and probably northern Venezuela and French Guiana. Therefore, the old record likely refers to *C.witti* (Brechlin et al. 2019a).

*Citheronialaocoon* (Cramer, 1777) was reported for Cu by Racheli and Racheli (2006), who examined specimens in the collection of Lemaire, C. (in MHNH, Paris). According to Brechlin et al. (2019a) and the available COI barcodes (in BOLD), this species is distributed from northeastern to southern Brazil, eastern Argentina (Misiones province), and Paraguay; but it is not an Andean species at all, as referred to by Jiménez-Bolívar et al. (2021: 163). Consequently, the old record likely refers to *C.laocandensis*.

*Citheronialobesis* Rothschild, 1907 was reported for An, Cu, Hu, and To (Lemaire 1988b; Amarillo-Suárez 2000), but two species were recently described within this species complex. Given that *C.lobesis* is only known from Mexico (Brechlin et al. 2019a), the old records likely refer to *C.laguajira*.

*Citheroniaphoronea* (Cramer, 1779) was reported for An, Ca, Ch, Me, and Vl (Amarillo-Suárez 2000; Decaëns et al. 2003b; Racheli and Vinciguerra 2005), but several species were described within the *phoronea*-species complex (Brechlin et al. 2019a). *Citheroniaphoronea* sensu stricto is known to be restricted to the Guianan region, including parts of eastern Venezuela. Thus, the old records likely refer to *C.phochocoensis* in western Colombia (An, Ch, and Vl) or *C.phoandensis* in eastern Colombia (Ca and Me).

*Citioicaanthonilis* (Herrich-Schäffer, 1854) was reported for By, Ca, Ch, Me, and Vl (Amarillo-Suárez 2000; Decaëns et al. 2003b, 2007; Racheli and Vinciguerra 2005; Jiménez-Bolívar et al. 2021), but the type locality of this species is in (southeastern?) Brazil. Therefore, the old records in Colombia likely refer to *C.colombiana* or *C.rubrocanescens* (Brechlin 2017c). Furthermore, the latter could also be treated as a subspecies of *C.anthonilis*, given the current molecular evidence, but no formal taxonomic change is made here.

*Citioicahomoea* (Rothschild, 1854) was reported for Me (Amarillo-Suárez 2000), but this species has a more southern distribution. In Colombia, the old record likely refers to *C.kaechi* (Brechlin 2017c).

*Copaxadecrescens* Walker, 1855 was reported for An, By, Ch, Me, and Na (Amarillo-Suárez 2000; Decaëns et al. 2003b, 2007), but this species is restricted to southeastern Brazil (Brechlin and Meister 2012d). The old records likely refer to either *C.andescens* or *C.metescens*.

*Copaxaexpandens* Walker, 1855 was reported for St (Amarillo-Suárez 2000), but this species is known to be restricted to northern Venezuela (Aragua and Carabobo) (Brechlin et al. 2016b). The old record likely refers to *C.parexpandens*.

*Copaxamultifenestrata* (Herrich-Schäffer, 1858) was reported for By, Cc, Ch, Na, St, and To (Amarillo-Suárez 2000; Decaëns et al. 2003b; Muñoz and Amarillo-Suárez 2010). However, this species is known to be restricted to Mexico only (Brechlin and Meister 2012d). The old records of this species could refer to e.g., *C.rufotincta*.

*Copaxarufinans* Schaus, 1906 was reported for An, By, Ch, and Vl (Amarillo-Suárez 2000; Decaëns et al. 2003b, 2007). However, the nominate subspecies is restricted to Mexico only. The old record for Ch very likely refers to *C.r.rufstralica*.

*Dirphiaavia* (Stoll, 1780) was reported for An, Ch, Cn and Me (Amarillo-Suárez 2000; Decaëns et al. 2003b; Jiménez-Bolívar et al. 2021), but several taxa were newly described or reinstated within this species complex (Brechlin and Meister 2011d; Brechlin 2017j). The distribution of *D.avia* sensu stricto is restricted to the Guianan region. The old records likely refer to *D.aviluisiana* for An, *D.avichoco* for Ch (Brechlin and Meister 2011d), and *D.concolor* for Cn and Me (Brechlin 2022k).

*Dirphiacrassifurca* Lemaire, 1993 was reported for An, By, Cl, and St (Amarillo-Suárez 2000; Lemaire 2002; Decaëns et al. 2007), but it is now known to be restricted to Venezuela. This species is part of a complex of species. The old records of this species should mainly refer to *D.crassgachala*, but other possibilities are *D.santboyacensis*, *D.tolimafurca*, or *D.yarumala*.

*Dirphiaradiata* Dognin, 1916 was reported for Colombia by Jiménez-Bolívar et al. (2021: 174), but until now this species is known from French Guiana only. The specimen with barcode (Sample ID in BOLD) PCG19, became a paratype of the recently described *D.radinirida* from Gn (Brechlin and Comoglio 2023d).

*Dysdaemoniaboreas* (Cramer, 1775) was reported for An, Ar, By, Ch, and Vl by Amarillo-Suárez (2000), Decaëns et al. (2003b), and Decaëns et al. (2007). However, according to Brechlin (2019e), this is a complex of species. *Dysdaemoniaboreas* is restricted to the Guianan region, including parts of eastern Venezuela and northern Brazil (Brechlin 2019e). The old records of this species in Colombia likely refer to *D.australoboreas*, *D.panamana*, or *D.vanschaycki*.

*Eaclesadoxa* Jordan, 1910 was reported for Ca (Amarillo-Suárez 2000; Racheli and Vinciguerra 2005), but this species is restricted to the Guianan region (Brechlin 2022i). The old record likely refers to the recently described *E.adoxandensis*.

*Eaclesfulvaster* Rothschild, 1907 was reported for Ca (Racheli and Vinciguerra 2005) as *Eaclesmasonifulvaster*, but later raised to full species status by Brechlin and Meister (2011c). Given that *Eaclesf.fulvaster* has a more southern distribution, the old record of this taxon likely refers to *Eaclesf.oriecuadoriana* (Brechlin and Meister 2011c).

*Eaclesimperialis* (Drury, 1773) was reported for An, Cc, Ch, Cu, Ma, Na, To, and Vl (Amarillo-Suárez 2000; Muñoz and Amarillo-Suárez 2010), but this is a North American species. The old records likely refer to either *E.anchicayensis* or *E.impandensis*.

*Eaclesimperialisanchicayensis* Lemaire, 1971 was reported for Ch (Decaëns et al. 2003b; Prada Lara et al. 2019), but this subspecies has been raised to full species status (Brechlin 2022i).

*Eaclesimperialiscacicus* (Boisduval, 1868) was reported for By and Ca (Racheli and Vinciguerra 2005; Decaëns et al. 2007), but this Brazilian taxon has been synonymized with *E.magnifica* Walker, 1855 (Brechlin 2022i). The old records likely refer to *E.anchicayensis* for By and *E.impandensis* for Ca.

*Eaclesmasoni* Schaus, 1896 was reported for Ch and Vl (Amarillo-Suárez 2000), but this is another complex of taxa. In the *masoni*-species group sensu Brechlin and Meister (2011c), the distribution of *E.masoni* sensu stricto is restricted to Mexico and northern Guatemala only. Thus, the old records of *masoni* likely refer to *E.tyrannus*. The latter has been raised to full species status from its previous subspecies status with *E.masoni* by Brechlin and Meister (2011c).

*Eaclesormondei* Schaus, 1889 was reported for Cc (Muñoz and Amarillo-Suárez 2010), but *E.ormondei* sensu stricto is only known from Mexico. Thus, the old record could refer to *E.niepelti* or *E.violacea*. Both taxa have been recently released from their subspecies status of *E.ormondei* by Brechlin (2022i).

*Eaclesormondeiniepelti* Draudt, 1930 was reported for Ch, Na, and Vl (Amarillo-Suárez 2000; Decaëns et al. 2003b; Prada Lara et al. 2019), but this subspecies has been raised to full species status by Brechlin (2022i).

*Gameliaabasia* (Stoll, 1781) was reported for Ar, Ch, Cu, and Vl (Amarillo-Suárez 2000; Lemaire 2002; Decaëns et al. 2003b), but this is another complex of species. *Gameliaabasia* is restricted to the Guianan region (Brechlin and Meister 2012b). The old records for Ch likely refer to *G.cimarrones* (Decaëns et al. 2005), while for other departments, to other species of this genus (Brechlin and Meister 2012b; Brechlin 2018k, 2020f, 2021d).

*Gamelianeidhoeferi* Lemaire, 1967 was reported for Cu, Ri, and To (Amarillo-Suárez 2000; Lemaire 2002), but this species has a more southern distribution (type locality: Bolivia, Cochabamba). Thus, the old records likely refer to several other species, such as *G.cundboyacensis* for Cu or *G.ristolima* for Ri and To.

*Homoeopteryxmajor* Jordan, 1924 was reported for Ch (Decaëns et al. 2003b), but this species is probably only distributed in southern Peru due to its type locality in Puno department.

Hylesia (Hylesia) aeneides aeneides (Druce, 1897) was reported for By, Na, and Vl (Amarillo-Suárez 2000; Lemaire 2002; Decaëns et al. 2007), but the nominate subspecies has not been found in Colombia until now. The old records for Na and Vl likely refer to *H.a.aenocciecuadorex* and *H.aencocornex* for By.

Hylesia (Hylesia) andensis (Druce, 1897) was reported for By and Hu in our unreviewed preprint (Comoglio and Brechlin 2021) and also cited in Jiménez-Bolívar et al. (2021: 178) but the evidence (BC-RBP 9879) refers to *H.andecuadorex*.

Hylesia (Hylesia) beneluzi Lemaire, 1988 was reported for Ch (Decaëns et al. 2003b). However, this record was initially stated as doubtful by the authors, as this species seems to be endemic to Costa Rica.

Hylesia (Hylesia) canitia (Cramer, 1780) was reported for St (Lemaire 2002), but this species is now known to be restricted to the Guianan region. The old record likely refers to any other taxon of this species group.

Hylesia (Hylesia) coex Dyar, 1913 was reported for Cc (Amarillo-Suárez 2000; Lemaire 2002), given that this is the type locality of *H.caucanex*, which was considered as a synonym by Lemaire (2002) but was later reinstated to species status (Brechlin et al. 2016a). According to Brechlin et al. (2016a), this species should occur in Venezuela only.

Hylesia (Hylesia) continua (Walker, 1865) was reported for An and Cc (Amarillo-Suárez 2000; Muñoz and Amarillo-Suárez 2010), but *H.C.continua* is a Central American subspecies. The old records likely refer to *H.c.colombiana*.

Hylesia (Hylesia) gyrex Dyar, 1913 was reported for Me (Lemaire 2002; Jiménez-Bolívar et al. 2021), but this species is restricted to the Guianan region. The old record likely refers to *H.gyramazonex*.

Hylesia (Hylesia) rosacea Schaus, 1911 was reported for Ch (Amarillo-Suárez 2000; Prada-Lara et al. 2019), but *H.r.rosacea* is a Central American subspecies. The old record likely refers to *H.r.thaumex*, which is proofed to occur in Colombia.

*Hyperchiriaacuta* (Conte, 1906) was reported for Vl (Lemaire 2002; Jiménez-Bolívar et al. 2021), but this species seems to have a more southern distribution, given its type locality in Peru. The old record likely refers to *H.parallela* or *H.volcana*, both distributed in western Colombia and distinguishable by size, as the latter is much bigger than the former.

*Hyperchirianausica* (Cramer, 1779) was reported for An, By, Ca, Ch, and Na (Amarillo-Suárez 2000; Decaëns et al. 2003b, 2007; Racheli and Vinciguerra 2005), but this is a complex of species. *Hyperchirianausica* is now known to be restricted to the Guianan region. Many species have been described within this species group, so the old records likely refer to them (Brechlin and Meister 2010c; Brechlin et al. 2011b; Brechlin 2019f).

*Janiodeslaverna* (Druce, 1890) was reported for Ch (Decaëns et al. 2003b), but this is another complex of species. *Janiodeslaverna* sensu stricto is restricted to western Ecuador only (Brechlin 2020e). Several new taxa within this species group have been described in a recent revision (Brechlin 2020e), so the old record likely refers to one of them.

“*Janiodespraeclara* Naumann et al.” is here considered as a nomen nudum, as it has not been validly described. This name was only mentioned once in Decaëns et al. (2003b) as a species to be described from Ch.

*Leucanellayungasensis* Meister & Naumann, 2006 was reported for Cu by Jiménez-Bolívar et al. (2021: 183), but this species is only known from southern Peru, Bolivia, and Argentina. The old record is probably based on a mislabeled specimen (Sample ID [in BOLD]: “Bc-Roug0012”). Its origin is very likely from Santa Cruz department in Bolivia, compared with other barcoded specimens from the same area.

*Lonomiaachelous* (Cramer, 1777) was reported for Cn by Jiménez-Bolívar et al. (2021: 183), but this species is restricted to the Guianan and Amazon region (Brechlin and Meister 2019; Bénéluz 2021). Lemaire (1973) designated a neotype of *L.achelous* from Surinam. Furthermore, Brechlin and Meister (2019: 16) discussed the misinterpretation of this taxon in Lemaire (2002). The old record for Cn should refer to *L.casanarensis*. Further studies and barcoding of the neotype are needed to clarify the identity of *L.achelous*.

Meroleuca (Meroleucoides) diazmaurini Decaëns, Bonilla & Ramirez, 2005 was reported for Cl (Decaëns et al. 2004b), but this species has been synonymized with *M.fassli* (Brechlin 2018i).

Meroleuca (Meroleucoides) erythropus (Maassen, 1890) was reported for To (Amarillo-Suárez 2000), but this species is currently only known from Ecuador (Lemaire 2002). The old record likely refers to another species of this genus. However, this species could be expected in southern Colombia, as it occurs in the north-Ecuadorian province of Carchi, very near the border with Na.

*Molippanibasa* Maassen & Weyding, 1885 was reported for By and Ch (Decaëns et al. 2003b, 2007; Prada-Lara et al. 2019), but this species is restricted to Mexico (neotype designation in Brechlin 2021a). The old records of this species likely refer to *M.flavotegana* (Brechlin and Meister 2011a).

*Molippasimillima* Jones, 1907 was reported for By, Ca, Ch, Cn, Cu, Hu, Me, and Pu (Jiménez-Bolívar et al. 2021), but this species is distributed in Brazil and northeastern Argentina. The old records likely refer to *M.simandensis*, *M.basina*, or *M.flavotegana*.

*Othorenehodeva* (Druce, 1904) was reported for Ca (Racheli and Vinciguerra 2005), but this species is now known to be restricted to the Guianan region. The old record likely refers to *O.winbrechlini* (Brechlin and Meister 2011j).

*Oxytenisalbilunulata* Schaus, 1912 was reported for Ch (Decaëns et al. 2003b), but the nominate *O.a.albilunulata* is a Central American taxon. The old record likely refers to *O.a.albecuatoriana*, which is known to occur in western Colombia.

*Oxytenisleda* Druce, 1906 was reported for Ca (Racheli and Vinciguerra 2005), but this species is restricted to central Peru. The old record could refer to *O.panguana*.

*Oxytenisnaemiaorecta* Jordan, 1924 was reported for Ch (Decaëns et al. 2003b; Jiménez-Bolívar et al. 2021), but this subspecies is known from Costa Rica only. The old record likely refers to *O.naemiajordani*.

*Paradaemoniaandensis* (Rothschild, 1907) was reported for Me by Amarillo-Suárez (2000), but this species ranges from central Peru to Bolivia (Brechlin 2018b). The old record could refer to *P.platydesmia*.

*Paradirphiaandicola* Lemaire, 2002 was reported for Cu in the original description. However, this species is known to be very likely restricted to eastern Ecuador only. The old record could refer to e.g., *P.cabrera* (Brechlin and Meister 2017) or *P.cundala* (Brechlin 2022a).

*Paradirphiaapollinairei* (Bouvier, 1930) was reported for eastern Colombia by Lemaire (2002), but according to Brechlin and Meister (2017), this species is not valid. The old record of this species likely refers to e.g., *P.cabrera* or *P.santander*.

*Paradirphiageneforti* (Bouvier, 1923) was reported for Cc and Na (Amarillo-Suárez 2000), but this species is known to be endemic to Ecuador (Imbabura and Pichincha) (Lemaire 2002). The old records likely refer to *P.gencarchensis* that has been recently described from northern Ecuador (Carchi).

*Paradirphiaoblita* Lemaire, 1976 was reported for By (Decaëns et al. 2007), but the occurrence of this species is not confirmed for the Eastern Cordillera in Colombia. However, it could be expected in the Colombian Amazon as it was found in the Ecuadorian Amazon (Napo and Pastaza provinces). The old record of this species likely refers to *P.cavichensis*.

*Paradirphiatorva* (Weymer, 1907) is a *species inquirenda*, according to Lemaire (2002), given that the holotype is lost. Furthermore, its Colombian origin is doubtful as there is no information apart from the original description, and there is no illustration either. Consequently, it was excluded from our checklist.

*Perigaangulosa* (Lemaire, 1972) was reported for Ca (Racheli and Vinciguerra 2005), but this is another complex of species (Brechlin and Meister 2013d). *Perigaangulosa* sensu stricto is only known in Ecuador until now. The old record likely refers to *P.angcaucana* (Brechlin 2021f).

*Perigabispinosa* (Lemaire, 1972) was reported for Ca by Jiménez-Bolívar et al. (2021: 187), but this species seems to have a more southern distribution, given its type locality in Peru (Huánuco), and is only known from Ecuador, Peru, and Bolivia. The old record likely refers to *P.sanmartiniana*.

*Perigacluacina* (Druce, 1886) was reported for Vl (Amarillo-Suárez 2000). However, this species is known to occur in Costa Rica and Panama only. The old record likely refers to e.g., *P.kaechi* or *P.pachijalensis*.

*Periphobaarcaei* (Druce, 1886) was reported for An and By (Amarillo-Suárez 2000; Decaëns et al. 2007), but this is a Central American species (Brechlin and Meister 2010d). The old records of this species likely refer to *P.tolimaiana*.

*Periphobahircia* (Cramer, 1775) was reported for Me (Amarillo-Suárez 2000), but this species is recently known to be restricted to the Guianan region (Brechlin et al. 2019b). The old record likely refers to *P.huaticocha*.

*Pseudautomerisirene* (Cramer, 1779) was reported for Ch (Decaëns et al. 2003b), but this species is now known to be restricted to the Guianan region (Brechlin and Meister 2010e). The old record likely refers to *P.chocensis* (Brechlin et al. 2013f).

*Pseudodirphiaagis* (Cramer, 1775) was reported for An, By, Ca, Cu, Ma, Me, and St (Amarillo-Suárez 2000; Decaëns et al. 2007), but this species is now known to be restricted to the Guianan region (Brechlin and Meister 2011g). The old records likely refer to *P.sinuosa*.

*Pseudodirphiaconvexa* Bouvier, 1929 was reported for To by Jiménez-Bolívar et al. (2021: 189), but this species has been recently treated as a synonym of *P.pallida* by Brechlin (2021b).

*Pseudodirphiaeumedide* (Stoll, 1782) was reported for Ca, Ch, and Cu (Amarillo-Suárez 2000; Lemaire 2002; Racheli and Vinciguerra 2005), but this species is now known to be restricted to the Guianan region (Brechlin 2018j). Therefore, the old records likely refer to e.g., *P.concava*, *P.ecandides*, *P.ecoccidides*, or *P.septentrides*.

*Pseudodirphiaeumedidoides* (Vuillot, 1893) was reported for Cu and Me (Amarillo-Suárez 2000), but this is a Brazilian species and its species group needs further research. There are several possibilities to which the old records could refer to e.g., *P.concava*, *P.ecandides*, *P.ecoccidides*, or *P.septentrides*.

*Pseudodirphiamenandermenander* (Druce, 1886) was reported for Cc, Ch, and Vl (Amarillo-Suárez 2000; Decaëns et al. 2003b; Muñoz and Amarillo-Suárez 2010), but this nominate subspecies is known to be distributed in Central America only. The old records of this subspecies likely refer to *P.m.reducta* that occurs in western Colombia.

*Pseudodirphiaperuviana* (Bouvier, 1924) was reported for Cu (Lemaire 2002) and St (Amarillo-Suárez 2000), but this species seems to have a more southern distribution, given its type locality in the Peruvian department of Puno. According to Lemaire (2002: 896), the identification of the specimen collected by Fassl in Cu was doubtful, and he noted that “several species may be involved, the separation of which however remains problematic.” Given the recent description of several new species of this genus from Colombia (Brechlin and Meister 2011g; Brechlin 2018j), the old records of *P.peruviana* for Colombia should refer to another species of this genus that is distributed in the Eastern Cordillera, despite its occurrence in the Colombian Amazon (e.g., Am and Pu) cannot be excluded.

Psilopygida (Psigida) walkeri (Grote, 1867) was reported for Me (Amarillo-Suárez 2000), but this species has a much more eastern as well as southern distribution in South America. The old record likely refers to *P.apollinairei*, which has been raised to full species status by Brechlin and Meister (2011c).

*Ptiloscolaphotophila* (Rothschild, 1907) was reported for Am and Ca (Amarillo-Suárez 2000; Racheli and Vinciguerra 2005), but this species is known to be restricted to the Guianan region. The old records likely refer to *P.wolfei* (Brechlin and Meister 2008).

*Rachesabreteuili* (Bouvier, 1927) was reported for Vl (Amarillo-Suárez 2000), but the nominate *Rachesab.breteuili* is only known from Ecuador until now. Thus, the old record likely refers to the subspecies *Rachesab.caucensis* which is distributed in western Colombia (Brechlin 2017k).

*Rothschildiaaurota* (Cramer, 1775) was reported for Me (Amarillo-Suárez 2000), but the nominate *R.a.aurota* is recently known to be restricted to the Guianan region (Brechlin and Meister 2013e). The old record likely refers to *R.a.auroamazonensis*.

*Rothschildiaincainca* Rothschild, 1907 was reported for An, By, Ch, Me, St, and Vl (Amarillo-Suárez 2000; Decaëns et al. 2003b, 2007), formerly as *R.lebeauinca*, that recently was raised to species status (Brechlin and Meister 2012c). The subspecies *R.i.inca* ranges from southern Peru to northern Bolivia. Thus, the old records for By, Me, and St likely refer to *R.i.inccolombiana* and An, Ch, and Vl to *R.lebeauaroma*.

*Rothschildiaorizaba* (Westwood, 1854) was reported for Cc, Ch, and Na (Amarillo-Suárez 2000; Muñoz and Amarillo-Suárez 2010), but this taxon is restricted to Central America, from Mexico (*R.o.orizaba*) to Costa Rica (*R.o.verapaziana* Brechlin & Meister, 2012). Thus, the old records likely refer to *R.equatorialis* which has been raised to full species status by Brechlin and Meister (2012c).

*Rothschildiaperuviana* Rothschild, 1907 was reported for Colombia (Jiménez-Bolívar et al. 2021), but the distribution of the nominotypical species seems to be restricted to southern Peru and northern Bolivia only. The old record likely refers to its subspecies *R.peruvianacoxeyi*.

*Theriniatransversariatransversaria* (Druce, 1887) was described from Nicaragua, Costa Rica, Panama, and Colombia, with its type locality (due to Jordan 1924) in Panama, Chiriquí. Jordan (1924) described two subspecies of *T. transversaria: salax* from Nicaragua and Costa Rica (type locality: Carreblanco [*sic* = Cariblanco, Alajuela]) and *columbiana* from Colombia, Muzo [Boyacá]. Given the similarity in the morphology of the male genitalia and the current molecular evidence (Brechlin and Meister 2014b), *T.t.salax* and *T.t.columbiana* should be synonymized (both being clustered into the BIN BOLD:AAB5377) and they are different from the nominate *T.t.transversaria* (clustered into the BIN BOLD:ABX5137, with a minimum p-distance of 2.56% from the former BIN), but no taxonomic change is made here. In order to avoid confusion, the old records of *T.t.transversaria* for Ch, Ma, and Vl reported by Jiménez-Bolívar et al. (2021: 192) should refer to *T.t.columbiana*, since the Panamanian nominotypical species cannot be confirmed for Colombia.

### ﻿Unconfirmed taxa to be expected in Colombia

Some regions of Colombia are undersampled since there is surely a sampling bias that favored collecting in the Andes (Correa-Carmona et al. 2015). As a result, no records are known for the following Colombian departments: Atlántico, Bolívar, Córdoba, San Andrés y Providencia, and Sucre; and few samplings have been carried out in Guainía, Guaviare, Vaupés, and Vichada. Future studies and sampling in the Caribbean and Amazon regions should reveal additional species which should be expected in Colombia, as they have been reported in neighboring countries already. The following 16 taxa can be expected for the Colombian fauna due to their known distribution close to the Colombian border, but their occurrence in Colombia is not yet confirmed.

*Adelowalkeriabezverkhovi* Brechlin, 2017 was described from specimens collected in Venezuela (Mérida) and Ecuador (Orellana) (Brechlin 2017m). Therefore, its occurrence should be expected in eastern Colombia.

*Arsenuraarchianassaporioni* Lemaire, 1980, known from western Ecuador (Manabí province), is expected in southwestern Colombia (Na) (Brechlin 2023b).

*Automerisarminandensis* Brechlin & Käch, 2017 was described from two specimens collected in Ecuador (Orellana) and Peru (Loreto) (Brechlin et al. 2017: 71). But there is an additional (female) specimen in CRBP (BC-HKT 0225) from the Ecuadorian province of Sucumbíos very near the border to Putumayo. Therefore, its occurrence is expected in southeastern Colombia.

*Automerisbarragani* Brechlin, Käch & Meister, 2013 was described from specimens collected in Ecuador (Carchi), very near the border to Nariño (Brechlin et al. 2013b). Therefore, its occurrence is expected in southern Colombia.

*Automerissachai* Brechlin, Käch & Meister, 2013 was described from specimens collected in Ecuador (Carchi), very near the border to Nariño (Brechlin et al. 2013b). Therefore, its occurrence is expected in southern Colombia.

*Copaxakaechi* Brechlin & Meister, 2013 was described from specimens collected in Ecuador (Carchi), near the border to Nariño (Brechlin et al. 2013d). Therefore, its occurrence is expected in southern Colombia.

*Copaxatulcana* Brechlin, 2016 was described from specimens collected in Ecuador (Carchi), very near the border to Nariño (Brechlin et al. 2016b). Therefore, its occurrence is expected in southern Colombia.

*Eaclesalinae* Brechlin & Käch, 2015 was described from specimens collected in Ecuador (Napo) at low elevations. Therefore, its occurrence is expected in southern Colombia.

*Erythromeriskaechi* Brechlin, 2016 was described from specimens collected in Ecuador (Carchi), very near the border to Nariño (Brechlin 2016b). Therefore, its occurrence is expected in southern Colombia.

*Gameliarindgei* Lemaire, 1967 was reported for the Amazon region of eastern Ecuador and northern Peru (Lemaire 2002). Therefore, its occurrence is expected in the Colombian Amazon.

*Hyperchiriaparda* Brechlin, Käch & Meister, 2011 was described from specimens collected in Ecuador (Tungurahua) (Brechlin et al. 2011b). Therefore, its occurrence is expected in southern Colombia.

*Loxolomiajohnsoni* Schaus, 1932, known from northern Peru (Loreto and Amazonas departments) to Bolivia, is expected at least in the far southeast of Colombia (Am).

Meroleuca (Meroleucoides) erythropus (Maassen, 1890) could be expected in southern Colombia as it occurs in the north-Ecuadorian province of Carchi (e.g., BC-RBP 7085) very near the border with Na.

Meroleuca (Meroleucoides) kaechi Brechlin & Meister, 2013 was described from specimens collected in Ecuador (Carchi), near the border to Nariño. Therefore, its occurrence is expected in southern Colombia (Brechlin et al. 2013c).

*Pseudodirphiaecoridides* Brechlin, Meister & Käch, 2011 was reported for the Amazon region of eastern Ecuador (Brechlin and Meister 2011g) and northern Peru. Therefore, its occurrence is expected in the Colombian Amazon.

*Rhescyntisdescimoni* Lemaire, 1975 was reported for the Amazon region of Ecuador (Napo) (Racheli and Racheli 2006) and Peru (San Martín). Therefore, its occurrence should be expected in the Colombian Amazon.

### ﻿Notes on the previously published checklists of the Colombian Saturniidae

The aim of this study is to show the taxonomic richness of the Saturniidae fauna of Colombia. During this work, we were becoming aware that there were still many taxonomical problems to solve, as well as a large number of unmounted specimens in the collection of the second author (CRBP), including some undescribed taxa (Brechlin [et al.] 2021–2023). For instance, there are old records of Eacles (imperialis) cacicus (Boisduval, 1868), with its type locality in the Brazilian state of Bahía, for several eastern Colombian departments (e.g., Lemaire 1988b: 38). This taxon is treated as a synonym of *E.magnifica* Walker, 1855 now Brechlin (Brechlin 2022i) and the replacement name for the eastern Colombian populations is *E.impandensis*. Because of the importance of such taxonomical acts and the long time it took, we decided to publish a preprint to make our preliminary results readily available (Comoglio and Brechlin 2021). After this occurred on 6 August 2021, another checklist of the Saturniidae of Colombia by Jiménez-Bolívar et al. (2021) was published on 10 December 2021 on Zootaxa. Because of the confusion in counting the listed taxa of Colombian Saturniidae in the checklists by Amarillo-Suárez (2000) and Jiménez-Bolívar et al. (2021), it is now necessary to present the correct numbers (Table 3).

**Table 3.** Numbers of genera, species, and taxa within subfamilies of Saturniidae in Colombia reported by Amarillo-Suárez (2000), in the preprint of this study, by Jiménez-Bolívar et al. (2021), and in the current study.

Subfamily	Amarillo-Suárez (2000)	Preprint of this study (08/2021)	Jiménez-Bolívar et al. (12/2021)	This study (2023)
Arsenurinae	8/18/18	8/28/32	8/27/33	8/35/38
Ceratocampinae	14/36/36	15/79/82	15/82/84	15/90/92
Cercophaninae	-	1/52/52	1/52/52	1/81/81
Hemileucinae	20/109/109	24/362/369	24/387/394	24/467/478
Hirpidinae	-	1/8/8	1/8/8	1/8/8
Oxyteninae	-	3/22/24	3/24/28	3/29/31
Saturniinae	3/20/20	3/51/54	3/52/54	3/56/62
**Total**	**45/183/183**	**55/602/621**	**55/632/653**	**55/766/790**

The difference in comparison with the paper by Amarillo-Suárez (2000) is because herein the genus *Hyperchiria* Hübner, 1819 [1816] and the species *Hyperchirianausica* (Cramer, 1779) and *Rhodirphiacarminata* (Schaus, 1902) were listed (and counted) twice (Amarillo-Suárez 2000: 184 f). That is why “a total of 185 species, distributed in 46 genera” were listed in Amarillo-Suárez (2000: 177 ff).

In Table 1 in Jiménez-Bolívar et al. (2021: 155), these authors stated to report the “No of genera / spp. and sspp.” but for the preprint study by Comoglio and Brechlin (2021), they only gave the number of species, but not of all taxa. In our preprint we presented a total number of 602 species, counting e.g., *Arsenuraarchianassaarchianassa* and *A.a.porioni* as a single species; thus, the direct comparison is misleading. The correct number of all listed taxa (sp. and ssp.) in Comoglio and Brechlin (2021) is indeed 621 as shown in Table 3.

In this context, it is also worth mentioning that 385 (59%) of the 653 listed taxa in Jiménez-Bolívar et al. (2021) have been described by Brechlin et al. Furthermore, Jiménez-Bolívar et al. (2021) have used 338 “evidence” (e.g., BC-RBP, BC-FMP, BC-EvS) or single “source(s)” (in summary, 52%) from the studies of the working group of the second author (mainly with Viktor Sinyaev, Frank Meister, Eric van Schayck, Horst Käch, Jan-P. Rudloff, and Peggy Rimkus-Handschug [Ackermann]). In some genera, these comprehensive studies by this working group were used by Jiménez-Bolívar et al. (2021) up to 100% as, e.g., all their evidence in *Janiodes*. In some genera, such as *Gamelioides* (100%), *Gamelia* (33 of 38 [87%]), *Hirpida* (7 of 8 [87%]), and *Copaxa* (25 of 39 [64%]), the majority of the known Colombian taxa were described by Brechlin et al., all this “based mainly on a literature review” (Jiménez-Bolívar et al. 2021: 153).

In addition, it should be mentioned that the following 13 taxa listed in the checklist by Jiménez-Bolívar et al. (2021) seem to be unlikely distributed in Colombia as previously discussed: *Automerislapaza*, *Automerislarra*, *Automerismoresca*, *Cerodirphiaaraguensis*, *Citheronialaocoon*, *Hylesiacoex*, *Hyperchiriaacuta*, *Leucanellayungasensis*, *Perigabispinosa*, *Pseudodirphiaperuviana*, *Oxytenisnaemiaorecta*, *Theriniatransversariatransversaria*, and *Rothschildiaperuvianaperuviana*.

Furthermore, we are concerned about the records extending some species’ distributional ranges. Taxa identification in cryptic species complexes cannot be easily accomplished through a photograph. For instance, there is still a large number of Saturniidae species reported in “iNaturalist” for Colombia, which are definitely not distributed in the country: e.g., *Automeriscecrops*, *Automerisio*, *Copaxaherbuloti*, *Molipparivulosa*, *Rothschildiatriloba*, and others. These records correspond to an incorrect identification assigned to the observations of the iNaturalist platform, which are mostly unverified or tentatively carried out by inexperienced users. For example, this is the case of the records of *Copaxaantiollita* for Ma (https://www.inaturalist.org/observations/54286917) and St (https://www.inaturalist.org/observations/20981043) reported by Jiménez-Bolívar et al. (2021: 194) that likely refer to *C.winbrechliniani* and *C.satellita*, respectively. Exceptions can only be made for those species whose identity is clearly visible by external morphological features (e.g., *Antheraeagodmanicolumbiana*, *Copaxasapatoza*, or *Rothschildiazacateca*).

Finally, the correct authorship of *Janiodesvirgata* is Jordan, 1924 and not Brechlin, 2020 as reported by Jiménez-Bolívar et al. (2021: 168). The correct authorship of *Pseudautomerischocensis* and *P.horsti* is Brechlin & Meister, 2013 instead of Brechlin, Käch & Meister, 2013 as reported in Jiménez-Bolívar et al. (2021: 157) and our preprint, as well as the correct authorship of *Pseudodirphiaobecuatoriana* Brechlin, Meister & Käch, 2011 instead of Brechlin & Meister, 2011 as reported in Jiménez-Bolívar et al. (2021: 190) and unfortunately in our unreviewed preprint. In addition, the subspecific epithet of *Hylesiacontinuacolombiana* is misspelled as “columbiana” in both Jiménez-Bolívar et al. (2021: 179) and our preprint; and the specific epithet of *Janiodesrusbogotana* is misspelled as “rusbogatana” in Jiménez-Bolívar et al. (2021: 167).

### ﻿Taxonomic progress

**Figure 2.**
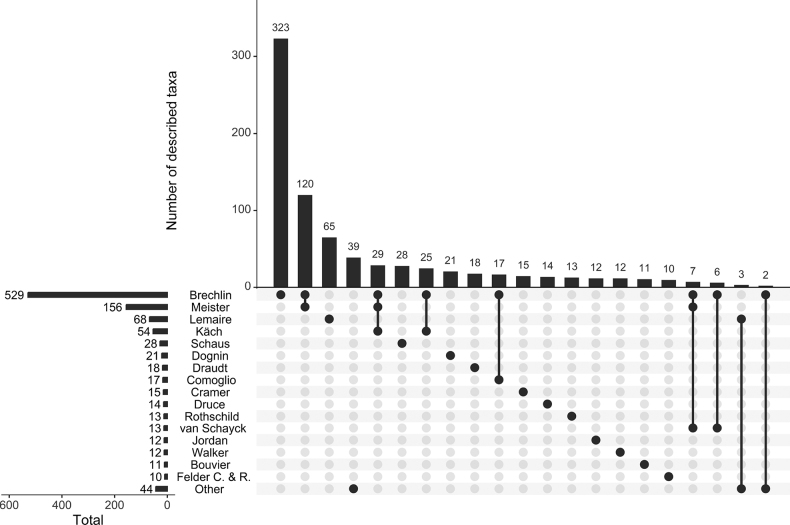
UpSet plot of the number of described Saturniidae species and subspecies that are distributed in Colombia by author or groups of authors. Data were retrieved from the “Taxon” column of Table 2. Those authors (*n* = 36) that have contributed fewer than 10 described taxa were grouped together as “Other”.

**Figure 3.**
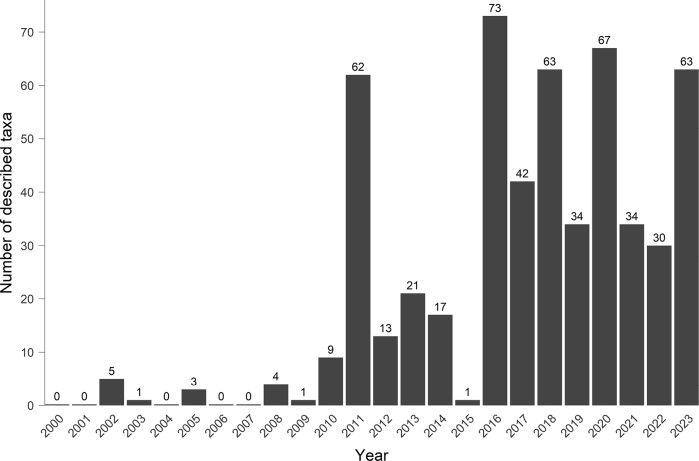
Bar chart of the number of described Saturniidae species and subspecies that are distributed in Colombia by year, starting from 2000. Data were retrieved from the “Taxon” column of Table 2.

The taxonomy of the Saturniidae has undergone a constant change and increase in the description of taxa on a global scale, with nearly 150 taxa described per year from 2008, when DNA barcoding began to be used to describe species (Decaëns and Rougerie 2008), to 2018 (Kitching et al. 2018). This effort is also evident for the Colombian Saturniidae, whose taxonomic progress has been especially promoted by the second author (RB) and his working group. Together, Brechlin et al. have described 529 taxa distributed in Colombia (Fig. 2), which is 67% of the total number of known taxa for the country. Since the publication of the first checklist of Colombian Saturniidae (Amarillo-Suárez 2000), there has been a spectacular increase in the number of species descriptions (Fig. 3), with the description of 543 taxa that make up 69% of the currently known taxa for the country. In the last decade (2013–2023), on average, 40 Saturniidae taxa distributed in Colombia have been described annually. It should also be noted that an impressive number of 93 taxa has been described since the publication of this study’s preprint (Comoglio and Brechlin 2021).

### ﻿Diversity and endemism

While diurnal butterflies (Lepidoptera: Papilionoidea) have been studied to a greater extent in Colombia, achieving to list 3,877 species, of which 219 are endemic (Garwood et al. 2022), little is known about the richness of moth species in the country. Checklists of moths have recently been published for a few families. In Colombia, 188 species of Sphingidae (Correa-Carmona et al. 2015), at least 177 species of Geometridae (Murillo-Ramos et al. 2021), 53 species of Pterophoridae (Landry and Gielis 2022), and 515 species of Notodontidae, of which 51 are endemic (Prada-Lara et al. 2023), have been reported. Therefore, the Saturniidae are currently Colombia’s most diverse documented family of moths, besides presenting the highest number and rate of endemic species. However, the diversity of some families, such as Erebidae and Geometridae, which is extremely high in the world (van Nieukerke et al. 2011) and the Neotropic (Pitkin 2002; Vincent and Laguerre 2014; Murillo-Ramos et al. 2021), is currently tremendously underestimated in Colombia and the Neotropic, and could significantly exceed the richness of Saturniidae in the country. It was already anticipated by Lemaire and Venedictoff (1989: 2) that “only Colombia, which has a geographical situation comparable with Ecuador, (with much more complexity in the cordilleras), may support a larger fauna” of Saturniidae, and finally, this study makes Colombia the most diverse documented country in the world for this family.

Colombia’s best-known regions regarding Saturniidae diversity include the Pacific and Andean regions. The most speciose subfamily in Colombia is Hemileucinae, and it is not surprising that many species and even a genus of this subfamily are endemic and were recently described. Most endemism are high Andean species with very narrow distribution ranges, mainly members of the genera *Automeris* of the *alticola* group, *Copaxa* of the *sapatoza* (*semioculata*) group, *Gamelioides*, *Janiodes*, and *Meroleuca*. A great endemic diversity of Colombian Saturniidae can be studied in montane biotopes such as high Andean forests and páramos. For example, the genus *Meroleuca* comprises 30 species in Colombia, almost all endemic. This data confirms a hypothesis by Lemaire that years before the intensive sampling in the Neotropic predicted that new species of *Meroleuca* “are expected every time a new collecting site is sampled at about 2500 m elevation or more” (Lemaire 2002: 14). The distribution of the endemic species of Saturniidae in Colombia demonstrates the importance of prioritizing the conservation of paramo and high Andean habitats, where the true richness of unique species for the country is concentrated. On the other hand, it should be noted that many Colombian endemic Saturniidae are classified as such at the moment because they are only known from their type locality. For instance, *Dirphiaradinirida* and *Pseudodirphialeticiana* are expected to be found in the neighboring Amazonian countries in the future.

Few species can be considered truly polytopic, with a wide distribution range. All of these taxa occur in lowlands and have not been reported for elevations higher than 1,500 meters. A short list of polytopic taxa includes *Arsenuraciocolatina*, *Caiochampioni*, *Titaeatamerlan*, *Adeloneivaiaboisduvalii*, *A.pallida*, *Syssphinxquadrilineata*, *Automerisargentifera*, Hylesia (Hylesia) continua, Hylesia (Micrattacus) nanus, *Lonomiavenezuelensis*, *Hirpidagaujoni*, *Rothschildialebeauaroma*, and *Copaxatroetschi*. Furthermore, the vast majority of these taxa have not been recently described, so records of their distribution have accumulated in the literature. In contrast, many recently described taxa are known only from their type locality, but their distribution could be expanded through increased sampling efforts. A recent example of this is the distribution of *Antheraeagodmanicolumbiana*, which was recently found in the southern department of Caquetá. In contrast, previous records showed that its distribution was limited to Antioquia and Santander, which is surprising considering that it is a relatively highland species (Ramos-Artunduaga et al. 2022). Other highland species that, until the checklist by Amarillo-Suárez (2000), were believed to be limited to the Eastern Cordillera are *Bathyphlebiaaglia* and *Erythromerissaturniata*, which today are known to be distributed in both Eastern and Central Cordillera. *Automerisiwanowitschi*, a highland species of the *alticola*-species group, described from Ecuador, was initially found only in the south of the country in the Central Cordillera, but was later found in Antioquia, due to additional sampling efforts.

## ﻿Conclusions

Many Saturniidae species have been recently described from Colombia. This study is the most recent attempt to present a checklist containing all the new descriptions and updated distribution data of all Colombian Saturniidae taxa. Most of these records are available on BOLD repository which has been used as both a tool for taxonomists (e.g., describing new species) and a source of occurrence data for each species. This comprehensive checklist of the Saturniidae of Colombia includes 790 taxa (766 in species rank) within 55 genera in 7 subfamilies, for which an updated taxonomic key is provided. According to available distribution data, the genus *Winbrechlinia*, the subgenusDarylesia, 379 species, and 18 subspecies are endemic to Colombia. Several old records and some species names given in the checklist by Amarillo-Suárez (2000) were discussed if excluded from this present checklist due to new studies and evidence. This checklist aims to avoid confusion with old names and provide an updated list of Colombian Saturniidae species. It is expected that this work will also become a useful tool for identification based on the biogeographic distribution of the species. The most recently described species (as of 15 June 2023), together with their distribution data, are included. A review and update of the taxonomy of the Colombian Saturniidae taxa were carried out, including some critical taxonomic changes, proposing synonymies and revalidations of taxa. Future studies and sampling in the Colombian lowlands should reveal additional species that are expected in Colombia as they have already been reported from the neighboring countries. Nevertheless, this checklist and the remarkable diversity of Colombian saturniid moths emphasizes the status of Colombia as an outstanding reference country for studying moth diversity and as the richest documented country in the world for Saturniidae diversity.

## ﻿Additional information

### Data availability

All of the data that support the findings of this study are available in the main text.
